# Supplementary material for: The anti-PD-L1/CTLA-4 bispecific antibody KN046 in combination with nab-paclitaxel in first-line treatment of metastatic triple-negative breast cancer: a multicenter phase II trial
Source: Nat Commun. 2024 Feb 3;15:1015. doi: 10.1038/s41467-024-45160-y (PMC10838317; doi:10.1038/s41467-024-45160-y)
Supplement: Supplementary file 1 — Supplementary Information [file 41467_2024_45160_MOESM1_ESM.pdf]

Supplementary information

Supplement to: Qiao Li, et al. The anti-PD-L1/CTLA-4 bispecific antibody KN046 in combination with nab-paclitaxel in first-line treatment of metastatic triple-negative breast cancer: a multicenter phase II trial.

Table of contents

Supplementary Table 1. Best overall response assessed by investigators…...2
Supplementary Table 2. Survival outcomes……………………………………….3
Supplementary Table 3. irAEs……………………………………………………...5
Supplementary Note. Study protocol……………………………………………..10

Supplementary Table 1. Best Overall Response assessed by investigators

	KN046 3mg/kg Q2W + nab-paclitaxel (n=15)	KN046 5mg/kg Q2W + nab-paclitaxel (n=10)	Total* (n=25)
Best Overall Response, *n* (%)			
Complete Response, CR	2 (13.3%)	0	2 (8.0%)
Partial Response, PR	4 (26.7%)	3 (30.0%)	7 (28.0%)
Stable Disease, SD	9 (60.0%)	6 (60.0%)	15 (60.0%)
Progressive Disease, PD	0	1 (10.0%)	1 (4.0%)
Objective Response Rate, ORR (95%CI)	40.0%	30.0%	36.0%
[CR+PR]	(16.3% - 67.7%)	(6.7% - 65.3%)	(18.0% - 57.5%)
Disease Control Rate, DCR (95%CI)	100.0%	90.0%	96.0%
[CR+PR+SD]	(78.2% - 100.0%)	(55.5% - 99.8%)	(79.7% - 99.9%)
Clinical Benefit Rate, CBR (95%CI)	60.0%	40.0%	52.0%
[CR+PR+SD≥24 weeks]	(32.3% - 83.7%)	(12.2% - 73.8%)	(31.3% - 72.2%)
Median DoR (95%CI)	Not reached	11.79 months (5.59, not reached)	11.93 months (5.59, not reached)

*2 patients withdrew from the trial before the first tumor assessment and were excluded from the evaluable population.

95%CI, 95% confidence interval.

Supplementary Table 2 - survival outcomes

	3 mg/kg Q2W (N = 16)	5 mg/kg Q2W (N = 11)	Total (N = 27)
median PFS	8.61 months	3.65 months	7.33 months
95%CI	(3.71, -)	(1.61, 9.10)	(3.68, 11.07)
1-year PFS rate	39.68%	10.00%	26.18%
95%CI	(14.78, 63.96)	(0.57, 35.81)	(10.37, 45.26)
2-year PFS rate	39.68%	-	26.18%
95%CI	(14.78, 63.96)	-	(10.37, 45.26)

	3 mg/kg Q2W (N = 16)	5 mg/kg Q2W (N = 11)	Total (N = 27)
median OS	- months	27.73 months	30.92 months
95%CI	(8.61, -)	(6.01, -)	(14.75, -)
1-year OS rate	80.00%	62.50%	73.91%
95%CI	(49.98, 93.07)	(22.93, 86.07)	(50.92, 87.34)
2-year OS rate	58.18%	62.50%	60.05%
95%CI	(29.41, 78.69)	(22.93, 86.07)	(37.17, 76.90)

	3 mg/kg Q2W (N = 16)	5 mg/kg Q2W (N = 11)	Total (N = 27)
TC < 1%			
median PFS	6.98 months	3.68 months	4.73 months
95%CI	(3.68, -)	(3.58, 11.07)	(3.61, 11.07)
TC ⩾ 1%			
median PFS	- months	2.66 months	8.61 months
95%CI	(7.33, -)	(1.61, -)	(1.61, -)
HR (95%CI)	0.5<br>(0.09, 2.77)	2.3<br>(0.52, 9.73)	0.8<br>(0.27, 2.35)
P-value	0.4286	0.2762	0.6817
TC < 1%			
median OS	16.23 months	30.92 months	30.92 months
95%CI	(4.73, -)	(6.01, -)	(6.01, -)
TC ⩾ 1%			
median OS	- months	17.05 months	26.14 months
95%CI	(8.61, -)	(9.56, -)	(8.61, -)
HR (95%CI)	0.7<br>(0.14, 3.54)	3.5<br>(0.48, 25.27)	1.1<br>(0.34, 3.62)
P-value	0.6731	0.2144	0.8717

HR, hazards ratio; 95%CI, 95% confidence interval; PFS, progression-free survival; OS, overall survival.

Survival analysis based on Kaplan-Meier method was used for PFS, and OS. Log-rank test was used to compare the difference in survival rate based on PD-L1 expression status. All p-values were two-sided.

Supplementary Table 3 - irAEs			
n (%)	3 mg/kg Q2W (N = 16)	5 mg/kg Q2W (N = 11)	Total (N = 27)
	8 (50.0)	5 (45.5)	13 (48.1)
Grade 1	5 (31.3)	0	5 (18.5)
Grade 2	3 (18.8)	2 (18.2)	5 (18.5)
Grade 3	0	3 (27.3)	3 (11.1)
Grade 4	0	0	0
Grade 5	0	0	0
Grade 3/4	0	3 (27.3)	3 (11.1)
Grade ≥3	0	3 (27.3)	3 (11.1)
elevated ALT	1 (6.3)	0	1 (3.7)
Grade 1	1 (6.3)	0	1 (3.7)
Grade 2	0	0	0
Grade 3	0	0	0
Grade 4	0	0	0
Grade 5	0	0	0
Grade 3/4	0	0	0
Grade ≥3	0	0	0
elevated AST	1 (6.3)	0	1 (3.7)
Grade 1	1 (6.3)	0	1 (3.7)
Grade 2	0	0	0
Grade 3	0	0	0
Grade 4	0	0	0
Grade 5	0	0	0
Grade 3/4	0	0	0
Grade ≥3	0	0	0
elevated γ-GGT	0	1 (9.1)	1 (3.7)
Grade 1	0	0	0
Grade 2	0	0	0
Grade 3	0	1 (9.1)	1 (3.7)
Grade 4	0	0	0
Grade 5	0	0	0
Grade 3/4	0	1 (9.1)	1 (3.7)
Grade ≥3	0	1 (9.1)	1 (3.7)
elevated T3	1 (6.3)	0	1 (3.7)
Grade 1	1 (6.3)	0	1 (3.7)
Grade 2	0	0	0
Grade 3	0	0	0
Grade 4	0	0	0
Grade 5	0	0	0
Grade 3/4	0	0	0
Grade ≥3	0	0	0

gain weight	1 (6.3)	0	1 (3.7)
Grade 1	0	0	0
Grade 2	1 (6.3)	0	1 (3.7)
Grade 3	0	0	0
Grade 4	0	0	0
Grade 5	0	0	0
Grade 3/4	0	0	0
Grade ⩾3	0	0	0
thrombocytopenia	0	0	0
Grade 1	0	0	0
Grade 2	0	0	0
Grade 3	0	0	0
Grade 4	0	0	0
Grade 5	0	0	0
Grade 3/4	0	0	0
Grade ⩾3	0	0	0
decreased free thyroxine	1 (6.3)	0	1 (3.7)
Grade 1	1 (6.3)	0	1 (3.7)
Grade 2	0	0	0
Grade 3	0	0	0
Grade 4	0	0	0
Grade 5	0	0	0
Grade 3/4	0	0	0
Grade ⩾3	0	0	0
hypothyroidism	2 (12.5)	1 (9.1)	3 (11.1)
Grade 1	2 (12.5)	0	2 (7.4)
Grade 2	0	1 (9.1)	1 (3.7)
Grade 3	0	0	0
Grade 4	0	0	0
Grade 5	0	0	0
Grade 3/4	0	0	0
Grade ⩾3	0	0	0
hyperthyroidism	0	1 (9.1)	1 (3.7)
Grade 1	0	1 (9.1)	1 (3.7)
Grade 2	0	0	0
Grade 3	0	0	0
Grade 4	0	0	0
Grade 5	0	0	0
Grade 3/4	0	0	0
Grade ⩾3	0	0	0
immune-mediated thyroiditis	0	1 (9.1)	1 (3.7)
Grade 1	0	0	0
Grade 2	0	1 (9.1)	1 (3.7)

Grade 3	0	0	0
Grade 4	0	0	0
Grade 5	0	0	0
Grade 3/4	0	0	0
Grade ≥3	0	0	0
infusion-related reaction	2 (12.5)	0	2 (7.4)
Grade 1	1 (6.3)	0	1 (3.7)
Grade 2	1 (6.3)	0	1 (3.7)
Grade 3	0	0	0
Grade 4	0	0	0
Grade 5	0	0	0
Grade 3/4	0	0	0
Grade ≥3	0	0	0
immune-mediated liver disease	0	2 (18.2)	2 (7.4)
Grade 1	0	0	0
Grade 2	0	0	0
Grade 3	0	2 (18.2)	2 (7.4)
Grade 4	0	0	0
Grade 5	0	0	0
Grade 3/4	0	2 (18.2)	2 (7.4)
Grade ≥3	0	2 (18.2)	2 (7.4)
abnormal liver function	1 (6.3)	0	1 (3.7)
Grade 1	1 (6.3)	0	1 (3.7)
Grade 2	0	0	0
Grade 3	0	0	0
Grade 4	0	0	0
Grade 5	0	0	0
Grade 3/4	0	0	0
Grade ≥3	0	0	0
peripheral edema	1 (6.3)	0	1 (3.7)
Grade 1	0	0	0
Grade 2	1 (6.3)	0	1 (3.7)
Grade 3	0	0	0
Grade 4	0	0	0
Grade 5	0	0	0
Grade 3/4	0	0	0
Grade ≥3	0	0	0
pyrexia	1 (6.3)	0	1 (3.7)
Grade 1	1 (6.3)	0	1 (3.7)
Grade 2	0	0	0
Grade 3	0	0	0
Grade 4	0	0	0
Grade 5	0	0	0

Grade 3/4	0	0	0
Grade ≥3	0	0	0
hypoalbuminemia	1 (6.3)	0	1 (3.7)
Grade 1	1 (6.3)	0	1 (3.7)
Grade 2	0	0	0
Grade 3	0	0	0
Grade 4	0	0	0
Grade 5	0	0	0
Grade 3/4	0	0	0
Grade ≥3	0	0	0
hypokalemia	1 (6.3)	0	1 (3.7)
Grade 1	1 (6.3)	0	1 (3.7)
Grade 2	0	0	0
Grade 3	0	0	0
Grade 4	0	0	0
Grade 5	0	0	0
Grade 3/4	0	0	0
Grade ≥3	0	0	0
encephalitis	0	0	0
Grade 1	0	0	0
Grade 2	0	0	0
Grade 3	0	0	0
Grade 4	0	0	0
Grade 5	0	0	0
Grade 3/4	0	0	0
Grade ≥3	0	0	0
bronchitis	0	0	0
Grade 1	0	0	0
Grade 2	0	0	0
Grade 3	0	0	0
Grade 4	0	0	0
Grade 5	0	0	0
Grade 3/4	0	0	0
Grade ≥3	0	0	0
allergic dermatitis	1 (6.3)	0	1 (3.7)
Grade 1	0	0	0
Grade 2	1 (6.3)	0	1 (3.7)
Grade 3	0	0	0
Grade 4	0	0	0
Grade 5	0	0	0
Grade 3/4	0	0	0
Grade ≥3	0	0	0

rash	0	1 (9.1)	1 (3.7)
Grade 1	0	0	0
Grade 2	0	0	0
Grade 3	0	1 (9.1)	1 (3.7)
Grade 4	0	0	0
Grade 5	0	0	0
Grade 3/4	0	1 (9.1)	1 (3.7)
Grade ⩾3	0	1 (9.1)	1 (3.7)
dorsalgia	1 (6.3)	0	1 (3.7)
Grade 1	0	0	0
Grade 2	1 (6.3)	0	1 (3.7)
Grade 3	0	0	0
Grade 4	0	0	0
Grade 5	0	0	0
Grade 3/4	0	0	0
Grade ⩾3	0	0	0
immune-mediated enterocolitis	1 (6.3)	0	1 (3.7)
Grade 1	0	0	0
Grade 2	1 (6.3)	0	1 (3.7)
Grade 3	0	0	0
Grade 4	0	0	0
Grade 5	0	0	0
Grade 3/4	0	0	0
Grade ⩾3	0	0	0
diabetes mellitus	0	0	0
Grade 1	0	0	0
Grade 2	0	0	0
Grade 3	0	0	0
Grade 4	0	0	0
Grade 5	0	0	0
Grade 3/4	0	0	0
Grade ⩾3	0	0	0
adrenal insufficiency	0	0	0
Grade 1	0	0	0
Grade 2	0	0	0
Grade 3	0	0	0
Grade 4	0	0	0
Grade 5	0	0	0
Grade 3/4	0	0	0
Grade ⩾3	0	0	0

Supplementary Note.
Study protocol

CLINICAL TRIAL PROTOCOL

COMPOUND: KN046

A Phase Ib/II Study to Evaluate Efficacy, Safety and Tolerability of KN046 Monotherapy or in Combination with Nab-paclitaxel in Subjects with Triple-negative Breast Cancer

STUDY NUMBER: KN046-203

VERSION NUMBER: 1.2

VERSION DATE: 25/01/2022

Sponsor Signature Page

I have read this clinical trial protocol, protocol No.: KN046-203, version No.: 1.2 (version date: 25 Jan. 2022), and I agree to perform relevant duties in accordance with the Local laws, the Declaration of Helsinki, the ICH-GCP and this study protocol. This study can only be conducted after approval by the Ethics Committee.

During the study, I will strictly follow the requirements of the protocol. If necessary, the protocol can only be revised after the consent of the sponsor and after the reapproval or filing by the Ethics Committee, unless measures must be taken to protect the safety, rights, and interests of the subjects.

I will keep the protocol and the related contents confidential.

Sponsor: Jiangsu Alphamab Biopharmaceuticals Co., Ltd.

Medical leader (printed):

Signature: ______________________________

Date of Signature:
(dd/Mmm/yyyy) ______________________________

Investigator Signature Page

I have read this clinical trial protocol, protocol No.: KN046-203, version No.: 1.2 (version date: 25 Jan. 2022), and I agree to perform relevant duties in accordance with the Local laws, the Declaration of Helsinki, the ICH-GCP and this study protocol. This study can only be conducted after approval by the Ethics Committee.

During the study, I will strictly follow the requirements of the protocol. If necessary, the protocol can only be revised after the consent of the sponsor and after the reapproval or filing by the Ethics Committee, unless measures must be taken to protect the safety, rights and interests of the subjects.

I will keep the protocol and the related contents confidential.

Clinical study institution:

Principal investigator (printed):

Principal investigator (printed):

Signature: ____

Date of Signature:
(dd/Mmm/yyyy) ____

Signature: ____

Date of Signature:
(dd/Mmm/yyyy) ____

CLINICAL TRIAL SYNOPSIS

COMPOUND: KN046 (a humanized PD-L1/CTLA4 bispecific single domain Fc fusion protein antibody)

STUDY No.: KN046-203

TITLE: A Phase Ib/II Study to Evaluate Efficacy, Safety and Tolerability of KN046 Monotherapy or in Combination with Nab-paclitaxel in Subjects with Triple-negative Breast Cancer

INVESTIGATOR/TRIAL LOCATION: China

PHASE OF DEVELOPMENT: Phase Ib/II

STUDY OBJECTIVE(S)

Primary objective:

- To evaluate the anti-tumor activity of KN046 monotherapy.
- To evaluate the anti-tumor activity of combination of KN046 and nab-paclitaxel.

Secondary objective(s):

- To evaluate the safety and tolerability of KN046 monotherapy.
- To evaluate the safety and tolerability of the KN046 combination therapy.
- To evaluate the immunogenicity of KN046.
- To characterize the pharmacokinetics of KN046 and evaluate the effect of nab-paclitaxel on the pharmacokinetics of KN046.
- To evaluate the impact of biomarkers (PD-L1 expression, BRCA1/2 mutations, HRD, TMB, TIL) on the anti-tumor activity of KN046.
- To evaluate the correlation between drug exposure levels and anti-tumor activity of KN046.

Exploratory objective:

- To explore the correlation between KN046 drug exposure and safety.

STUDY DESIGN:

This study is a Ib/II, multicenter, open-label clinical trial designed to evaluate the efficacy and safety of KN046 monotherapy or combination with nab-paclitaxel in patients with metastatic or locally advanced unresectable triple-negative breast cancer (TNBC) in China. The study will consist of two stages, does-escalation stage, and dose-expansion stage. Each subject will submit tumor tissue samples to determine tumor and immune cell biomarkers such as BRCA1/2 mutation status, HRD mutation status, tumor mutational burden (TMB), PD-L1 expression level, and TIL.

Subjects who have received at least one prior line of systemic treatment will receive KN046 monotherapy. The monotherapy cohort will initially enroll 6 subjects and allocated to 3 mg/kg IV Q2W according to the protocol requirements. After 6 subjects have been enrolled and have completed a 28-day safety observation period, a Safety Monitoring Committee (SMC) meeting will be held (enrollment will not be paused during this period). The safety, preliminary efficacy, and pharmacokinetic data of KN046 will be reviewed, and the following decisions will be made: 1) expand the 3 mg/kg dose group to 30 subjects; and/or 2) dose escalates to 5 mg/kg IV Q2W treatment group (subjects in the 3 mg/kg Q2W cohort who do not experience Grade ≥ 2 treatment-related adverse events before the SMC meeting can escalate to 5 mg/kg IV Q2W with the consent of the investigator and subjects). The 5 mg/kg IV Q2W cohort will initially enroll 6 subjects. After 6 subjects have been enrolled and have completed a 28-day safety observation period, an SMC meeting will be held (enrollment will not be paused during this period). The safety, preliminary efficacy, and pharmacokinetic data of KN046 will be reviewed, and a decision will be made on whether to continue expanding the 5 mg/kg dose group to 30 subjects (Figure 1). Each subject will receive KN046 (predefined dosing regimen is 3 mg/kg or 5 mg/kg IV, Q2W), until disease progression (assessed by the investigator based on RECIST1.1), intolerable toxicity, withdrawal of consent, or completion of 2-years treatment, whichever occurs first. After each cohort of the KN046 monotherapy has enrolled 30 subjects and all subjects have completed at least one post-baseline imaging, an interim analysis will be conducted. After discussion by the Scientific Monitoring Committee (SMC), a decision will be made on whether to further expand in the original cohort or in the enriched population of biomarker-positive individuals or explore higher dose levels and/or different dosing intervals.

Subjects naïve to systemic anti-cancer treatment will receive combination therapy with KN046 and nab-paclitaxel. The combination treatment cohort will initially enroll 6 subjects, who will receive KN046 at a dose of 3 mg/kg IV Q2W in combination with albumin-bound paclitaxel as per the protocol. After all 6 subjects have been enrolled and have completed a 28-day safety observation period, an SMC meeting will be held (enrollment will not be paused during this period). The safety, preliminary efficacy, and pharmacokinetic data of KN046 will be reviewed, and the following decisions will be made: 1) expand the 3 mg/kg dose group to 30 subjects; and/or 2) dose escalates to KN046 5 mg/kg IV Q2W treatment group (subjects in the 3 mg/kg Q2W cohort who do not experience Grade ≥ 2 treatment-related adverse events before the SMC meeting can escalate to 5 mg/kg IV Q2W with the consent of the investigator and subjects). The 5 mg/kg IV Q2W cohort will initially enroll 6 subjects. After 6 subjects have been enrolled and have completed a 28-day safety observation period, an SMC meeting will be held (enrollment will not be paused during this period). The safety, preliminary efficacy, and pharmacokinetic data of combination therapy will be reviewed, and a decision will be made on whether to continue expanding the KN046 5 mg/kg dose group to 25 subjects (Figure 2). Each subject will receive KN046 (predefined dosing regimen is 3 mg/kg or 5 mg/kg IV, d1,15, Q4W) and nab- paclitaxel (100 mg/m^2, d1,8,15, Q4W), until disease progression (assessed by the investigator based on RECIST1.1), intolerable toxicity, withdrawal of consent, or completion of 2-years treatment, whichever occurs first. The nab-paclitaxel is prescribed for 6 cycles (28-day/cycle) initially. After subjects completing these 6 cycles, investigators will evaluate the need for ongoing treatment with nab-paclitaxel based on medical best practices and a thorough assessment of the potential benefits and risks. After each cohort of the KN046 combination therapy has enrolled 25 subjects and all subjects have completed at least one post-baseline imaging, an interim analysis will be conducted. After discussion by the SMC, a decision will be made on whether to further expand in the original cohort or in the enriched population of biomarker-positive individuals or explore higher dose levels and/or different dosing intervals.

Tumor evaluation will be performed at baseline, every 8 weeks (56-days ± 7-days) within 12 months and every 12 weeks (84-days ± 7-days) thereafter. Tumor evaluation will continue until confirmed progressive disease per RECIST 1.1, starting new anti-cancer therapy, withdrawal of informed consent, or subject dies whichever comes first. Once objective response is observed, response should be confirmed by a second scan at approximately 8 weeks apart (no earlier than 4 weeks and no later than 8 weeks).

If a subject is clinically stable and has not experienced intolerable toxicity during KN046 treatment upon first RECIST 1.1 defined PD judged by the investigator, the subject is allowed to continue receiving treatment until confirmed disease progression (Section 6.1.3). Clinical stability is defined as: stable ECOG score, absence of unacceptable toxicity related to KN046 treatment (Section 6.1.4), absence of rapid disease progression requiring salvage therapy, and no emergent medical interventions required due to disease progression (such as central nervous system metastasis, tumor-induced airway obstruction leading to respiratory distress, or spinal cord compression). For subjects with bone metastases who were receiving denosumab during the screening period, it is required to switch to bisphosphonate to treat bone metastases before starting KN046 treatment.

An SMC will be established to review the emerging safety and efficacy data from this study. Based on safety, efficacy and/or pharmacokinetics data from this trial and/or other KN046-related trials, the SMC will decide whether to increase the intermediate KN046 dose, continue expansion in a certain dose and / or treatment cohort, terminate a certain dosing schedule and / or treatment cohort, add other dosing interval cohorts, and / or explore higher KN046 dosing based on safety, pharmacokinetics, and / or other data from previous treatment cohorts and / or other studies of KN046.

Each subject will receive study treatment as per the protocol until progressive disease (PD) as judged by the investigator per Response Evaluation Criteria in Solid Tumors (RECIST) V1.1, intolerable toxicity, withdrawal of consent by the subject, or treatment for 2 years, whichever occurs first.

The study period includes a screening period (Day -21 to Day 0), a treatment period (up to 2 years of treatment; if the investigator judges that the subject is still benefiting after 2 years, continuation of treatment will be allowed with the consent of the Sponsor), end of treatment follow-up (within 7 days after the decision to leave the group), 30-day safety follow-up, 90-day safety follow-up and long-term follow-up.

The clinical and laboratory assessment are detailed in Table 1 and 2, as well as Section 7.

Each subject will be asked for consent to participate in the non-mandatory biomarker assessment. Subjects who sign the consent (in either monotherapy or combination therapy) will be allocated to two groups. The first group of subjects will undergo fresh tumor biopsy at baseline and at week 2 after the first dose of KN046, while the second group of subjects will undergo fresh tumor biopsy

at baseline and at week 4 after the first dose of KN046, for the analysis of changes in tumor-infiltrating lymphocyte (TIL) before and after treatment (Section 4.1, 7.3.6.1).

STUDY POPULATION:

Inclusion criteria:

I01. Signed informed consent form.

I02. Male or female, 18 years of age or older; willing and able to complete all required procedures of study.

I03. Histology confirmed locally advanced unresectable or metastatic triple-negative breast cancer (TNBC):

- HER2-negative defined as (Wolff et al, 2018) [9]
  a. HER2 immunohistochemistry (IHC) 0 or 1+; or
  b. IHC 2+ and in situ hybridization (ISH) not amplified.
- ER-negative and PR-negative defined as IHC < 1%.

I04. Subjects' prior therapy needs to meet the requirement:

- KN046 monotherapy cohort: refractory to or relapsed after at least one prior therapeutic regimen for advanced/metastatic TNBC, prior exposure to a taxane either in localized or advanced/metastatic setting or neoadjuvant/adjuvant chemotherapy setting; subjects who refractory to or relapsed within 12 months after completion of neoadjuvant/adjuvant chemotherapy are considered as first-line treatment failure;
- KN046 plus nab-paclitaxel combination therapy cohort: systemic treatment naïve for advanced/metastatic TNBC; prior radiation therapy or endocrine therapy were allowed; subjects who completed neoadjuvant/adjuvant chemotherapy at least 12 months prior to the first dose of KN046.

I05. Baseline measurable disease according to RECIST 1.1 as determined by the local site investigator/radiology assessment. Target lesions situated in a previously irradiated area are considered measurable if progression has been demonstrated in such lesions.

I06. Have provided recently (within 2-years) or newly obtained core or excisional biopsy from a locally recurrent inoperable or metastatic tumor lesion for central determination of biomarker.

I07. ECOG performance status of 0 or 1 (Appendix 4).

I08. Adequate organ function assessed within 7 days prior to first trial treatment:

- Hematological function
  - ANC≥1.5 x 10^9/L;
  - Hemoglobin≥9 g/dL;
  - Platelets≥100 x 10^9/L.
- Renal function
  - Calculated creatinine clearance≥60 mL/min (Cockcroft-Gault method).
- Hepatic function
  - Total bilirubin≤1.5 x ULN (or 2.5 x ULN for documented Gilberts' syndrome);
  - ALT/AST≤3.0 x ULN (or 5.0 x ULN for documented liver metastasis).
- INR or aPTT ≤1.5 x ULN.
- TSH in the normal range; if TSH is abnormal, total T3 or free T3 and free T4 need to be within the normal range.

I09. Have a life expectancy of at least 3 months.

I10. If female of childbearing potential, have a negative serum pregnancy test within 7 days prior to first trial treatment.

I11. If female of childbearing potential or a male subject with a partner with childbearing potential, be willing to use a highly effective method of contraception (with a failure rate of less than 1.0% per year, please refer to Appendix 3) from first study treatment to 24 weeks after completion of the trial treatment.

Exclusion criteria:

E01. Leptomeningeal metastasis or untreated active CNS metastasis or leptomeningeal metastasis. Subjects with CNS metastasis may be eligible provided they are treated and clinically stable for at least 4 weeks and have no evidence of new or enlarging brain metastases and are off steroids 7 days for treating brain metastasis prior to first trial treatment.

E02. Untreated spinal compression fractures: treated spinal compression fractures require a minimum of 2 weeks of disease stability prior to the first dose.

E03. Uncontrolled hypercalcemia (corrected serum calcium concentration > 12 mg / dL or Ca^{2+} concentration > 1.5 mmol / L), or symptomatic hypercalcemia requires ongoing bisphosphonate therapy.

E04. Lactate dehydrogenase (LDH) > 2 x ULN.

E05. Uncontrolled cancer pain, analgesic drugs were not at a stable dose before enrollment.

E06. Is currently participating and receiving an investigational drug or has participated in a study of an investigational drug within 28 days.

E07. Has received other anti-tumor treatment within 28 days or within 5 times of half-life (no less than 2 weeks), whichever is shorter prior to the first trial treatment.

E08. Major surgery for any reason, except diagnostic biopsy, within 28 days of the first administration of trial treatment.

E09. Curative radiation within 3 months of the first dose of trial treatment. Radiation to more than 30% of the bone marrow or with a wide field of radiation should not be used within 2 weeks prior to the first administration of trial treatment.

E10. Prior therapy with any antibody/drug targeting T-cell coregulatory proteins (immune checkpoints) such as PD-1, PD-L1, cytotoxic T-lymphocyte antigen-4 (CTLA-4), LAG-3, or curative cancer vaccine.

E11. Subjects with a condition requiring systemic treatment with either corticosteroids (> 10 mg daily prednisone equivalents) or other immunosuppressive medications within 14 days of study drug administration. Inhaled or topical steroids, and adrenal replacement doses ≤ 10 mg daily prednisone equivalents are permitted in the absence of active autoimmune disease. A brief course of corticosteroids for the prophylaxis (e.g., contrast dye allergy) or treatment of non-autoimmune conditions (e.g., delayed-type hypersensitivity reaction caused by contact allergen) is permitted.

E12. Vaccination within 28 days of the first administration of trial treatment, including live attenuated vaccine.

E13. Has interstitial lung disease, or a history of pneumonitis that required oral or intravenous glucocorticoids to assist with management.

E14. History or current active autoimmune disease that might deteriorate when receiving an immunostimulatory agent, including but not limited to:

Myasthenia gravis (MG), Good syndromes, ISAACS syndromes, polymyositis, myocarditis, neuromuscular syndrome (myotonic dystrophy myositis, Eaton-Lambert syndrome), blood disorders (red cell aplasia, hypogammaglobulinemia, T-cell deficiency syndrome, erythrocytosis, pancytopenia, megakaryocytopenia, T-cell lymphocytosis, pernicious anemia), systemic lupus erythematosus, sarcoidosis, scleroderma, Crohn’s disease, inflammatory bowel disease, Wegener syndrome (granulomatosis with polyangitis, Grave’s disease, rheumatoid arthritis, hypophysitis, uveitis), autoimmune hepatitis, systemic sclerosis (for example scleroderma), Hashimoto thyroiditis (with the exception as stated below), hyperparathyroidism, stiff-person syndrome, Addison disease, panhypopituitarism, autoimmune vasculitis, autoimmune neuropathy (Guillain-Barre syndrome) etc.

Subjects with Type I diabetes, vitiligo, psoriasis, hypo- or hyperthyroid disease, Sjögren syndrome not requiring immunosuppressive treatment are eligible. Subjects requiring hormone replacement with corticosteroids are eligible if the steroids are administered only for the purpose of hormonal replacement and at doses ≤10 mg or equivalent prednisone per day. Administration of steroids for other conditions through a route known to result in a minimal systemic exposure (topical, intranasal, intra-ocular, or inhalation) are acceptable.

E15. Previous malignant disease other than the target malignancy to be investigated in this study except for adequately treated non-melanomatous cancers of the skin, in situ carcinoma of the prostate/cervical/breast cancer, NMIBC-Tis or other malignancy treated at least 5 years previously with surgery and/or curative radiotherapy, and there is no evidence of recurrence since that time.

E16. History of uncontrolled intercurrent illness including but not limited to:

- Active HBV or HCV infection.
  - If HBsAg and HCV antibody positive, HBV DNA and HCV RNA assay should be performed. Subjects maybe eligible if HBV DNA ≤ 500 UI/ml (or 2000 copies/ml) or HCV RNA negative.
- Known HIV infection or known history of acquired immune deficiency syndrome (AIDS);
- Active tuberculosis infection.
- Active infection within 4 weeks prior to the first dose of trial treatment that require the use of systemic antibiotics ≥ 7 days.

- Hypertension uncontrolled by standard therapies (not stabilized to 150/90 mmHg).
- Clinically significant (that is, active) cardiovascular disease: cerebral vascular accident/stroke (< 6 months prior to enrolment), myocardial infarction (< 6 months prior to enrolment), unstable angina pectoris, congestive heart failure (New York Heart Association Classification Class II-IV) or serious cardiac arrhythmia requiring medication (including corrected QT interval prolongation of > 470 msec calculated according to Fridericia and/or pacemaker or prior diagnosis of congenital long QT syndrome.

E17. Persisting toxicity related to prior therapy (including any prior investigational therapy) of CTCAE ≥ grade 2 (NCI-CTCAE v5.0) or related toxicity not recovery to baseline, with the exception of alopecia of any grade.

E18. Prior allo-HSCT or solid organ transplant.

E19. Known severe hypersensitivity reactions to antibody drug (≥ grade 3 NCI-CTCAE v5.0), any history of anaphylaxis, uncontrolled asthma (that is, 3 or more features of partially controlled asthma), or any history of severe drug hypersensitivity (for example immune mediated liver toxicity, immune mediated thrombocytopenia or anemia).

E20. Is pregnant or breastfeeding.

E21. Other medical conditions that at the discretion of investigator interfere with the requirements of the trial in terms of safety or efficacy evaluation, or treatment compliance. These include but are not limited to psychiatric or substance abuse disorder, moderate to large pleural fluid/cardiac effusion/ascites, or recurrent/refractory pleural fluid/cardiac effusion/ascites.

Total expected number of patients:

KN046 monotherapy cohort: Approximately 30 ~ 60 planned.

KN046 combination therapy cohort: Approximately 25 ~ 50 planned.

STUDY TREATMENT(s)

Investigational medicinal product: KN046.

Formulation: 40 mg/1.6 mL/vial; 300 mg/12 mL/vial

Route(s) of administration: intravenous infusion over at least 90 minutes (90~120 minutes)

Dose regimen: 3 mg/kg IV d1, 15 or 5 mg/kg IV d1, 15

Treatment period: every 28 days

Combination medicinal product: nab-paclitaxel.

Formulation: 100 mg/vial

Route(s) of administration: intravenous infusion over at 30 minutes (+15 minutes)

Dose regimen: 100 mg/m^2 IV d1, 8, 15, Q4W

Treatment period: every 28 days

ENDPOINT(S)

Primary endpoint:

- Objective response rate (ORR) and duration of response (DOR) per RECIST v1.1 by independent review committee (IRC).

Secondary endpoint(s):

- ORR and DOR per RECIST v1.1 by investigator.
- 6-months progression free survival rate (PFSR), 12-months PFSR, clinical benefit rate (CBR, defined as the proportion of subjects with best overall response of CR, PR, or SD ≥ 24 weeks), per RECIST v1.1 by IRC and investigator.
- 6-months overall survival rate (OS rate) and 12-months OS rate.
- Incidence and severity (as graded by CTCAE v5.0), seriousness and relationship to the trial treatments, abnormal findings on any laboratory test and physical examination.
- Status (positive or negative) and serum titers of anti-KN046 antibody and neutralizing capacity.
- Concentration-time profiles of KN046 and individual PK parameters of KN046 derived from population PK analysis, including but not limited to AUC_{ss}, $C_{max,ss}$, $C_{min,ss}$, CL and $T_{1/2}$.
- The correlation between biomarkers (PD-L1 expression, BRCA1/2 mutations, HRD, TMB) and clinical efficacy parameters (ORR, CBR, PFSR).
- The correlation between pharmacokinetic parameters ($AUCt_{au,ss}$, $C_{trough,ss}$, etc.) of KN046 and clinical efficacy parameters (ORR, CBR, PFSR, etc.).

Exploratory endpoints:

- The correlation between pharmacokinetic parameters ($AUCt_{au,ss}$, $C_{trough,ss}$, etc.) of KN046 and safety indicators.

ASSESSMENT SCHEDULE: see tables 1, 2, and 3

STATISTICAL CONSIDERATIONS

Sample size determination:

30 subjects and 25 subjects will be enrolled into KN046 monotherapy cohort and KN046 combination therapy cohort, respectively, based on the dose regimen. The sample size calculation is based on the Clopper-Pearson method to estimate the 95% confidence interval for ORR.

Sample size	ORR, %	ORR 95% CI
30（Mono）	5%	(0.82%, 17.2%)
	10%	(2.1%, 26.5%)
	15%	(5.6%, 30.7%)
	20%	(7.7%, 38.6%)
	25%	(12.3%, 42.3%)
	30%	(14.7%, 49.4%)
25（Combination）	40%	(21.1%, 61.3%)
	45%	(24.4%, 65.1%)
	50%	(31.3%, 72.2%)

	55%	(34.9%, 75.6%)
	60%	(38.7%, 78.9%)
	65%	(42.5%, 82.0%)
	70%	(50.6%, 87.9%)
	75%	(54.9%, 90.6%)

Analysis population:

- **Safety Set (SAS):** all subjects who received at least one (full or partial) dose of study treatment. Subjects will be classified according to the treatment prescribed in the protocol. Unless otherwise stated, SAS will be the default analysis set for all analyses.

- **Efficacy Analysis Set (EAS):** all subjects who received at least one (full or partial) dose of study treatment and had at least one post-baseline tumor imaging assessment. EAS will be used for the analysis of ORR and DOR.

- **Pharmacokinetic Analysis Set (PAS):** all subjects who receive at least one (full or partial) dose of study treatment and provide at least one post treatment KN046 concentration value above the lower limit of quantification (LLOQ) of the assay.

- **Immunogenicity Analysis Set (IAS):** all subjects who receive at least one (full or partial) dose of KN046 and provide at least one post treatment anti-KN046 antibody result.

Primary analysis:

- **ORR and DOR:** Based on EAS, ORR analysis is summarized by treatment cohort, and 95% confidence interval (CI) will be calculated using the Clopper Pearson method. DOR will also be summarized by treatment cohort using the Kaplan-Meier method.

Analysis of secondary endpoints:

- Safety analyses will be performed based on the SAS. Descriptive statistical analyses of safety endpoints will be performed by cohort. Safety analyses will be based on the incidence of AEs, TEAEs, irAEs, treatment-related AEs (TRAEs), and changes in vital signs, ECGs, weights, ECOG scores and laboratory values (hematology and serum chemistry). The treatment period is defined as the period from the first dose of study drugs to 90 days after the last dose of study drugs, or until 1 day before the initiation of a new anti-tumor therapy, whichever occurs first.

- Efficacy endpoints related to tumor assessment will be analyzed based on the EAS, and PFS and OS will be analyzed based on the SAS. The exact 95% CIs calculated by the Clopper Pearson method will be reported by cohort for individual proportions (CBR); for time-related events (DOR, PFS, OS), the parameters (including median and 95% CIs) calculated by the Kaplan-Meier method will be reported by cohort.

- The frequency of anti-KN046 antibodies (ADAs) and NADAs will be listed by cohort for immunogenicity, and descriptive statistical analysis will be performed for each cohort; for ADA-positive subjects, the titer of ADAs will be presented; and the frequency of low/medium/high-titer ADAs will also be presented for each cohort.

- PK analysis will be performed based on the measured KN046 plasma concentrations and actual blood sample collection time points, and individual PK parameters will be calculated, including AUCtau, ss, C_{max}, $C_{trough, ss}$, CL, V, and $T_{1/2}$, etc. Descriptive statistical analysis will be performed on PK parameters, including mini, max, median, arithmetic mean, geometric mean, and coefficient of variation (CV)%. The specific analyses and the corresponding methods will be detailed in a separate PK/pharmacodynamic analysis plan.

- Correlation between PK parameters and clinical efficacy endpoints will be based on Logistic regression analysis of AUC ($AUC_{tau,ss}$), $C_{trough,ss}$ and C_{max} against clinical efficacy endpoints (ORR, CBR, PFS rate, OS rate); PK parameters will be calculated by population PK modeling.

Flow charts

1.1 Overall study design

Figure 1 Study design of KN046 monotherapy

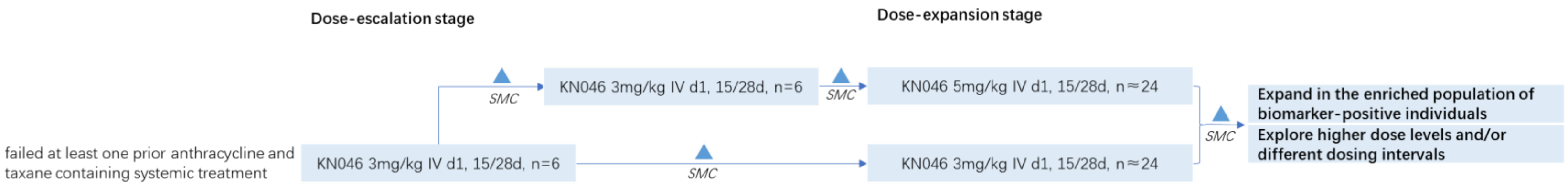

Figure 2 Study design of KN046 and nab- paclitaxel combination therapy

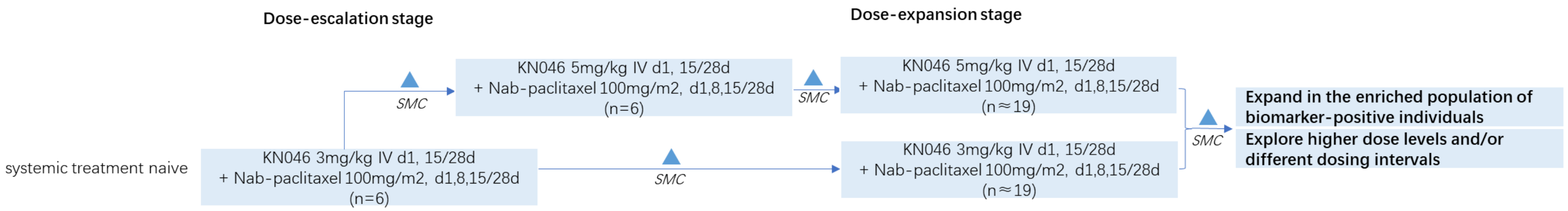

Assessment of schedule

Table 1 Assessment schedule of KN046 monotherapy

**Assessment**	**Screening**	**Treatment Period**									**End of Treatment (EOT)**	**30-day Safety Follow-up**	**90-day Safety Follow-up**	**Survival Follow-up**
Visit	Screening	Week 1	Week 2	Week 3	Week 4	Week 5	Week 6	Week 7	Week 8	Repeat Week 1-4 from Week 9 every 4 weeks	EOT	30-day FU	90-day FU	Every 12 weeks
Day (d)	(-28~-1)	1		1<br>±3		1<br>±3		1<br>±3		1<br>±3	(within 7 days after last dose) [1]	30 days after last dose of trial treatment (±3 d)	90 days after last dose of trial treatment (±7 d)	±14 d
**General procedure**														
Informed consent	X													
Biopsy consent (optional, not mandatory)[2]	X													
Inclusion / exclusion criteria	X													
Demographics	X													
Medical history	X													
Oncology history	X													
Prior and concomitant medications and procedures [2]	X	X									X	X	(X)	(X)
New anti-cancer therapy											X	X	X	X
Survival status														X
**Clinical examination**														
AE [4]	X	X									X	X	X	X
Full physical examination	X										X			
Symptom-directed examination		X		X		X		X		X		X	X	
Height	X													

Assessment	Screening	Treatment Period									End of Treatment (EOT)	30-day Safety Follow-up	90-day Safety Follow-up	Survival Follow-up
Visit	Screening	Week 1	Week 2	Week 3	Week 4	Week 5	Week 6	Week 7	Week 8	Repeat Week 1-4 from Week 9 every 4 weeks	EOT	30-day FU	90-day FU	Every 12 weeks
Day (d)	(-28~-1)	1		1 ±3		1 ±3		1 ±3		1 ±3	(within 7 days after last dose) [1]	30 days after last dose of trial treatment (±3 d)	90 days after last dose of trial treatment (±7 d)	±14 d
Body weight	X	X		X		X		X		X	X	X	X	
Vital signs [5]	X	X		X		X		X		X	X	X	X	
12-lead ECG	X										X			
ECOG performance status	X	X		X		X		X		X	X	X	X	
**Local laboratory test [6]**														
Hematology test (including reticulocyte) [7]	X [10]	(X)		X		X		X		X	X	(X) [11]		
Coagulation test	X [10]	Clinically indicated												
Serum chemistry [8]	X [10]	(X)		X		X		X		X	X	(X) [11]		
Urinalysis [9]	X [10]	Every 12 weeks; or as clinically indicated									X	(X) [11]		
T3, FT3, FT4 and TSH	X	Every 6 weeks; or as clinically indicated									X	(X) [11]		
ACTH	X	Every 6 weeks; or as clinically indicated									X	(X) [11]		
Troponin	X	Clinically indicated												
Serum β-HCG (if applicable)	(X) [8]										(X)			
Urine β-HCG (if applicable)		(Every 12 weeks)												
FSH (when need to confirm menopausal status)	(X)													
HBV, HCV, HIV [12]	X													
CRP	X													
**Central laboratory test**														
PK, ADA		Table 3												

**Assessment**	**Screening**	**Treatment Period**									**End of Treatment (EOT)**	**30-day Safety Follow-up**	**90-day Safety Follow-up**	**Survival Follow-up**
Visit	Screening	Week 1	Week 2	Week 3	Week 4	Week 5	Week 6	Week 7	Week 8	Repeat Week 1-4 from Week 9 every 4 weeks	EOT	30-day FU	90-day FU	Every 12 weeks
Day (d)	(-28~-1)	1		1 ±3		1 ±3		1 ±3		1 ±3	(within 7 days after last dose) [1]	30 days after last dose of trial treatment (±3 d)	90 days after last dose of trial treatment (±7 d)	±14 d
Biomarker (tumor tissue collection, optional) [13]	X													
Biomarker (blood, optional)[13]	X													
Tumor tissue biopsy before and after treatment [2]	(X) [2]		(X) [2]		(X) [2]									
**Tumor evaluation**														
Tumor imaging (chest/abdomen/pelvis) [14, 15]	X	Every 8 weeks within 12 months and every 12 weeks after 12 months (±7 days)									(X)	(X)	(X)	(X)
Tumor imaging (brain if applicable) [14, 15]	(X)	(clinically indicated)												
Bone scan (if applicable) [16]	(X)	(clinically indicated)												
**Investigational drug**														
KN046 administration [17]		X		X		X		X		d1,15/28d				

1. The EOT visit can be conducted on the day of the decision to discontinue KN046 treatment, and if it is conducted on the same day as the last pre-treatment evaluation, the same examination items do not need to be repeated.
2. Each subject will be asked for consent to participate in the non-mandatory biomarker assessment. Subjects who sign the consent (in either monotherapy or combination therapy) will be allocated to two groups. The first group of subjects will undergo fresh tumor biopsy at baseline and at week 2 after the first dose of KN046, while the second group of subjects will undergo fresh tumor biopsy at baseline and at week 4 after the first dose of KN046, for the analysis of changes in tumor-infiltrating lymphocyte (TIL) before and after treatment.
3. All medications (including herbal medications) taken and procedures performed within 28 days prior to first trial treatment should be recorded; radiation therapy other than the target indication of this study performed within 3 months prior to first trial treatment should be recorded;

4. All AEs will be collected from time of signature of inform consent through 30 days following the last dose of study drug or the date of subject initiates new anticancer therapy, whichever is earlier. All SAE and treatment related adverse event will be collected from the time of informed consent through 90 days following the last dose of study drug. However, if an investigator learns of any SAE, after 90-day safety follow-up period, and she/he considers there is a reasonable possibility that the event is related to the study drug, the investigator should report to sponsor. All AEs and SAEs should be proactively followed up for each patient, adverse events should be followed to resolution or stabilization at a level acceptable to the investigator.
5. Vital signs include temperature, respiration rate, pulse rate and blood pressure. In the D1 of 1st to 4th doses of KN046, they will be measured before the infusion (-60 min), 30 minutes (± 10 min) after the infusion, 15 minutes after the end of infusion, and 2 hours (+ 30 min) after the end of infusion. Vital signs measurement should be performed before PK sampling at respective time points.
6. Laboratory evaluation should be obtained before KN046 administration; Within 7 days before the first KN046 dose, the results should be obtained and used for inclusion/exclusion criteria assessment. The laboratory evaluation includes hematology test, coagulation test (PT/INR/aPTT), serum biochemistry, urinalysis, TSH/T3/free T3/free T4, ACTH, troponin, HBV/HCV/HIV, serum/urine β-HCG test. For detailed description, please see Section 7.3.3.
7. Hematology test includes absolute lymphocyte count, absolute neutrophil count, hematocrit, hemoglobin, platelet count, red blood cells, white blood cells and differential count, red blood cell morphology, reticulocytes, mean corpuscular hemoglobin, mean corpuscular volume, mean corpuscular hemoglobin concentration.
8. Serum chemistry test includes albumin, alkaline phosphatase, alanine aminotransferase, amylase, aspartate aminotransferase, gamma-glutamyl transferase, blood urea nitrogen/total urea, calcium, chloride, cholesterol, creatinine, glucose, lactate dehydrogenase, lipase, phosphorus/phosphates, magnesium, potassium, sodium, troponin, total bilirubin, total protein, uric acid.
9. Urinalysis includes bilirubin, blood, glucose, ketones, pH, protein, specific gravity, and color and appearance. If urinalysis is positive for protein, segments and 24-hour urine protein exam should be performed.
10. Examination or test results should be obtained within 7 days before each KN046 administration.
11. Items with clinically significant abnormal findings at EOT visit should be repeated at 30-day safety follow-up.
12. HBV, HCV, and HIV tests include HBsAb, HCV RNA and anti-HIV1/2. If HBsAb is positive, HBsAg, HBeAb, HBeAg, HBcAb and HBV DNA should be measured to exclude active HBV infection as clinically indicated.
13. A tumor biopsy should be collected at screening unless tissue (blocks or slides) from an archival specimen (biopsy or surgery) is available and was obtained no more than 2 years from non-radiation area prior to screening, which is used for BRCA1/2 mutations, HRD mutations, TMB, PD-L1 expression, and TIL analysis. Endoscopic biopsies, core needle biopsies, excisional biopsies, punch biopsies, and surgical specimens are suitable. Fine needle aspiration biopsies are not suitable. Biopsies are only to be obtained from safely accessible tumor tissue/sites. Priority 1: tumor containing FFPE tissue block; Priority 2: if the tumor containing FFPE tissue block cannot be provided in total, sections from this block should be provided that are freshly cut (within 1 week), 4 μm thick, and mounted on SuperFrost Plus microscope slides. Subjects should be encouraged to provide as many slides as possible, and no less than 10 slides should be provided. If not, the investigators and the sponsor's medical monitor need to discuss whether to enroll the subject. Each subject is required to provide a serum sample during the screening period for BRCA and HRD mutation analysis.
14. Tumor assessment will be performed preferably using CT assessments of chest, abdomen and pelvis as appropriate with IV contrast. Subjects who are intolerant of IV contrast agents may have CT scans performed with oral contrast and the reason for not using IV contrast will be documented in source documents. If magnetic resonance imaging (MRI) is used instead, CT of chest is mandatory. MRI may be used to evaluate sites of disease that cannot be adequately imaged using CT (in any case where an MRI is desirable, it must be the imaging technique used to assess disease at baseline and at all subsequent response evaluations for that site. Screening tumor assessment should be performed within 21 days of first trial treatment in order to document baseline status of tumor disease using RECIST 1.1 target and non-target lesions. Brain

CT/MRI scan (either, with contrast preferred) is required at screening if not performed within the previous 42 days prior to first trial treatment. During treatment period, tumor assessment should be performed every 8 weeks within 12 months and every 12 weeks after 12 months, and the tumor assessment visit time window is ±7 days. CT or MRI scan (if MRI is used, CT of chest is mandatory) should always be used the same to screening period. Tumor assessment will continue until disease progression, start of new anti-cancer therapy, or withdrawal of informed consent, die, end of study, whichever comes first. During treatment period, brain CT/MRI scan should be done if clinically indicated by development of new specific symptoms. If a tumor response is documented during the study, confirmation of the response should be performed according to RECIST 1.1, preferably at the regularly scheduled 8-week assessment interval, but no sooner than 4 weeks and no later than 8 weeks after the initial documentation of CR or PR. Confirmation of PR/CR can be done at an assessment later than the next assessment after the initial documentation of PR/CR. If disease progression per RECIST 1.1 is documented, confirmation of PD is required, preferably at an 8-week assessment interval (but no sooner than 4 weeks and no later than 8 weeks). Tumor assessment will continue until confirmed disease progression per RECIST 1.1, start of new anti-cancer therapy, or withdrawal of informed consent, whichever comes first.

15. If treatment discontinuation is due to reasons other than RECIST 1.1 defined progressive disease (such as intolerable toxicity, clinical deterioration), tumor assessment should continue until RECIST 1.1 defined progressive disease, start of new anti-cancer therapy, or withdrawal of informed consent, whichever comes first (Section 7.3.2.3). If disease progression is confirmed according to RECIST 1.1 but meets the criteria for continuing treatment after progression (section 6.1.3), and the investigator judges that there may be clinical benefit from continuing KN046 treatment, it is allowed to refer to the iRECIST [19] to follow-up for tumor assessment .
16. A bone scan should be performed if not be done within 3 months prior to first trial treatment. During treatment period, Bone scan should be done as clinically indicated.
17. Each KN046 will be administered intravenously over at least 90 minutes (90~120 minutes). After the completion of KN046 infusion, subject should be observed at site for at least 2 hours.

Assessment of schedule

Table 2 Assessment schedule of KN046 and nab- paclitaxel combination therapy

**Assessment**	**Screening**	**Treatment Period**									**End of Treatment (EOT)**	**30-day Safety Follow-up**	**90-day Safety Follow-up**	**Survival Follow-up**
Visit	Screening	Week 1	Week 2	Week 3	Week 4	Week 5	Week 6	Week 7	Week 8	Repeat Week 1-4 from Week 9 every 4 weeks	EOT	30-day FU	90-day FU	Every 12 weeks
Day (d)	(-28~-1)	1		1<br>±3		1<br>±3		1<br>±3		1<br>±3	(within 7 days after last dose) [1]	30 days after last dose of trial treatment (±3 d)	90 days after last dose of trial treatment (±7 d)	±14 d
**General procedure**														
Informed consent	X													
Biopsy consent (optional, not mandatory)[2]	X													
Inclusion / exclusion criteria	X													
Demographics	X													
Medical history	X													
Oncology history	X													
Prior and concomitant medications and procedures [2]	X	X									X	X	(X)	(X)
New anti-cancer therapy											X	X	X	X
Survival status														X
**Clinical examination**														
AE [4]	X	X									X	X	X	X
Full physical examination	X										X			
Symptom-directed examination		X		X		X		X		X		X	X	
Height	X													
Body weight	X	X		X		X		X		X	X	X	X	

Assessment	Screening	Treatment Period									End of Treatment (EOT)	30-day Safety Follow-up	90-day Safety Follow-up	Survival Follow-up
Visit	Screening	Week 1	Week 2	Week 3	Week 4	Week 5	Week 6	Week 7	Week 8	Repeat Week 1-4 from Week 9 every 4 weeks	EOT	30-day FU	90-day FU	Every 12 weeks
Day (d)	(-28~-1)	1		1 ±3		1 ±3		1 ±3		1 ±3	(within 7 days after last dose) [1]	30 days after last dose of trial treatment (±3 d)	90 days after last dose of trial treatment (±7 d)	±14 d
Vital signs [5]	X	X		X		X		X		X	X	X	X	
12-lead ECG	X										X			
ECOG performance status	X	X		X		X		X		X	X	X	X	
**Local laboratory test [6]**														
Hematology test (including reticulocyte) [7]	X [10]	(X)		X		X		X		X	X	(X) [11]		
Coagulation test	X [10]	Clinically indicated												
Serum chemistry [8]	X [10]	(X)		X		X		X		X	X	(X) [11]		
Urinalysis [9]	X [10]	Every 12 weeks; or as clinically indicated									X	(X) [11]		
T3, FT3, FT4 and TSH	X	Every 6 weeks; or as clinically indicated									X	(X) [11]		
ACTH	X	Every 6 weeks; or as clinically indicated									X	(X) [11]		
Troponin	X	Clinically indicated												
Serum β-HCG (if applicable)	(X) [8]										(X)			
Urine β-HCG (if applicable)		(Every 12 weeks)												
FSH (when need to confirm menopausal status)	(X)													
HBV, HCV, HIV [12]	X													
CRP	X													
**Central laboratory test**														
PK, ADA		Table 3												
Biomarker (tumor tissue	X													

Assessment	Screening	Treatment Period									End of Treatment (EOT)	30-day Safety Follow-up	90-day Safety Follow-up	Survival Follow-up
Visit	Screening	Week 1	Week 2	Week 3	Week 4	Week 5	Week 6	Week 7	Week 8	Repeat Week 1-4 from Week 9 every 4 weeks	EOT	30-day FU	90-day FU	Every 12 weeks
Day (d)	(-28~-1)	1		1<br>±3		1<br>±3		1<br>±3		1<br>±3	(within 7 days after last dose) [1]	30 days after last dose of trial treatment (±3 d)	90 days after last dose of trial treatment (±7 d)	±14 d
collection, optional) [13]														
Biomarker (blood, optional)[13]	X													
Tumor tissue biopsy before and after treatment [2]	(X) [2]		(X) [2]		(X) [2]									
**Tumor evaluation**														
Tumor imaging (chest/abdomen/pelvis) [14, 15]	X	Every 8 weeks within 12 months and every 12 weeks after 12 months (±7 days)									(X)	(X)	(X)	(X)
Tumor imaging (brain if applicable) [14, 15]	(X)	(clinically indicated)												
Bone scan (if applicable) [16]	(X)	(clinically indicated)												
**Investigational drug**														
KN046 administration [17]		X		X		X		X		d1,15/28d				
Nab-paclitaxel administration [18]														

1. The EOT visit can be conducted on the day of the decision to discontinue KN046 treatment, and if it is conducted on the same day as the last pre-treatment evaluation, the same examination items do not need to be repeated.
2. Each subject will be asked for consent to participate in the non-mandatory biomarker assessment. Subjects who sign the consent (in either monotherapy or combination therapy) will be allocated to two groups. The first group of subjects will undergo fresh tumor biopsy at baseline and at week 2 after the first dose of KN046, while the second group of subjects will undergo fresh tumor biopsy at baseline and at week 4 after the first dose of KN046, for the analysis of changes in tumor-infiltrating lymphocyte (TIL) before and after treatment.
3. All medications (including herbal medications) taken and procedures performed within 28 days prior to first trial treatment should be recorded; radiation therapy other than the target indication of this study performed within 3 months prior to first trial treatment should be recorded;

4. All AEs will be collected from time of signature of inform consent through 30 days following the last dose of study drug or the date of subject initiates new anticancer therapy, whichever is earlier. All SAE and treatment related adverse event will be collected from the time of informed consent through 90 days following the last dose of study drug. However, if an investigator learns of any SAE, after 90-day safety follow-up period, and she/he considers there is a reasonable possibility that the event is related to the study drug, the investigator should report to sponsor. All AEs and SAEs should be proactively followed up for each patient, adverse events should be followed to resolution or stabilization at a level acceptable to the investigator.
5. Vital signs include temperature, respiration rate, pulse rate and blood pressure. In the D1 of 1st to 4th doses of KN046, they will be measured before the infusion (-60 min), 30 minutes (± 10 min) after the infusion, 15 minutes after the end of infusion, and 2 hours (+ 30 min) after the end of infusion. Vital signs measurement should be performed before PK sampling at respective time points.
6. Laboratory evaluation should be obtained before KN046 administration; Within 7 days before the first KN046 dose, the results should be obtained and used for inclusion/exclusion criteria assessment. The laboratory evaluation includes hematology test, coagulation test (PT/INR/aPTT), serum biochemistry, urinalysis, TSH/T3/free T3/free T4, ACTH, troponin, HBV/HCV/HIV, serum/urine β-HCG test. For detailed description, please see Section 7.3.3.
7. Hematology test includes absolute lymphocyte count, absolute neutrophil count, hematocrit, hemoglobin, platelet count, red blood cells, white blood cells and differential count, red blood cell morphology, reticulocytes, mean corpuscular hemoglobin, mean corpuscular volume, mean corpuscular hemoglobin concentration.
8. Serum chemistry test includes albumin, alkaline phosphatase, alanine aminotransferase, amylase, aspartate aminotransferase, gamma-glutamyl transferase, blood urea nitrogen/total urea, calcium, chloride, cholesterol, creatinine, glucose, lactate dehydrogenase, lipase, phosphorus/phosphates, magnesium, potassium, sodium, troponin, total bilirubin, total protein, uric acid.
9. Urinalysis includes bilirubin, blood, glucose, ketones, pH, protein, specific gravity, and color and appearance. If urinalysis is positive for protein, segments and 24-hour urine protein exam should be performed.
10. Examination or test results should be obtained within 7 days before each KN046 administration.
11. Items with clinically significant abnormal findings at EOT visit should be repeated at 30-day safety follow-up.
12. HBV, HCV, and HIV tests include HBsAb, HCV RNA and anti-HIV1/2. If HBsAb is positive, HBsAg, HBeAb, HBeAg, HBcAb and HBV DNA should be measured to exclude active HBV infection as clinically indicated.
13. A tumor biopsy should be collected at screening unless tissue (blocks or slides) from an archival specimen (biopsy or surgery) is available and was obtained no more than 2 years from non-radiation area prior to screening, which is used for BRCA1/2 mutations, HRD mutations, TMB, PD-L1 expression, and TIL analysis. Endoscopic biopsies, core needle biopsies, excisional biopsies, punch biopsies, and surgical specimens are suitable. Fine needle aspiration biopsies are not suitable. Biopsies are only to be obtained from safely accessible tumor tissue/sites. Priority 1: tumor containing FFPE tissue block; Priority 2: if the tumor containing FFPE tissue block cannot be provided in total, sections from this block should be provided that are freshly cut (within 1 week), 4 μm thick, and mounted on SuperFrost Plus microscope slides. Subjects should be encouraged to provide as many slides as possible, and no less than 10 slides should be provided. If not, the investigators and the sponsor's medical monitor need to discuss whether to enroll the subject. Each subject is required to provide a serum sample during the screening period for BRCA and HRD mutation analysis.
14. Tumor assessment will be performed preferably using CT assessments of chest, abdomen and pelvis as appropriate with IV contrast. Subjects who are intolerant of IV contrast agents may have CT scans performed with oral contrast and the reason for not using IV contrast will be documented in source documents. If magnetic resonance imaging (MRI) is used instead, CT of chest is mandatory. MRI may be used to evaluate sites of disease that cannot be adequately imaged using CT (in any case where an MRI is desirable, it must be the imaging technique used to assess disease at baseline and at all subsequent response evaluations for that site. Screening tumor assessment should be performed within 21 days of first trial treatment in order to document baseline status of tumor disease using RECIST 1.1 target and non-target lesions. Brain

CT/MRI scan (either, with contrast preferred) is required at screening if not performed within the previous 42 days prior to first trial treatment. During treatment period, tumor assessment should be performed every 8 weeks within 12 months and every 12 weeks after 12 months, and the tumor assessment visit time window is $\pm$7 days. CT or MRI scan (if MRI is used, CT of chest is mandatory) should always be used the same to screening period. Tumor assessment will continue until disease progression, start of new anti-cancer therapy, or withdrawal of informed consent, die, end of study, whichever comes first. During treatment period, brain CT/MRI scan should be done if clinically indicated by development of new specific symptoms. If a tumor response is documented during the study, confirmation of the response should be performed according to RECIST 1.1, preferably at the regularly scheduled 8-week assessment interval, but no sooner than 4 weeks and no later than 8 weeks after the initial documentation of CR or PR. Confirmation of PR/CR can be done at an assessment later than the next assessment after the initial documentation of PR/CR. If disease progression per RECIST 1.1 is documented, confirmation of PD is required, preferably at an 8-week assessment interval (but no sooner than 4 weeks and no later than 8 weeks). Tumor assessment will continue until confirmed disease progression per RECIST 1.1, start of new anti-cancer therapy, or withdrawal of informed consent, whichever comes first.

15. If treatment discontinuation is due to reasons other than RECIST 1.1 defined progressive disease (such as intolerable toxicity, clinical deterioration), tumor assessment should continue until RECIST 1.1 defined progressive disease, start of new anti-cancer therapy, or withdrawal of informed consent, whichever comes first (Section 7.3.2.3). If disease progression is confirmed according to RECIST 1.1 but meets the criteria for continuing treatment after progression (section 6.1.3), and the investigator judges that there may be clinical benefit from continuing KN046 treatment, it is allowed to refer to the iRECIST [19] to follow-up for tumor assessment.
16. A bone scan should be performed if not be done within 3 months prior to first trial treatment. During treatment period, Bone scan should be done as clinically indicated.
17. Each KN046 will be administered intravenously over at least 90 minutes (90~120 minutes). After the completion of KN046 infusion, subject should be observed at site for at least 2 hours.
18. When KN046 and nab-paclitaxel are dosed on the same day, nab-paclitaxel should be dosed at least 2 hours after the completion of KN046 infusion.

Table 3 Assessment schedule (PK, ADA)

Treatment cycle (1 cycle = 28 days)	Days	Time	PK sample	ADA sample
1	1	0 h (KN046 pre-dose) (-60 min)	3.5 mL	3.5 mL
1	1	KN046 end of infusion (+30 min)	3.5 mL	
1	15	0 h (KN046 pre-dose) (-60 min)	3.5 mL	3.5 mL
2	1	0 h (KN046 pre-dose) (-60 min)	3.5 mL	3.5 mL
2	1	KN046 end of infusion (+30 min)	3.5 mL	
2	15	0 h (KN046 pre-dose) (-60 min)	3.5 mL	3.5 mL
3	1	0 h (KN046 pre-dose) (-60 min)	3.5 mL	3.5 mL
4	1	KN046 end of infusion (+30 min)	3.5 mL	3.5 mL
6	1	0 h (KN046 pre-dose) (-60 min)	3.5 mL	3.5 mL
8	1	0 h (KN046 pre-dose) (-60 min)	3.5 mL	3.5 mL
11	1	0 h (KN046 pre-dose) (-60 min)	3.5 mL	3.5 mL
14	1	0 h (KN046 pre-dose) (-60 min)	3.5 mL	3.5 mL
17	1	0 h (KN046 pre-dose) (-60 min)	3.5 mL	3.5 mL
20	1	0 h (KN046 pre-dose) (-60 min)	3.5 mL	3.5 mL
23	1	0 h (KN046 pre-dose) (-60 min)	3.5 mL	3.5 mL
30-day safety follow up			3.5 mL	3.5 mL
90-day safety follow up			3.5 mL	3.5 mL
Total			***59.5 mL***	***52.5 mL***

Table of contents

Clinical trial synopsis 4

Flow charts 11

Overall study design 11

Assessment of schedule 13

List of abbreviations 31

1 Background information 35

1.1 Immune checkpoint inhibitor 35

1.1.1 PD-1/PD-L1 immune checkpoint blockers 35

1.1.2 CTLA-4 immune checkpoint blockers 36

1.1.3 PD-1/PD-L1 in combination with ctla-4 checkpoint inhibitors 36

1.2 KN046 37

1.2.1 Preclinical PK study 37

1.2.2 Preclinical toxicology studies 37

1.2.3 Clinical studies 38

2 Rationale for the current clinical trial 46

2.1 Rationale for dose selection 46

2.2 Rationale for clinical endpoint 46

2.3 Rationale for indication selection 46

2.4 Rational for chemotherapy selection 47

2.5 Overall benefit and risk assessment 48

3 Trial objectives and endpoints 49

3.1 Trial objectives 49

3.1.1 Primary objective 49

3.1.2 Secondary objectives 49
3.1.3 Exploratory objective 49
3.2 Trial endpoints 49
3.2.1 Primary endpoint 49
3.2.2 Secondary endpoints 49
3.2.3 Exploratory endpoints 50
4 Study Design 51
4.1 Overall study design and plan 51
4.2 Definition of end of study 53
4.3 Premature termination of the study 53
4.4 Interim analysis 54
4.5 Scientific Monitoring Committee (SMC) 54
5 Selection of trial population 55
5.1 Inclusion criteria 55
5.2 Exclusion criteria 56
5.3 Criteria for subject withdrawal 59
5.3.1 Discontinuation of Study Drugs 59
5.3.2 Withdrawal from the study 59
6 Investigational medicinal product and trial treatment 61
6.1 Study drug (KN046) 61
6.1.1 Dosage form and strength 61
6.1.2 Drug preparation 61
6.1.3 Dose and method of administration of KN046 61
6.1.4 Dose modifications 62
6.1.5 Treatment Assignment 64

6.1.6 Drug Packaging and Labeling 64
6.1.7 Storage and handling requirements 65
6.1.8 Drug quantity management 65
6.1.9 Assessment of investigational medicinal product compliance 65
6.1.10 Occupational Safety 65
6.2 Study drug (Nab-paclitaxel) 65
6.2.1 Dose and method of administration of nab-paclitaxel 65
6.2.2 Dose modification of nab-paclitaxel 66
6.3 Concurrent medications and therapies 67
6.3.1 Permitted medications and procedures 67
6.3.2 Prohibited medications and procedures 67
6.3.3 Special precautions 68
7 Procedures and assessments 72
7.1 Schedule of visits plan 72
7.1.1 Screening and baseline period 72
7.1.2 Treatment period 73
7.2 End of treatment 75
7.2.1 Safety follow up 77
7.2.2 Long term follow up 78
7.3 Study assessments 79
7.3.1 Demographic and other baseline characteristics 79
7.3.2 Efficacy assessments 80
7.3.3 Safety assessments 83
7.3.4 Pharmacokinetics assessments 88
7.3.5 Immunogenicity assessment 88

7.3.6 Biomarker assessments....88
8 Adverse event assessment and record 90
8.1 Adverse event definition 90
8.1.1 Adverse event....90
8.1.2 Serious adverse event 92
8.1.3 AE of interest....93
8.2 Methods of recording and assessing adverse events 93
8.3 Definition of the adverse event reporting period....94
8.4 Procedure for reporting serious adverse events....94
8.5 Monitoring of subjects with adverse events 94
8.6 pregnancy and in utero drug exposure....94
9 Statistics....96
9.1 Statistical methods....96
9.2 Sample size....96
9.3 Analysis set....96
9.4 Demographic and Other Baseline Characteristics....97
9.5 Primary endpoint analysis 97
9.6 Secondary endpoint analysis 97
9.6.1 Safety endpoints 97
9.6.2 Efficacy endpoints....99
9.6.3 Pharmacokinetic Endpoints....99
9.6.4 Correlation between biomarkers and clinical response endpoints 99
9.6.5 Immunogenicity endpoints....100
9.7 Other endpoints 100
10 Data collection and management....101

10.1 Data confidentiality 101
10.2 Site monitoring 101
10.3 Data collection 101
10.4 Database management and quality control 102
11 Ethical and regulatory aspects 103
11.1 Responsibilities of the investigator 103
11.2 Independent ethnic committee or institutional review board 103
11.3 Health authorities 103
11.4 Informed consent 104
11.5 Subject identification and privacy 104
11.6 Clinical trial insurance and compensation 105
12 Trial management 106
12.1 Case report form handling 106
12.2 Source data and subject files 106
12.3 Investigator site file and archiving 107
12.4 Monitoring, quality assurance and inspection by health authorities 107
12.5 Changes to the clinical trial protocol 107
12.6 Clinical trial report and publication policy 107
13 Reference 109
14 Appendices 111
Appendix 1 RECIST 1.1 111
Appendix 2 Guidelines on the management of immune related adverse event 119
Appendix 3 Guidance on contraception 130
Appendix 4 Eastern Cooperative Oncology Group Performance Status 131
Appendix 5 Cockcroft-Gault Formula 132

LIST OF TABLES

Table 1 Assessment schedule of KN046 monotherapy 13
Table 2 Assessment schedule of KN046 and nab- paclitaxel combination therapy 18
Table 3 Assessment schedule (PK, ADA) 23
Table 4 Subject disposition for Trial KN046-AUS-001 (Safety Population) 40
Table 5 Safety summary for Trial KN046-CHN-001 (Safety Population) 41
Table 6 Treatment related TEAEs (TRAEs) for Trial KN046-AUS-001 (Safety Population) 42
Table 7 Immune-related AEs for Trial KN046-AUS-001 (Safety Population) 43
Table 8 Efficacy Summary for Trial KN046-AUS-001 (Efficacy Population) 44
Table 9 Summary of Pharmacokinetic Parameters on Cycle 1 Day 1 (KN046-AUS-001) 45
Table 10 Guideline on KN046 dose modification 63
Table 11 Guideline on nab-paclitaxel dose reduction 66
Table 12 Guideline of nab-paclitaxel modification on hematologic toxicities 66
Table 13 Guideline of nab-paclitaxel modification on hematologic toxicities 67
Table 14 Guideline on management of IRR and hypersensitivity reactions 69
Table 15 Laboratory Test Items 87
Table 16 Sample size calculation 96

LIST OF FIGURES

Figure 1 Study design of KN046 monotherapy 11
Figure 2 Study design of KN046 and nab- paclitaxel combination therapy 12
Figure 3 Evaluation and initial treatment of TLS 71

LIST OF ABBREVIATIONS

ACTH	adrenocorticotropic hormone
ADA	anti-drug antibody
ADCC	antibody-dependent cell-mediated cytotoxicity
ADL	activities of daily living
ADR	adverse drug reaction
AE	adverse event
AESI	adverse event of special interest
AIDS	acquired immune deficiency syndrome
AJCC	American Joint Committee on Cancer
ALT	alanine aminotransferase
ANA	antinuclear antibody
ANC	absolute neutrophil count
ANOVA	analysis of variance
aPTT	activated partial thromboplastin time
ARDS	acute respiratory distress syndrome
AST	aspartate aminotransferase
AUC	area under the curve
BAL	bronchoalveolar lavage
BED	biologically effective dose
BIRC	Blinded Independent Review Committee
BNP	brain natriuretic peptide
BOR	best overall response
CBR	clinical benefit rate
CDC	complement-dependent cytoxicity
CHO	Chinese hamster ovary
CI	confidence interval
CK	creatine kinase
C_{avg}	average concentration
C_{max}	maximum concentration
C_{min}	minimum concentration
CNS	central nervous system
CPK	creatine phosphokinase
CR	complete response
CRA	Clinical Research Associate
CRO	Contract Research Organization

CRP	C reactive protein
CT	computed tomography
CTCAE	Common Terminology Criteria for Adverse Events
CTLs	cytotoxic T lymphocytes
CTLA	cytotoxic T-lymphocyte-associated antigen
CTP	clinical trial protocol
DCR	disease control rate
DLT	dose limiting toxicity
DOR	duration of response
EAS	efficacy analysis set
ECG:	electrocardiogram
ECOG	Eastern Cooperative Oncology Group
eCRF	electronic case report form
EDC	electronic data capture
ENA	extractable nuclear antigens
EOT	end-of-treatment
ESMO	European Society for Medical Oncology
FAS	full analysis set
FFPE	formalin fixation and paraffin embedding
FNCLCC	Fédération Nationale des Centres de Lutte Contre le Cancer
FSH	follicle-stimulating hormone
FU	follow-up
GCP	Good Clinical Practice
GEP	gene expression profile
HAV	hepatitis A
HBsAb	hepatitis B surface antibody
HBsAg	hepatitis B surface antigen
HBV	hepatitis B
HCG	human chorionic gonadotropin
HCV	hepatitis C
HIV	human immunodeficiency virus
HSCT	hematopoietic stem cell transplantation
IAS	immunogenicity analysis set
ICF	informed consent form
ICH	International Conference on Harmonisation
IEC:	independent ethics committee
IHC	immunohistochemistry

IL	interleukin
IMP:	investigational medicinal product
INR	international normalized ratio
irAE	immune-related adverse event
IRB:	institutional review board
IRC	Independent Review Committee
IRR	infusion-related adverse event
IV	intravenous
LAG-3	lymphocyte-activiation gene 3
LLOQ	lower limit of quantification
mAbs	monoclonal antibodies
MG	myasthenia gravis
MOA	mechanism of action
MRI	magnetic resonance imaging
MTD	maximum tolerated dose
MuSK	muscle-specific kinase
NCI	National Cancer Institute
NE	not evaluable
NIMBC	non-muscle invasive bladder cancer
NSAIDs	non-steroidal anti-inflammatory drugs
ORR	overall response rate
OS	overall survival
PAS	pharmacokinetic analysis set
PD	pharmacodynamic
PD	progressive disease
PD-1	programmed cell death protein 1
PD-L1	programmed death-ligand 1
PK	pharmacokinetic
PFS	progression-free survival
PPS	per-protocol set
PR	partial response
PT	prothrombin time
Q2W	every 2 weeks
RECIST	Response Evaluation Criteria In Solid Tumors
RNA	ribonucleic acid
RP2D	recommended phase 2 dose
SAE:	serious adverse event

SAP	Statistical Analysis Plan
SD	stable disease
SD	standard deviation
SLD	sum of longitudinal diameter
SMC	Safety Monitoring Committee
SS	safety set
T3	triodothyronine
T4	thyroxine
TdT	terminal deoxynucleotidyl transferase
TEAE	treatment ermergent adverse event
TIM-3	T cell immunoglobulin and mucin domain-3
TIL	Tumor-Infiltrating Lymphocytes
TLS	tumor lysis syndrome
TMB	tumor mutational burden
TNBC	triple negative breast cancer
TRAE	treatment-related TEAE
TSH	thyroid stimulating hormone
TTP	time to progression
TTR	time to response
ULN	upper limit of normal
UNK	unknown
WOCBP	Women of Childbearing Potential

1 BACKGROUND INFORMATION

1.1 Immune checkpoint inhibitor

In 2013, cancer immunotherapy was ranked as No.1 of the top 10 scientific breakthrough events by scientific journals [1]. Immune checkpoints are molecules that positively or negatively regulate signals in the immune system. Positive regulator molecules are known as stimulatory checkpoint molecules (e.g., CD27, CD40, OX40, GITR, CD137, CD28, ICOS), and negative regulator molecules are known as inhibitory checkpoint molecules (e.g., CTLA-4, PD-1, LAG-3, TIM-3, IDO). Many cancer cells can escape killing by the immune system by inhibition of T-cell signaling [2].

At present, anti-PD-1/PD-L1 and anti-CTLA-4/CD80/CD86 antibodies have been identified as effective immune checkpoint blockers that can prolong the survival of multiple tumor types including melanoma, renal cell carcinoma, non-small cell lung cancer (NSCLC), bladder cancer, gastric cancer, etc.

1.1.1 PD-1/PD-L1 immune checkpoint blockers

Under normal physiological conditions, PD-1, as an immune checkpoint, can bind to its two ligands, PD-L1 and PD-L2, and can attenuate the immune system by suppressing T cells and up-regulating Tregs, thereby reducing autoimmunity and promoting self-tolerance. The PD-1/PD-L1 interaction is more important than PD-1/PD-L2. Both PD-1 and PD-L1 are membrane proteins located on the surface of different immune cells, and they interact (bind) to each other to form the PD-1/PD-L1 pathway. Studies have shown that tumor cells can use the PD-1/PD-L1 pathway to escape T-cell anti-tumor immunity. Currently, five PD-1/PD-L1 blockers have been approved by FDA, EMA, and PMDA: (1) Nivolumab is an anti-PD-1 agent developed by BMS and its approved indications include monotherapy or in combination with ipilumab for unresectable or metastatic melanoma, postoperative adjuvant therapy for melanoma, 2L NSCLC, 2L renal cell carcinoma, in combination with ipilimumab for 1L intermediate- and high-risk renal cell carcinoma, classical Hodgkin's tumor that has failed third- or more lines of therapies, recurrent or metastatic head and neck cancer that has failed platinum-based therapy, 2L therapy for recurrent or metastatic urothelial cancer, MSI-H metastatic colorectal cancer that has failed oxaliplatin/fluorouracil/irinotecan therapy, and hepatocellular carcinoma that has failed sorafenib therapy; (2) Pembrolizumab is another anti-PD-1 agent developed by MSD, and its approved indications include unresectable or metastatic melanoma, 1L therapy for NSCLC with PD-L1 ≥ 50%, 2L therapy for NSCLC with PD-L1 ≥ 1%, 1L therapy in combination with pemetrexed/carboplatin for NSCLC, recurrent or metastatic head and neck cancer that has failed platinum-based therapy, classical Hodgkin's tumor that has failed third- or more lines of therapies, primary mediastinal large B-cell lymphoma that has failed second or more lines of therapies, 1L or 2L therapy for urothelial cancer, MSI-H metastatic colorectal cancer or other MSI-H solid tumors without standard therapy, gastric cancer or gastroesophageal junction tumors with PD-L1 ≥ 1% that has failed second- or more lines of therapy, cervical cancer with PD-L1 ≥ 1% of that has failed chemotherapy; (3) Atezolizumab is an anti-PD-L1 agent developed by Genentech (Roche), and its approved indications include urethral epithelial cell carcinoma that has failed or intolerance to cisplatin, 2L therapy for NSCLC; (4) Durvalumab is an

anti-PD-L1 agent developed by AstraZeneca, and its approved indications include recurrent, locally advanced or metastatic urothelial cancer that has failed platinum-based chemotherapy, and Stage III NSCLC that has not progressed after concurrent chemoradiotherapy; and (5) Avelumab is an anti-PD-L1 agent developed by Merck/Pfizer, and its approved indications include Merkel cell carcinoma, recurrent, locally advanced or metastatic urothelial cell carcinoma that has failed platinum-based chemotherapy.

Although PD-1/PD-L1 has shown clinical benefit in multiple tumor types, objective response rates are still limited. Therefore, currently approved or unapproved PD-1/PD-L1 checkpoint blockers are undergoing hundreds of clinical studies, and further PD-1/PD-L1-based combination therapies or novel biomarkers are being developed to expand the beneficiary population.

1.1.2 CTLA-4 immune checkpoint blockers

CTLA-4, a homolog of the T cell costimulatory receptor CD28, also binds to CD80 and CD86 with higher affinity than CD28. Naive T cells (CD4+ FoxP3− and CD8+) are induced and activated to express CTLA-4. Because CTLA-4 is a direct transcriptional target of Foxp3, it is rapidly expressed without physiological requirements. Anti-CTLA-4 antibody have effects on both effector T cells and Tregs and can increase the ratio of CD4+ and CD8+ effector T cells to FoxP3+ Tregs in tumor infiltration. Where CD8+ T cells are essential for the anti-tumor effect of CTLA-4 antibodies, but CD4+ T cells are not. Anti-CTLA-4 antibodies can directly kill tumor cells by CD8+ T cells, and can also deplete Tregs cells in tumors by Fc-mediated ADCC. Two anti-CTLA-4 antibodies (ipilimumab and tremelimumab) have been well studied in clinical studies (MDX010-20, CA184024), in which ipilimumab has been approved for the treatment of melanoma in the United States.

CTLA-4 blockers have limited efficacy as a single agent and can non-selectively activate peripheral naive T cells at effective doses, resulting in greater toxicity. Approximately 15% of patients experienced serious or fatal irAEs (colitis, hepatitis, neuropathy, dermatitis, endocrinopathy, myocarditis, etc.) at 3 mg/kg after a total of 4 doses; this proportion of patients can reach about 40% at 10 mg/kg after a total of 4 doses [3].

1.1.3 PD-1/PD-L1 in combination with ctla-4 checkpoint inhibitors

PD-1/PD-L1 in combination with CTLA-4 checkpoint inhibitors have shown promising synergistic anti-tumor effects in the treatment of melanoma, MSI-H colorectal carcinoma, TMB-H NSCLC, and renal cell carcinoma, where nivolumab in combination with ipilimumab has been approved for the treatment of 1L melanoma and 1L intermediate-high-risk renal cell carcinoma and has become the standard 1L therapy for intermediate- and high-risk renal cell carcinoma. In order to reduce the immune-related toxicity of the combination therapy, lower doses of CTLA-4 were selected for each combination therapy.

In the study of CheckMate-067, 1,296 patients with newly diagnosed advanced malignant melanoma were divided into 3 groups in a ratio of 1:1:1 to receive nivolumab in combination with ipilimumab, nivolumab, or ipilimumab, respectively. The results showed that the median survival was greater than 40 months, 37.6 months, and 19.9 months in the combination treatment group, as well as the nivolumab and ipilimumab monotherapy groups, respectively, with 3-year survival rates

of 58%, 52%, and 34%, respectively, efficacy rates of 58%, 44%, and 19%, respectively, and 3-year PFS rates of 39%, 32%, and 10%, respectively. The incidences of Grade 3-4 adverse reactions (ARs) were 59%, 21%, and 28% in the 3 treatment groups, respectively [4].

847 untreated patients with intermediate- and high-risk advanced renal cell carcinoma were enrolled in CheckMate-214, who were randomized to nivolumab 3 mg/kg in combination with ipilimumab 1 mg/kg treatment group or the sunitinib treatment group. The results showed that the ORR was 42%, the median PFS was 11.6 months (95% CI: 8.7-15.5), and the median DOR was not reached in the combination group; the ORR was 27%, the median PFS was 8.4 months (95% CI: 7.0-10.8), and the DOR was 18.2 months in the control group [5].

Recently, data from Study Checkmate 227 showed that, for NSCLC patients with PD-L < 1% and TMB-H, nivolumab in combination with ipilimumab significantly prolonged PFS compared with nivolumab in combination with platinum-doublet chemotherapy or platinum-doublet chemotherapy monotherapy, with a 1-year PFS rate of up to 47% [6].

Although PD-1/PD-L1 in combination with CTLA-4 checkpoint inhibitors showed good synergistic effect, higher treatment-related toxicities limited the use and further development of this combination.

1.2 KN046

KN046 is a recombinant humanized novel bispecific antibody that binds both PD-L1 and CTLA-4, thereby blocking the binding of PD-L1 to PD-1 and CTLA-4 to CD80/CD86. KN046 is expressed and produced by CHO cells and the wild-type IgG1 Fc in the molecule maintains ADCC and CDC functions. CTLA-4 inhibitors act on naive T cells in secondary lymphoid organs and also mediate the depletion of Tregs, resulting in anti-tumor effects. PD-1/PD-L1 inhibitors can relieve inhibitory conduction pathways in the tumor microenvironment, thereby activating tumor-infiltrating CTLs. The binding of KN046 molecule to PD-L1 is stronger than that of CTLA-4 and has a stronger inhibitory effect on tumors with high expression of PD-L1, so KN046 can strongly activate the immune system in the tumor microenvironment. In clinical toxicology studies in cynomolgus monkeys, KN046 also showed good tolerability.

1.2.1 Preclinical PK study

A preclinical PK study of KN046 was conducted in cynomolgus monkeys. After intravenous (IV) administration of 1-100 mg/kg KN046 to cynomolgus monkeys, Cmax and AUC increased proportionally with the dose, with T1/2 of 51-88.3 h and CL of 0.701-0.747 mL/hr/kg; the mean accumulation rate after multiple doses was less than 2.0 (0.72-1.38).

PK information for KN046 is detailed in the Investigator's Brochure (IB).

1.2.2 Preclinical toxicology studies

Single-dose and multiple-dose toxicity studies of KN046 were conducted in non-human primates.

Single-dose toxicity: Single-dose toxicity was also observed in a 4-week multiple-dose toxicity study in cynomolgus monkeys. The results showed that the maximum tolerated dose (MTD) of a single dose of KN046 was approximately greater than 100 mg/kg.

Multiple-dose toxicity: In the 4-week multiple-dose toxicology study in cynomolgus monkeys, study drugs were administered intravenously at 0 mg/kg (control group), 10 mg/kg, 30 mg/kg, and 100 mg/kg weekly for a total of 5 doses, with a 6-week recovery period. In the 30 mg/kg group, 1 male died during the 5th dose. Pathological observations were performed on Days 18, 20, and/or 29, presumably due to immunogenic challenge with KN046, which had the highest ADA titer on Day 29, rather than a direct effect of KN046. KN046-related major changes were noted in the ≥ 30 mg/kg group, including enlarged groin nodes and decreased body weight and food consumption in 1 animal (at 100 mg/kg); pathological changes were mainly indicative of inflammatory responses, including slightly increased large unstained cell counts, mild to moderate increases in fibrinogen concentrations, and slight to mild increases in globulin concentrations; histopathologically noted ARs included multiorgan vascular inflammation, mesangioproliferative glomerulonephropathy and/or concomitant tubular degeneration/necrosis/regeneration, hypertrophy/hyperplasia of Kupffer cells, neutrophil/mononuclear cell infiltration in liver sinusoids and/or portal areas, and cardiomyocyte degeneration/necrosis (at ≥ 30 mg/kg). The no observed adverse effect level (NOAEL) for KN046 was 10 mg/kg.

Immunogenicity: In a single IV dose PK study in cynomolgus monkeys, 17/18 animals in all 3 groups (1.0 mg/kg, 3.0 mg/kg, or 10.0 mg/kg) were positive for ADAs 42 days postdose. Only 1 animal in the 3.0 mg/kg group was ADA-negative. In the multiple IV dose study in cynomolgus monkeys (10, 30, and 100 mg/kg weekly), 4/10 animals were ADA-positive on Day 29 and 4/10, 0/10, and 1/10 animals were ADA-positive on Day 43 in the three dose groups, respectively.

Hemolysis test showed that KN046 (26.3 mg/mL) did not cause hemolytic reactions in red blood cells of rabbits.

Toxicology study results are detailed in the IB.

1.2.3 Clinical studies

A Phase I, open-label, multiple-ascending dose-escalation and expansion Trial KN046-AUS-001 is currently ongoing to evaluate the maximum tolerated dose (MTD), recommended phase 2 doses (RP2Ds), biological effective dose (BED), the safety, tolerability and pharmacokinetics (PK) of intravenously administered KN046 in subjects with metastatic or locally advanced solid tumors.

Trial KN046-AUS-001 consists of a dose escalation phase (3+3 design) followed by an expansion phase. The dose escalation phase was designed to provide safety and tolerability, as well as PK data for KN046 at sequential doses ranging from 0.3 to 10.0 mg/kg in subjects with advanced malignancies, for which there are no established standard treatments. As of 20-Jan-2020, enrollment has been completed and 54 subjects have been treated with KN046.

The dose escalation (3 + 3 design) phase included a total of 13 subjects and was performed at the following dose levels:

- Dose level 1: 0.3 mg/kg (Cohort 1), 1 subject treated.
- Dose level 2: 1.0 mg/kg (Cohort 2), 3 subjects treated.
- Dose level 3: 3.0 mg/kg (Cohort 3), 3 subjects treated.
- Dose level 4: 5.0 mg/kg (Cohort 4), 6 subjects treated.
- Dose level 5: 10.0 mg/kg (Cohort 5), 3 subjects treated.

The 3 + 3 dose escalation algorithm to determine the MTD/RP2Ds/BED is complete and doses of 3.0 and 5.0 mg/kg once every 2 weeks was determined for the tumor treatment expansion cohorts on the basis of safety, PK, preliminary efficacy and *ex vivo* pharmacodynamics observations.

As of 20-Jan-2020, the dose expansion phase included a total of 14 subjects at 3.0 mg/kg dose level and 24 subjects at 5.0 mg/kg dose level.

- Expansion cohort 1: 3 mg/kg, 14 subjects treated
- Expansion cohort 2: 5 mg/kg, 24 subjects treated

1.2.3.1 Safety results

As of 20-Jan-2020, a total of 54 subjects were included in the safety data analysis, including 16 and 37 subjects enrolled in the dose escalation and dose expansion, respectively. Median duration of therapy was 11 weeks (range: 2 to 67). In total 13.0% patients discontinued KN046 treatment due to adverse event.

Four DLT events were observed in three subjects, including (i) one subject with grade 3 treatment-related hepatic function abnormal without bilirubin increased from the 5.0 mg/kg Q2W cohort; and (ii) one subject with grade 3 pruritic erythematous rash, and one subject with grade 3 aspartate aminotransferase increased accompanied by grade 3 arthritis from the 10.0 mg/kg Q2W cohort. The relevant subjects recovered within three weeks. Maxima tolerate dose (MTD) has been declared at 5 mg/kg Q2W.

As of the Data Cut-off Date, 41 (75.9%) out of the 54 subjects had experienced treatment-related TEAE of all grades, and 20 (37.0%) subjects had experienced treatment- related TEAEs at grade 3 or higher levels. 14 (25.9%) subjects had experienced treatment- related SAEs and 26 (48.1%) subjects had experienced irAEs, 13 (24.1%) of which were grade 3 or higher levels. The most frequent treatment-related TEAEs (TRAE) included arthralgia (16.7%), fatigue (14.8%), infusion-related reaction (14.8%), diarrhea (11.1%) and pruritus (11.1%. Skin and subcutaneous tissue disorders and musculoskeletal and connective tissue disorders were the most frequent irAEs. The TRAE and irAEs were not found to occur in a dose-dependent manner up to 5 mg/kg Q2W, and neither the number nor severity of TRAE or irAEs was exacerbated due to dose escalation at the RP2D or lower levels.

4 subjects with thymic neoplasms were enrolled in KN046-AUS-001. 2 out 4 patients are still under treatment (340+ and 240+ days). 1 patient (BOR SD) had spinal injury and off treatment due to this treatment unrelated AE. The patient had been on treatment for 146 days before KN046 withdrawal. One patient had baseline ocular MG as paraneoplastic disease to underlying advanced thymoma and enrolled (previous the patient did not perform thymectomy). After 8 KN046 doses (Q2W), the patient developed ocular symptoms and then generalized symptoms and requiring intensive care. The investigator considered this SAE as disease related due to rapid elevation of anti-acetycholine receptor levels indicating antibody mediated rather T cell mediated which was thought unusual to checkpoint inhibitor related. Imaging at that time showed slow and steady increase (having 13% increase in target lesion from baseline) and lack of other organ specific immune related adverse events. The patient was withdrawn from KN046 treatment and developed partial response thereafter. This SAE was completely recovered.

Table 4 Subject disposition for Trial KN046-AUS-001 (Safety Population)

	0.3 mg/kg Q2W (N = 1)	1.0 mg/kg Q2W (N = 3)	3.0 mg/kg Q2W (N = 17)	5.0 mg/kg Q2W (N = 30)	10.0 mg/kg Q2W (N = 3)	Total (N = 54)
Subjects enrolled	1 (100%)	3 (100%)	17 (100%)	30 (100%)	3 (100%)	54 (100%)
Subjects dosed	1 (100%)	3 (100%)	17 (100%)	30 (100%)	3 (100%)	54 (100%)
Treatment ongoing at data cut-off	0	0	1 (5.9%)	7 (23.3%)	0	8 (14.8%)
Treatment termination	1 (100%)	3 (100%)	15 (88.2%)	22 (73.3%)	3 (100%)	44 (81.5%)
Confirmed disease progression	1 (100%)	2 (66.7%)	10 (58.8%)	12 (40.0%)	1 (33.3%)	26 (48.1%)
Intercurrent illness that prevents further administration of treatment	0	0	0	1 (3.3%)	0	1 (1.9%)
Unacceptable adverse experiences	0	1 (33.3%)	0	5 (16.7%)	1 (33.3%)	7 (13.0%)
Subject withdraws consent	0	0	1 (5.9%)	1 (3.3%)	1 (33.3%)	3 (5.6%)
If in the opinion of the Investigator, a change or discontinuation of therapy would be in the best interest of the subject	0	0	3 (17.6%)	2 (6.7%)	0	5 (9.3%)
Death	0	0	1 (5.9%)	0	0	1 (1.9%)
Other	0	0	0	1 (3.3%)	0	1 (1.9%)

Note: 1. Percentages are based on the number of subjects of each dose level in the evaluable analysis set.

Program: t_tte.sas Executed: 20FEB2020 23:24

Table 5 Safety summary for Trial KN046-CHN-001 (Safety Population)

	0.3 mg/kg Q2W (N = 1)	1.0 mg/kg Q2W (N = 3)	3.0 mg/kg Q2W (N = 17)	5.0 mg/kg Q2W (N = 30)	10 mg/kg Q2W (N = 3)	Total (N = 54)
Number of TEAE	5	35	153	271	34	498
Subjects with at least 1 TEAE	1 (100%)	3 (100%)	17 (100%)	28 (93.3%)	3 (100%)	52 (96.3%)
Related to KN046	1 (100%)	2 (66.7%)	13 (76.5%)	22 (73.3%)	3 (100%)	41 (75.9%)
Subjects with at least 1 CTCAE Grade ⩾3 TEAE	0	2 (66.7%)	11 (64.7%)	19 (63.3%)	3 (100%)	35 (64.8%)
Related to KN046	0	2 (66.7%)	4 (23.5%)	11 (36.7%)	3 (100%)	20 (37.0%)
Subjects with at least 1 DLT Event	0	0	0	1 (3.3%)	2 (66.7%)	3 (5.6%)
Subjects with at least 1 irAE	0	2 (66.7%)	9 (52.9%)	12 (40.0%)	3 (100%)	26 (48.1%)
Subjects with at least 1 CTCAE Grade ⩾3 irAE	0	1 (33.3%)	3 (17.6%)	6 (20.0%)	3 (100%)	13 (24.1%)
Subjects with at least 1 Infusion Reaction	0	1 (33.3%)	3 (17.6%)	4 (13.3%)	0	8 (14.8%)
Subjects with at least 1 CTCAE Grade ⩾3 Infusion Reaction	0	0	0	0	0	0
Subjects with at least 1 Treatment-emergent SAE	0	1 (33.3%)	9 (52.9%)	14 (46.7%)	2 (66.7%)	26 (48.1%)
Related to KN046	0	1 (33.3%)	4 (23.5%)	7 (23.3%)	2 (66.7%)	14 (25.9%)
Subjects with at least 1 CTCAE Grade ⩾3 Treatment-emergent SAE	0	1 (33.3%)	7 (41.2%)	12 (40.0%)	1 (33.3%)	21 (38.9%)
Related to KN046	0	0	3 (17.6%)	6 (20.0%)	1 (33.3%)	10 (18.5%)
Subjects with at least 1 TEAE Leading to Drug Withdrawn	0	1 (33.3%)	1 (5.9%)	7 (23.3%)	1 (33.3%)	10 (18.5%)
Related to KN046	0	1 (33.3%)	1 (5.9%)	4 (13.3%)	1 (33.3%)	7 (13.0%)
Subjects with TEAE Leading to Death	0	0	0	2 (6.7%)	0	2 (3.7%)
Related to KN046	0	0	0	0	0	0

Note: 1. Percentages are based on the number of subjects of each dose level in the safety analysis set.; 2. MedDRA 22.0; 3. CTCAE 4.03

Program: t_ae_pt.sas Executed: 20FEB2020 23:21

Table 6 Treatment related TEAEs (TRAEs) for Trial KN046-AUS-001 (Safety Population)

	0.3 mg/kg Q2W (N = 1)		1.0 mg/kg Q2W (N = 3)		3.0 mg/kg Q2W (N = 17)		5.0 mg/kg Q2W (N = 30)		10.0 mg/kg Q2W (N = 3)		Total (N = 54)	
Preferred Term	All grades	Grade≥3	All grades	Grade≥3	All grades	Grade≥3	All grades	Grade≥3	All grades	Grade≥3	All grades	Grade≥3
Subjects with at least 1 KN046 related TEAE	1 (100%)	0	2 (66.7%)	2 (66.7%)	13 (76.5%)	4 (23.5%)	22 (73.3%)	11 (36.7%)	3 (100%)	3 (100%)	41 (75.9%)	20 (37.0%)
Arthralgia	0	0	1 (33.3%)	0	4 (23.5%)	0	4 (13.3%)	1 (3.3%)	0	0	9 (16.7%)	1 (1.9%)
Fatigue	0	0	0	0	0	0	7 (23.3%)	0	1 (33.3%)	1 (33.3%)	8 (14.8%)	1 (1.9%)
Infusion related reaction	0	0	0	0	4 (23.5%)	0	4 (13.3%)	1 (3.3%)	0	0	8 (14.8%)	1 (1.9%)
Diarrhoea	0	0	0	0	2 (11.8%)	0	2 (6.7%)	0	2 (66.7%)	0	6 (11.1%)	0
Pruritus	0	0	0	0	3 (17.6%)	1 (5.9%)	3 (10.0%)	0	0	0	6 (11.1%)	1 (1.9%)
Alanine aminotransferase increased	0	0	0	0	1 (5.9%)	0	1 (3.3%)	1 (3.3%)	2 (66.7%)	0	4 (7.4%)	1 (1.9%)
Flushing	0	0	0	0	1 (5.9%)	0	3 (10.0%)	1 (3.3%)	0	0	4 (7.4%)	1 (1.9%)
Nausea	0	0	1 (33.3%)	0	1 (5.9%)	0	1 (3.3%)	1 (3.3%)	1 (33.3%)	0	4 (7.4%)	1 (1.9%)
Pyrexia	0	0	1 (33.3%)	0	0	0	2 (6.7%)	0	1 (33.3%)	0	4 (7.4%)	0
Aspartate aminotransferase increased	0	0	0	0	0	0	1 (3.3%)	0	2 (66.7%)	1 (33.3%)	3 (5.6%)	1 (1.9%)
Hyperthyroidism	0	0	1 (33.3%)	0	1 (5.9%)	0	1 (3.3%)	0	0	0	3 (5.6%)	0
Myalgia	0	0	0	0	1 (5.9%)	0	2 (6.7%)	0	0	0	3 (5.6%)	0
Rash	0	0	0	0	0	0	3 (10.0%)	0	0	0	3 (5.6%)	0
Transaminases increased	0	0	0	0	1 (5.9%)	0	2 (6.7%)	0	0	0	3 (5.6%)	0
Abdominal pain	0	0	0	0	0	0	1 (3.3%)	0	1 (33.3%)	0	2 (3.7%)	0

Note: 1. Percentages are based on the number of subjects of each dose level in the safety analysis set.； 2. MedDRA 22.0； 3. CTCAE 4.03

Program: t_ae_pt.sas Executed: 20FEB2020 23:21

Table 7 Immune-related AEs for Trial KN046-AUS-001 (Safety Population)

	0.3 mg/kg Q2W (N = 1)		1.0 mg/kg Q2W (N = 3)		3.0 mg/kg Q2W (N = 17)		5.0 mg/kg Q2W (N = 30)		10.0 mg/kg Q2W (N = 3)		Total (N = 54)	
Preferred Term	All grades	Grade≥3	All grades	Grade≥3	All grades	Grade≥3	All grades	Grade≥3	All grades	Grade≥3	All grades	Grade≥3
Subjects with at least 1 irAE	0	0	2 (66.7%)	1 (33.3%)	9 (52.9%)	3 (17.6%)	12 (40.0%)	6 (20.0%)	3 (100%)	3 (100%)	26 (48.1%)	13 (24.1%)
Arthralgia	0	0	1 (33.3%)	0	2 (11.8%)	0	2 (6.7%)	1 (3.3%)	0	0	5 (9.3%)	1 (1.9%)
Infusion related reaction	0	0	0	0	3 (17.6%)	0	2 (6.7%)	0	0	0	5 (9.3%)	0
Pruritus	0	0	0	0	3 (17.6%)	0	1 (3.3%)	0	0	0	4 (7.4%)	0
Transaminases increased	0	0	0	0	1 (5.9%)	0	3 (10.0%)	0	0	0	4 (7.4%)	0
Alanine aminotransferase increased	0	0	0	0	0	0	1 (3.3%)	1 (3.3%)	1 (33.3%)	0	2 (3.7%)	1 (1.9%)
Aspartate aminotransferase increased	0	0	0	0	0	0	1 (3.3%)	0	1 (33.3%)	0	2 (3.7%)	0
Autoimmune arthritis	0	0	0	0	1 (5.9%)	1 (5.9%)	1 (3.3%)	0	0	0	2 (3.7%)	1 (1.9%)
Autoimmune myositis	0	0	0	0	0	0	2 (6.7%)	2 (6.7%)	0	0	2 (3.7%)	2 (3.7%)
Diarrhoea	0	0	0	0	2 (11.8%)	0	0	0	0	0	2 (3.7%)	0
Hepatic function abnormal	0	0	1 (33.3%)	1 (33.3%)	0	0	1 (3.3%)	1 (3.3%)	0	0	2 (3.7%)	2 (3.7%)
Hepatitis	0	0	0	0	1 (5.9%)	0	1 (3.3%)	0	0	0	2 (3.7%)	0
Hyperthyroidism	0	0	1 (33.3%)	0	1 (5.9%)	0	0	0	0	0	2 (3.7%)	0
Myalgia	0	0	0	0	1 (5.9%)	0	1 (3.3%)	0	0	0	2 (3.7%)	0
Myositis	0	0	0	0	0	0	1 (3.3%)	0	1 (33.3%)	0	2 (3.7%)	0

Note: 1. Percentages are based on the number of subjects of each dose level in the safety analysis set.； 2. MedDRA 22.0； 3. CTCAE 4.03

Program: t_ae_pt.sas Executed: 20FEB2020 23:21

1.2.3.2 Efficacy results

In general, all of the subjects enrolled in this study had previously failed standard-of-care treatments. As of the Data Cut-off Date, there were 35 evaluable subjects. The efficacy results showed that among the 35 evaluable subjects, the ORR (defined as the proportion of subjects with a BOR of confirmed or unconfirmed CR or PR), was 16.7% (95% CI 7.0, 31.4). At recommended Phase 2 dose levels (3 mg/kg Q2W and 5 mg/kg Q2W), the ORR was 15.4% (95% CI 1.9, 45.4) and 20.8% (95% CI 7.1, 42.2), respectively.

4 subjects with thymic neoplasms were enrolled in KN046-AUS-001 (stage IV thymoma 2; thymic carcinoma 2). 3 subjects observed complete response (n = 1) or partial response (n = 2). 1 thymoma patient observed stable disease. KN046 showed preliminary efficacy in this study population.

Table 8 Efficacy Summary for Trial KN046-AUS-001 (Efficacy Population)

	0.3 mg/kg Q2W (N = 1)	1.0 mg/kg Q2W (N = 3)	3.0 mg/kg Q2W (N = 13)	5.0 mg/kg Q2W (N = 24)	10.0 mg/kg Q2W (N = 1)	Total (N = 42)
Best Overall Response						
Complete Response (CR)	0	0	1 (7.7%)	0	0	1 (2.4%)
Partial Response (PR)	0	0	0	2 (8.3%)	0	2 (4.8%)
Unconfirmed Complete Response (uCR)	0	0	0	0	0	0
Unconfirmed Partial Response (uPR)	0	0	1 (7.7%)	3 (12.5%)	0	4 (9.5%)
Stable Disease (SD)	0	1 (33.3%)	2 (15.4%)	12 (50.0%)	0	15 (35.7%)
Progressive Disease (PD)	1 (100%)	2 (66.7%)	9 (69.2%)	7 (29.2%)	1 (100%)	20 (47.6%)
Objective Response Rate (ORR)	0	0	2 (15.4%)	5 (20.8%)	0	7 (16.7%)
95% CI	0.0%, 97.5%	0.0%, 70.8%	1.9%, 45.4%	7.1%, 42.2%	0.0%, 97.5%	7.0%, 31.4%
Disease Control Rate (DCR)	0	1 (33.3%)	4 (30.8%)	17 (70.8%)	0	22 (52.4%)
95% CI	0.0%, 97.5%	0.8%, 90.6%	9.1%, 61.4%	48.9%, 87.4%	0.0%, 97.5%	36.4%, 68.0%

Note: 1. Percentages are based on the number of subjects of each dose level in the evaluable analysis set.
2. ORR=CR+PR+uCR+uPR.
3. DCR=CR+PR+uCR+uPR+SD.

Program: t_rs.sas Executed: 20FEB2020 23:23

1.2.3.3 Pharmacokinetic results

Trial KN046-AUS-001 was performed in Australia and enrolled majority Caucasians.

Below table displays the main PK parameters that were evaluated after the first dose in Trial KN046-AUS-001. After single dose, terminal half-life does not appear to be dose-dependent and ranges from 100.8 to 181.8 hours across dose range of 3.0 to 10 mg/kg. Formal dose proportionality has not been performed. From exploratory analysis, it appears both C_{max} and AUC_{inf} increase with increasing dose across the dose range of 0.3 to 10.0 mg/kg.

Table 9 Summary of Pharmacokinetic Parameters on Cycle 1 Day 1 (KN046-AUS-001)

PK parameters	0.3 mg/kg Q2W (N=1)	1.0 mg/kg Q2W (N=3)	3.0 mg/kg Q2W (N=17)	5.0 mg/kg Q2W (N=16)	10.0 mg/kg Q2W (N=2)
**AUC_{inf} (ng*h/mL)**					
Mean (SD)	505552	2463669 (712624.2)	8520038.8 (2472946.6)	13675607 (3908026.9)	17917942 (3326685.7)
Median	505552	2540594	7956885	13712441	17917942
Geometric Mean	505552	2390699.1	8198420.7	13141043	17762861
**C_{max} (ng/mL)**					
Mean (SD)	6446	25586.7 (10153.2)	96308.8 (96041.7)	119118.7 (41359.7)	148228 (48202.1)
Median	6446	25292	71716	105547	148228
Geometric Mean	6446	24183.6	79423.8	113315.1	144256.1
**Terminal $T_{1/2}$ (h)**					
Mean (SD)	59	100.8 (19.9)	165.5 (45.1)	181.8 (52.5)	170.5 (0.7)
Median	59	98	164	183.5	170.5
Geometric Mean	59	99.5	159.1	172.7	170.5
**CL (mL/h)**					
Mean (SD)	35.6	30.0 (1.7)	28.6 (7.2)	26.3 (6.2)	31.0 (5.4)
Median	35.6	30.3	27.7	25.9	31.0
Geometric Mean	35.6	29.9	27.8	25.6	30.7

2 RATIONALE FOR THE CURRENT CLINICAL TRIAL

2.1 Rationale for dose selection

The predetermined initial dose of KN046 for this study were 3 mg/kg. The rationale for dose selection is mainly based on the safety and preliminary efficacy data from the KN046-AUS-001 study (first-in-human study of KN046). The KN046 3 mg/kg Q2W showed good tolerance in humans, and 1 NSCLC subject observed CR and 1 ovarian cancer subject observed PR (target lesion shrinkage > 50%) after treatment in this dose cohort. At the time of 3 mg/kg Q2W dosing, the steady-state trough concentration had exceeded approximately 20 times the drug concentration corresponding to the maximum release effect of IL-2 in vitro, which also supports that the 3 mg/kg dose is an effective pharmacodynamics dose.

2.2 Rationale for clinical endpoint

The primary measure of antitumor activity is an estimation of overall response rate (ORR) and duration of response (DOR) according to RECIST 1.1. Confirmation of responses by independent review committee (IRC) are required to prevent bias in tumor assessment. For early phase clinical studies, ORR and DOR are acceptable surrogate endpoints for measuring clinical benefits, which can reflect the drug's anti-tumor activity, especially when durable anti-tumor responses can be observed.

2.3 Rationale for indication selection

Subjects with triple negative breast cancer (TNBC) will be enrolled in this study. Breast cancer is the most common malignant tumor in women, with an estimated 272,400 new cases of breast cancer diagnosed in China each year, resulting in 70,700 deaths from breast cancer [8]. TNBC is the most aggressive subtype of breast cancer, accounting for 15% to 20% of the breast cancer population. The median survival period for metastatic TNBC is less than 12 months, hence there exists a substantial medical need in this area.

Recent molecular subtyping has suggested that TNBC is a highly heterogeneous disease, with approximately 40% of TNBC tumors expressing PD-L1 on immune cells (≥1% tumor immune cell PD-L1 expression positive) [10]. Immune checkpoint blockers targeting PD1 and PD-L1 have demonstrated good efficacy and safety in TNBC. In a phase 1 study of atezolizumab involving 116 TNBC patients, those who received atezolizumab monotherapy had an objective response rate of 24% (5/21) for first-line TNBC patients and 6% (6/94) for second-line or higher TNBC patients; among patients whose tumor immune cells expressed PD-L1, the objective response rate was 12% (11/91), while it was 0% for those without PD-L1 expression; the median duration of response was 19-21 months [11]. In a phase 1 study of pembrolizumab involving 170 first-line or higher TNBC patients and 52 first-line TNBC patients, approximately 62% of TNBC tumors or immune cells expressed PD-L1 (≥1% tumor or tumor immune cell PD-L1 expression positive). The ORR of pembrolizumab monotherapy was 23% for first-line TNBC patients and 4.7% for ≥second-line patients, with disease control rate (DCR) of 40% and median DOR of 8.4 months for 1L TNBC patients; the DCR was 25.3%, with median DOR of 6.3 months for ≥2L TNBC patients, and the median OS for these disease control patients had not yet been reached [12].

A recent phase 3 study of atezolizumab involving 451 treatment-naive TNBC patients to compare the efficacy of atezolizumab plus nab-paclitaxel versus nab-paclitaxel monotherapy. The addition of atezolizumab to nab-paclitaxel significantly prolonged the progression-free survival (PFS, 7.2 months vs 5.5 months, HR 0.8, p=0.002), and in patients with PD-L1 positive status, the PFS was also significantly longer than that of nab-paclitaxel monotherapy (7.5 months vs 5.0 months, HR 0.62, p<0.001). At the interim analysis, the overall survival was significantly longer in the atezolizumab plus nab-paclitaxel arm than in the nab-paclitaxel monotherapy arm for patients with PD-L1 positive status (25.0 months vs 15.5 months, HR 0.62). The safety profile of atezolizumab plus nab-paclitaxel was also manageable, with a discontinuation rate due to adverse events of 15.9%. Therefore, atezolizumab plus nab-paclitaxel is expected to become a new standard treatment for TNBC [10].

KN046 is a bispecific antibody to PD-L1 and CTLA-4. PD-1 and CTLA-4 have different mechanisms in preventing T cell exhaustion and promoting T cell activation, and they can produce complementary effects in regulating immune responses. The inhibitory effect of CTLA-4 on T cell activation and proliferation occurs during the antigen presentation process, while the inhibitory effect of the PD-1 pathway occurs at the tumor site. Targeting both pathways can produce additive or synergistic effects [7], so combination therapy may produce better anti-tumor effects than monotherapy. Simultaneous inhibition of PD-L1 and CTLA4 has shown stronger anti-tumor activity in multiple tumor types, including melanoma, non-small cell lung cancer, and renal cell carcinoma. In a preclinical study, compared to chemotherapy combined with PD-L1 or CTLA4 monoclonal antibody blockers, chemotherapy combined with dual immunoblockade of PD-L1 and CTLA4 in BRCA1/2 mutated negative TNBC mice significantly increased the infiltration of tumor effector T cells (cytotoxic CD8+ and CD4+ T cells) and significantly prolonged mouse survival [13]. PD-L1 combined with CTLA4 immune checkpoint blockers have also shown preliminary efficacy in humans. In a single-arm study, 18 patients with metastatic breast cancer (HR+ breast cancer n=11; TNBC n=7) were treated with durvalumab plus tremelimumab. Among the TNBC patients, ORR was achieved in 3/7 (43%) after treatment, with DOR exceeding 10 months. 1 TNBC patient had SD for more than 12 weeks, and another TNBC patient had SD after progression with all target lesions responding and enlarged lymph nodes remaining stable [20].

This study will be the first to explore the efficacy of KN046 as a single agent or in combination with nab-paclitaxel in TNBC patients, with the aim to expand the benefit population of PD-1 or PD-L1 immune checkpoint inhibitor monotherapy and explore the impact of biomarkers, such as PD-L1 expression, TMB, BRCA1/2 mutations, HRD mutations, and TILs, on the efficacy of KN046.

2.4 Rational for chemotherapy selection

Taxane are currently the standard first-line treatment for metastatic breast cancer, including TNBC. Taxane combination with other chemotherapy is mostly used in patients with invasive disease or visceral metastasis, but there is no significant survival benefit compared with single agent. Nab-paclitaxel greatly reduces the high sensitivity reaction of paclitaxel solvent-related by attaching paclitaxel to albumin to form particles of a mean 130 nm in diameter. It does not need premedication and shorten the infusion time to about 30 minutes. Preclinical studies have shown that nab-paclitaxel can be enriched in tumor tissues, and the drug exposure level in tumors is about 33% higher than that of paclitaxel [14]. A phase III study compared the efficacy of nab-paclitaxel and paclitaxel in

patients with metastatic breast cancer (n=460). The objective response rate in the a nab-paclitaxel treatment arm was higher (21.5% vs 11.1%), the median PFS was longer (23.0 weeks vs 16.9 weeks), and the incidence of grade 4 neutropenia was lower (9% vs 22%). The incidence of grade 3 sensory neuropathy was higher in the nab-paclitaxel arm (10% vs 2%), but it was self-limiting and improved after drug suspension or dose reduction [15]. In a key phase III study, patients with metastatic breast cancer who did not receive prior paclitaxel treatment, or patients with advanced breast cancer who recurred more than 12 months after the completion of paclitaxel adjuvant treatment were enrolled to compare the efficacy of nab-paclitaxel and paclitaxel. The ORR of nab-paclitaxel was higher (34% vs 18%, p=0.013), and the PFS tended to be prolonged (23.7 weeks vs 19.7 weeks, p=0.173), but the total OS was not significant difference between the two treatment arms (71.0 weeks vs 77.9 weeks, HR=1.215, p=0.264). Based on the phase III study, nab-paclitaxel was approved for breast cancer patient with metastatic disease or relapse within 6 months of adjuvant chemotherapy [16, 17]. Although nab-paclitaxel has not been officially approved for first-line treatment of metastatic breast cancer, NCCN guidelines have listed it as a first-line recommended regimen [18].

2.5 Overall benefit and risk assessment

The occurrence of AEs will be closely observed in the clinical study, including vital signs, ECG, laboratory blood, and urine tests. Based on preclinical studies of KN046 and clinical results of marketed anti-PD-L1 and anti-CTLA-4 monoclonal antibodies with the same target, the most common ARs included weariness, infusion reaction, diarrhoea, joint pain, skin rash, nausea, pruritus, and headache, most of which were mild to moderate in severity.

Autoimmune-related toxicities may occur during drug treatment with anti-PD-L1 and anti-CTLA-4 monoclonal antibodies, therefore careful monitoring and special therapeutic measures are required. These drug-related AEs included skin rash and pruritus in the skin system, diarrhoea and colitis in the gastrointestinal system, hypophysitis, hepatitis, endocrine disorders, pneumonia, and renal insufficiency. In this study, principles for the management of immune-related toxicities will be developed with reference to relevant ESMO and NCCN guidelines [6]. Measures such as exclusion criteria, safety monitoring, initial dose design, treatment interruption provisions, discontinuation criteria, and management of immune-related toxicities will be taken to reduce the potential risks of subjects (Section 6.1.4, Appendix 2).

The management of potential infusion reactions during use of KN046 as a biological product is described in Section 6.3.3.1. As ADCC function is retained in the structure of KN046, the management of potential tumor lysis syndrome is provided in Section 6.3.3.2.

The SMC will be established to monitor the benefits and risks of the study on an ongoing basis (Section 4.4), and the study will be suspended or terminated in the event of unexpected and unacceptable adverse events (Section 4.2).

The study will be conducted in compliance with the protocol, GCP, the Declaration of Helsinki and other relevant regulations.

3 TRIAL OBJECTIVES AND ENDPOINTS

3.1 Trial objectives

3.1.1 Primary objective

- To evaluate the anti-tumor activity of KN046 monotherapy.
- To evaluate the anti-tumor activity of combination of KN046 and nab-paclitaxel.

3.1.2 Secondary objectives

- To evaluate the safety and tolerability of KN046 monotherapy.
- To evaluate the safety and tolerability of the KN046 combination therapy.
- To evaluate the immunogenicity of KN046.
- To characterize the pharmacokinetics of KN046 and evaluate the effect of nab-paclitaxel on the pharmacokinetics of KN046.
- To evaluate the impact of biomarkers (PD-L1 expression, BRCA1/2 mutations, HRD, TMB, TIL) on the anti-tumor activity of KN046.
- To evaluate the correlation between drug exposure levels and anti-tumor activity of KN046.

3.1.3 Exploratory objective

- To explore the correlation between KN046 drug exposure and safety.

3.2 Trial endpoints

3.2.1 Primary endpoint

- ORR and DOR per RECIST v1.1 by IRC.

3.2.2 Secondary endpoints

- ORR and DOR per RECIST v1.1 by investigator.
- 6-months PFSR, 12-months PFSR, CBR, defined as the proportion of subjects with best overall response of CR, PR, or SD ⩾ 24 weeks), per RECIST v1.1 by IRC and investigator.
- 6-months OS rate and 12-months OS rate.
- Incidence and severity (as graded by CTCAE v5.0), seriousness and relationship to the trial treatments, abnormal findings on any laboratory test and physical examination.

- Status (positive or negative) and serum titers of anti-KN046 antibody and neutralizing capacity.
- Concentration-time profiles of KN046 and individual PK parameters of KN046 derived from population PK analysis, including but not limited to AUC_{ss}, $C_{max,ss}$, $C_{min,ss}$, CL and $T_{1/2}$.
- The correlation between biomarkers (PD-L1 expression, BRCA1/2 mutations, HRD, TMB) and clinical efficacy parameters (ORR, CBR, PFSR).
- The correlation between pharmacokinetic parameters ($AUC_{tau,ss}$, $C_{trough,ss}$, etc.) of KN046 and clinical efficacy parameters (ORR, CBR, PFSR, etc.).

3.2.3 Exploratory endpoints

- The correlation between pharmacokinetic parameters (AUCtau,ss, Ctrough,ss, etc.) of KN046 and safety indicators.

4 STUDY DESIGN

4.1 Overall study design and plan

This study is a Ib/II, multicenter, open-label clinical trial designed to evaluate the efficacy and safety of KN046 monotherapy or combination with nab-paclitaxel in patients with metastatic or locally advanced unresectable triple-negative breast cancer (TNBC) in China. The study will consist of two stages, does-escalation stage, and dose-expansion stage. Each subject will submit tumor tissue samples to determine tumor and immune cell biomarkers such as BRCA1/2 mutation status, HRD mutation status, tumor mutational burden (TMB), PD-L1 expression level, and TIL.

Subjects who have received at least one prior line of systemic treatment will receive KN046 monotherapy. The monotherapy cohort will initially enroll 6 subjects and allocated to 3 mg/kg IV Q2W according to the protocol requirements. After 6 subjects have been enrolled and have completed a 28-day safety observation period, a Safety Monitoring Committee (SMC) meeting will be held (enrollment will not be paused during this period). The safety, preliminary efficacy, and pharmacokinetic data of KN046 will be reviewed, and the following decisions will be made: 1) expand the 3 mg/kg dose group to 30 subjects; and/or 2) dose escalates to 5 mg/kg IV Q2W treatment group (subjects in the 3 mg/kg Q2W cohort who do not experience Grade ⩾ 2 treatment-related adverse events before the SMC meeting can escalate to 5 mg/kg IV Q2W with the consent of the investigator and subjects). The 5 mg/kg IV Q2W cohort will initially enroll 6 subjects. After 6 subjects have been enrolled and have completed a 28-day safety observation period, an SMC meeting will be held (enrollment will not be paused during this period). The safety, preliminary efficacy, and pharmacokinetic data of KN046 will be reviewed, and a decision will be made on whether to continue expanding the 5 mg/kg dose group to 30 subjects (Figure 1). Each subject will receive KN046 (predefined dosing regimen is 3 mg/kg or 5 mg/kg IV, Q2W), until disease progression (assessed by the investigator based on RECIST1.1), intolerable toxicity, withdrawal of consent, or completion of 2-years treatment, whichever occurs first. After each cohort of the KN046 monotherapy has enrolled 30 subjects and all subjects have completed at least one post-baseline imaging, a mid-term analysis will be conducted. After discussion by the SMC, a decision will be made on whether to further expand in the original cohort or in the enriched population of biomarker-positive individuals or explore higher dose levels and/or different dosing intervals.

Subjects naïve to systemic anti-cancer treatment will receive combination therapy with KN046 and nab-paclitaxel. The combination treatment cohort will initially enroll 6 subjects, who will receive KN046 at a dose of 3 mg/kg IV Q2W in combination with albumin-bound paclitaxel as per the protocol. After 6 subjects have been enrolled and have completed a 28-day safety observation period, an SMC meeting will be held (enrollment will not be paused during this period). The safety, preliminary efficacy, and pharmacokinetic data of KN046 will be reviewed, and the following decisions will be made: 1) expand the 3 mg/kg dose group to 30 subjects; and/or 2) dose escalates to KN046 5 mg/kg IV Q2W treatment group (subjects in the 3 mg/kg Q2W cohort who do not experience Grade ≥ 2 treatment-related adverse events before the SMC meeting can escalate to 5 mg/kg IV Q2W with the consent of the investigator and subjects). The 5 mg/kg IV Q2W cohort will initially enroll 6 subjects. After 6 subjects have been enrolled and have completed a 28-day safety observation period, an SMC meeting will be held (enrollment will not be paused during this period).

The safety, preliminary efficacy, and pharmacokinetic data of combination therapy will be reviewed, and a decision will be made on whether to continue expanding the KN046 5 mg/kg dose group to 25 subjects (Figure 2). Each subject will receive KN046 (predefined dosing regimen is 3 mg/kg or 5 mg/kg IV, d1,15, Q4W) and nab- paclitaxel (100 mg/m2, d1,8,15, Q4W), until disease progression (assessed by the investigator based on RECIST1.1), intolerable toxicity, withdrawal of consent, or completion of 2-years treatment, whichever occurs first. The nab-paclitaxel is prescribed for 6 cycles (28-day/cycle) initially. After subjects completing these 6 cycles, investigators will evaluate the need for ongoing treatment with nab-paclitaxel based on medical best practices and a thorough assessment of the potential benefits and risks. After each cohort of the KN046 combination therapy has enrolled 25 subjects and all subjects have completed at least one post-baseline imaging, a mid-term analysis will be conducted. After discussion by the SMC, a decision will be made on whether to further expand in the original cohort or in the enriched population of biomarker-positive individuals or explore higher dose levels and/or different dosing intervals.

Tumor evaluation will be performed at baseline, every 8 weeks (56-days ± 7-days) within 12 months and every 12 weeks (84-days ± 7-days) thereafter. Tumor evaluation will continue until confirmed progressive disease per RECIST 1.1, starting new anti-cancer therapy, withdrawal of informed consent, or subject dies whichever comes first. Once objective response is observed, response should be confirmed by a second scan at approximately 8 weeks apart (no earlier than 4 weeks and no later than 8 weeks).

If a subject is clinically stable and has not experienced intolerable toxicity during KN046 treatment upon first RECIST 1.1 defined PD judged by the investigator, the subject is allowed to continue receiving treatment until confirmed disease progression (Section 6.1.3). Clinical stability is defined as: stable ECOG score, absence of unacceptable toxicity related to KN046 treatment (Section 6.1.4), absence of rapid disease progression requiring salvage therapy, and no emergent medical interventions required due to disease progression (such as central nervous system metastasis, tumor-induced airway obstruction leading to respiratory distress, or spinal cord compression). For subjects with bone metastases who were receiving denosumab during the screening period, it is required to switch to bisphosphonate to treat bone metastases before starting KN046 treatment.

An SMC will be established to review the emerging safety and efficacy data from this study. Based on safety, efficacy and/or pharmacokinetics data from this trial and/or other KN046-related trials, the SMC will decide whether to increase the intermediate KN046 dose, continue expansion in a certain dose and / or treatment cohort, terminate a certain dosing schedule and / or treatment cohort, add other dosing interval cohorts, and / or explore higher KN046 dosing based on safety, pharmacokinetics, and / or other data from previous treatment cohorts and / or other studies of KN046.

Each subject will receive study treatment as per the protocol until progressive disease (PD) as judged by the investigator per Response Evaluation Criteria in Solid Tumors (RECIST) V1.1, intolerable toxicity, withdrawal of consent by the subject, or treatment for 2 years, whichever occurs first.

The study period includes a screening period (Day -21 to Day 0), a treatment period (up to 2 years of treatment; if the investigator judges that the subject is still benefiting after 2 years, continuation of treatment will be allowed with the consent of the Sponsor), end of treatment follow-up (within 7

days after the decision to leave the group), 30-day safety follow-up, 90-day safety follow-up and long-term follow-up.

The clinical and laboratory assessment are detailed in Table 1 and 2, as well as Section 7.

Each subject will be asked for consent to participate in the non-mandatory biomarker assessment. Subjects who sign the consent (in either monotherapy or combination therapy) will be allocated to two groups. The first group of subjects will undergo fresh tumor biopsy at baseline and at week 2 after the first dose of KN046, while the second group of subjects will undergo fresh tumor biopsy at baseline and at week 4 after the first dose of KN046, for the analysis of changes in tumor-infiltrating lymphocyte (TIL) before and after treatment (Section 4.1, 7.3.6.1).

4.2 Definition of end of study

The end of study is defined as occurring on the date of 1 year after the last dose for all subjects, lost to follow-up, withdrew consent, death, early withdrew from the trial, completed the follow-up visit, or the sponsor assesses that the study has met expectations (such as completion of the primary endpoint evaluation indicator, section 3.2.1), whichever occurs first.

Note: if there are still patients in treatment when the sponsor assesses the study meets the expected results and ends the study, the sponsor will provide a roll-over study protocol to continue providing KN046 and safety follow-up for the patients.

4.3 Premature termination of the study

The whole study may be discontinued prematurely in the event of any of the following:

- New information leading to unfavorable risk-benefit judgement of the study drug.
- Sponsor’s decision that continuation of the study is not justifiable for medical or ethical reasons.
- Poor enrolment of subjects making completion of the study within an acceptable time frame unlikely.
- Discontinuation of development of the Sponsor’s study drug or discontinuation of the development of the defined indication.

If the study is terminated prematurely, the subject should receive a visit as soon as possible and complete the visit as required by Section 7.2. For the sake of protecting the subjects, the investigator may be instructed to conduct additional visits.

After termination of the study, the regulatory authorities and the Independent Ethics Committee (IEC)/Institutional Review Board (IRB) will be notified in accordance with applicable regulations.

Regulatory authorities may also request suspension or termination of the whole study.

4.4 Interim analysis

For KN046 monotherapy cohort, an interim analysis will be performed at the time when the 30 subjects' enrollment in a certain dose end, and at least one post-baseline oncology imaging evaluation completes in the study. Based on the results of the interim efficacy, safety, pharmacokinetics, and biomarker analysis, SMC will discuss whether to further expand in the original cohort or in the enriched population of biomarker-positive individuals or explore higher dose levels and/or different dosing intervals (Figure 1).

For KN046 combination therapy cohort, an interim analysis will be performed at the time when the 25 subjects' enrollment in a certain dose end, and at least one post-baseline oncology imaging evaluation completes in the study. Based on the results of the interim efficacy, safety, pharmacokinetics, and biomarker analysis, SMC will discuss whether to further expand in the original cohort or in the enriched population of biomarker-positive individuals or explore higher dose levels and/or different dosing intervals (Figure 2).

4.5 Scientific Monitoring Committee (SMC)

To ensure the safety and risk-benefit ratio of subjects throughout the study, a SMC will be established to periodically review safety data and clinical efficacy. The SMC includes fixed members from the Sponsor (including, but not limited to Medical Director and Biostatistics Expert), participating investigators (if applicable), and external experts (if applicable).

The SMC will evaluate safety and efficacy data, and make an advice on the initiation, discontinuation, and continuation of enrollment in a cohort, but the Sponsor has final decision priority. The SMC will continuously monitor all safety and efficacy information of subjects (frequency of monitoring will be defined in the SMC charter), make a decision on continuation, modification, and discontinuation of the entire study or a treatment cohort, and make an advice on the addition of subsequent cohorts. The SMC may adjust the frequency of meetings as appropriate during the course of the study.

Specific working procedures are described in the SMC charter, which will be established prior to study enrollment.

5 SELECTION OF TRIAL POPULATION

Only persons meeting all inclusion criteria and no exclusion criteria may be enrolled in this study. Prior to performing any study assessments that are not part of subject's routine medical care, the investigator will ensure that the subject or the subject's legal representative has provided written informed consent.

5.1 Inclusion criteria

I01. Signed informed consent form.

I02. Male or female, 18 years of age or older; willing and able to complete all required procedures of study.

I03. Histology confirmed locally advanced unresectable or metastatic triple-negative breast cancer (TNBC):

- HER2-negative defined as (Wolff et al, 2018) [9].
- HER2 immunohistochemistry (IHC) 0 or 1+; or
- IHC 2+ and in situ hybridization (ISH) not amplified.
- ER-negative and PR-negative defined as IHC < 1%.

I04. Subjects' prior therapy needs to meet the requirement:

- KN046 monotherapy cohort: refractory to or relapsed after at least one prior therapeutic regimen for advanced/metastatic TNBC, prior exposure to a taxane either in localized or advanced/metastatic setting or neoadjuvant/adjuvant chemotherapy setting; subjects who refractory to or relapsed within 12 months after completion of neoadjuvant/adjuvant chemotherapy are considered as first-line treatment failure;
- KN046 plus nab-paclitaxel combination therapy cohort: systemic treatment naïve for advanced/metastatic TNBC; prior radiation therapy or endocrine therapy were allowed; subjects who completed neoadjuvant/adjuvant chemotherapy at least 12 months prior to the first dose of KN046.

I05. Baseline measurable disease according to RECIST 1.1 as determined by the local site investigator/radiology assessment. Target lesions situated in a previously irradiated area are considered measurable if progression has been demonstrated in such lesions.

I06. Have provided recently (within 2-years) or newly obtained core or excisional biopsy from a locally recurrent inoperable or metastatic tumor lesion for central determination of biomarker.

I07. ECOG performance status of 0 or 1 (Appendix 4).

I08. Adequate organ function assessed within 7 days prior to first trial treatment:

- Hematological function
  - ANC≥1.5 x 10^9/L.
  - Hemoglobin≥9 g/dL.
  - Platelets≥100 x 10^9/L.
- Renal function
  - Calculated creatinine clearance≥60 mL/min (Cockcroft-Gault method).
- Hepatic function
  - Total bilirubin≤1.5 x ULN (or 2.5 x ULN for documented Gilberts' syndrome).
  - ALT/AST≤3.0 x ULN (or 5.0 x ULN for documented liver metastasis).
- INR or aPTT ≤1.5 x ULN.

I09. Have a life expectancy of at least 3 months.

I10. If female of childbearing potential, have a negative serum pregnancy test within 7 days prior to first trial treatment.

I11. If female of childbearing potential or a male subject with a partner with childbearing potential, be willing to use a highly effective method of contraception (with a failure rate of less than 1.0% per year) from first study treatment to 24 weeks after completion of the trial treatment.

5.2 Exclusion criteria

E01. Leptomeningeal metastasis or untreated active CNS metastasis or leptomeningeal metastasis. Subjects with CNS metastasis may be eligible provided they are treated and clinically stable for at least 4 weeks and have no evidence of new or enlarging brain metastases and are off steroids 7 days for treating brain metastasis prior to first trial treatment.

E02. Untreated spinal compression fractures: treated spinal compression fractures require a minimum of 2 weeks of disease stability prior to the first dose.

E03. Uncontrolled hypercalcemia (corrected serum calcium concentration > 12 mg / dL or Ca^{2+} concentration >1.5 mmol/L), or symptomatic hypercalcemia requires ongoing bisphosphonate therapy.

E04. Lactate dehydrogenase (LDH) > 2 x ULN.

E05. Uncontrolled cancer pain, analgesic drugs were not at a stable dose before enrollment.

E06. Is currently participating and receiving an investigational drug or has participated in a study of an investigational drug within 28 days.

E07. Has received other anti-tumor treatment within 28 days or within 5 times of half-life (no less than 2 weeks), whichever is shorter prior to the first trial treatment.

E08. Major surgery for any reason, except diagnostic biopsy, within 28 days of the first administration of trial treatment.

E09. Curative radiation within 3 months of the first dose of trial treatment. Radiation to more than 30% of the bone marrow or with a wide field of radiation should not be used within 2 weeks prior to the first administration of trial treatment.

E10. Prior therapy with any antibody/drug targeting T-cell coregulatory proteins (immune checkpoints) such as PD-1, PD-L1, cytotoxic T-lymphocyte antigen-4 (CTLA-4), LAG-3, or curative cancer vaccine.

E11. Subjects with a condition requiring systemic treatment with either corticosteroids (> 10 mg daily prednisone equivalents) or other immunosuppressive medications within 14 days of study drug administration. Inhaled or topical steroids, and adrenal replacement doses ≤ 10 mg daily prednisone equivalents are permitted in the absence of active autoimmune disease. A brief course of corticosteroids for the prophylaxis (e.g., contrast dye allergy) or treatment of non-autoimmune conditions (e.g., delayed-type hypersensitivity reaction caused by contact allergen) is permitted.

E12. Vaccination within 28 days of the first administration of trial treatment, including live attenuated vaccine.

E13. Has interstitial lung disease, or a history of pneumonitis that required oral or intravenous glucocorticoids to assist with management.

E14. History or current active autoimmune disease that might deteriorate when receiving an immunostimulatory agent, including but not limited to:

Myasthenia gravis (MG), Good syndromes, ISAACS syndromes, polymyositis, myocarditis, neuromuscular syndrome (myotonic dystrophy myositis, Eaton-Lambert syndrome), blood disorders (red cell aplasia, hypogammaglobulinemia, T-cell deficiency syndrome, erythrocytosis, pancytopenia, megakaryocytopenia, T-cell lymphocytosis, pernicious anemia), systemic lupus erythematosus, sarcoidosis, scleroderma, Crohn’s disease, inflammatory bowel disease, Wegener syndrome (granulomatosis with polyangitis, Grave’s disease, rheumatoid arthritis, hypophysitis, uveitis), autoimmune hepatitis, systemic sclerosis (for example scleroderma), Hashimoto thyroiditis (with the exception as stated below), hyperparathyroidism, stiff-person syndrome, Addison disease, panhypopituitarism, autoimmune vasculitis, autoimmune neuropathy (Guillain-Barre syndrome) etc.

Subjects with Type I diabetes, vitiligo, psoriasis, hypo- or hyperthyroid disease, Sjögren syndrome not requiring immunosuppressive treatment are eligible. Subjects requiring

hormone replacement with corticosteroids are eligible if the steroids are administered only for the purpose of hormonal replacement and at doses ≤10 mg or equivalent prednisone per day. Administration of steroids for other conditions through a route known to result in a minimal systemic exposure (topical, intranasal, intra-ocular, or inhalation) are acceptable.

E15. Previous malignant disease other than the target malignancy to be investigated in this study except for adequately treated non-melanomatous cancers of the skin, in situ carcinoma of the prostate/cervical/breast cancer, NMIBC-Tis or other malignancy treated at least 5 years previously with surgery and/or curative radiotherapy, and there is no evidence of recurrence since that time.

E16. History of uncontrolled intercurrent illness including but not limited to:

- Active HBV or HCV infection.
- If HBsAg and HCV antibody positive, HBV DNA and HCV RNA assay should be performed. Subjects maybe eligible if HBV DNA ≤ 500 UI/ml (or 2000 copies/ml) or HCV RNA negative.
- Known HIV infection or known history of acquired immune deficiency syndrome (AIDS);
- Active tuberculosis infection.
- Active infection within 4 weeks prior to the first dose of trial treatment that require the use of systemic antibiotics ≥ 7 days.
- Hypertension uncontrolled by standard therapies (not stabilized to 150/90 mmHg).
- Clinically significant (that is, active) cardiovascular disease: cerebral vascular accident/stroke (< 6 months prior to enrolment), myocardial infarction (< 6 months prior to enrolment), unstable angina pectoris, congestive heart failure (New York Heart Association Classification Class II-IV) or serious cardiac arrhythmia requiring medication (including corrected QT interval prolongation of > 470 msec calculated according to Fridericia and/or pacemaker or prior diagnosis of congenital long QT syndrome.

E17. Persisting toxicity related to prior therapy (including any prior investigational therapy) of CTCAE ≥ grade 2 (NCI-CTCAE v5.0) or related toxicity not recovery to baseline, with the exception of alopecia of any grade.

E18. Prior allo-HSCT or solid organ transplant.

E19. Known severe hypersensitivity reactions to antibody drug (≥ grade 3 NCI-CTCAE v5.0), any history of anaphylaxis, uncontrolled asthma (that is, 3 or more features of partially controlled asthma), or any history of severe drug hypersensitivity (for example immune mediated liver toxicity, immune mediated thrombocytopenia or anemia).

E20. Is pregnant or breastfeeding.

E21. Other medical conditions that at the discretion of investigator interfere with the requirements of the trial in terms of safety or efficacy evaluation, or treatment compliance. These include but are not limited to psychiatric or substance abuse disorder, moderate to large pleural fluid/cardiac effusion/ascites, or recurrent/refractory pleural fluid/cardiac effusion/ascites..

5.3 Criteria for subject withdrawal

5.3.1 Discontinuation of Study Drugs

A subject must be discontinuation of KN046 or KN046 plus nab-paclitaxel in the event of any of the following:

- Confirmed PD per RECIST 1.1 (note: If the subject's ECOG performance status remains stable and the investigator determines that the subject will benefit from continuous treatment with KN046, treatment with KN046 will be allowed after first PD judged per RECIST V1.1, as detailed in Section 6.1.3).

- Significant clinical deterioration (clinical progression) is defined as new symptoms that are deemed by the investigator to be clinically significant or significant worsening of existing symptoms (if PD is not met per RECIST 1.1, the subject needs to continue tumor assessment until RECIST 1.1 defined PD is confirmed).

- Therapeutic failure requiring urgent additional anticancer drug.

- Unacceptable toxicity (Section 6.1.4, if a subject does not occur RECIST 1.1 defined confirmed PD, the subject will be asked to continue tumor assessment until RECIST 1.1 defined PD is confirmed).

- Occurrence of pregnancy.

- Use of a nonpermitted concomitant drug as defined in Section 6.3.2 where the predefined consequence is withdrawal from KN046.

- Withdrawal of the subject’s consent to continue KN046 (if a subject withdraws consent, the subject will be asked to continue tumor assessments if RECIST 1.1 defined confirmed PD does not occur).

- Noncompliance.

5.3.2 Withdrawal from the study

Subjects are free to withdraw from the study at any time without giving reasons. In case of withdrawal from the study, the assessment schedule for end-of-treatment visit (EOT visit) should be performed (Section 7.2) if possible and focus on the most relevant assessments. In all cases, the eCRF page records for the EOT visit must be completed whenever possible. Subjects will be asked to continue safety and long-term follow-up, which includes the collection of data on survival and subsequent anticancer therapy.

A subject must be withdrawn in the event of any of the following:

- Withdrawal of the subject's consent. If the subject withdraws consent, it must be clearly stated if the subject is also withdrawing their consent from the post-treatment follow-up assessments.
- Participation in any other therapeutic study during the Treatment Phase of this study; however, subjects will continue to be followed for new anticancer therapy and for survival.
- Lost to follow-up.

If the data are not further collected because the subject completely withdraws from the study or does not return to the visit, the investigator must determine the primary reason for the subject's withdrawal as completely and accurately as possible and record this information in the eCRF page. For a subject who is "lost to follow-up", the investigator should show "due diligence" by documenting in the source documents the steps taken to contact the subject, e.g., dates of telephone calls, registered letters, etc. If permitted by local laws, public records may be assessed to determine vital status information (alive / dead) for a subject who is lost to follow-up.

6 INVESTIGATIONAL MEDICINAL PRODUCT AND TRIAL TREATMENT

KN046 and nab-paclitaxel are the investigational medicinal product of this study.

6.1 Study drug (KN046)

6.1.1 Dosage form and strength

The dosage form of KN046 is IV injection for single use, with a strength of 40 mg/1.6 mL/vial or 300 mg/12 mL/vial.

6.1.2 Drug preparation

Subjects in this trial will receive IV infusion of KN046 over at least 90 minutes (90~120 minutes) once every 2 weeks.

The volume of KN046 solution will be calculated based on the body weight of the subject determined the day prior to, or on the day of each drug administration. For a 70 kg subject who is enrolled and planned to receive 3 mg/kg treatment, the dose of KN046 is 210 mg, and the volume of solution is 210 mg/40 mg x 1.6 mL = 8.4 mL. The dilution needs to be done aseptically by transferring 8.4 mL of 40 mg/ 1.6 mL KN046 from 6 vials into 250 mL of sterile 5% dextrose in water. The diluted KN046 solution should be mixed gently and thoroughly before application by intravenous infusion, with an adequate sterile 5% dextrose flush at the end of the infusion.

Visible foreign matters and color should be examined macroscopically before drug infusion. KN046 cannot be administered as an IV bolus and short infusion.

The actual dose allowed a maximum error of not exceeding ±10% of the theoretical dose.

For the detailed process of the dilution and infusion, please refer to the Pharmacy Manual.

6.1.3 Dose and method of administration of KN046

Subjects in this trial will receive KN046 at the dosage of 3 mg/kg or 5 mg/kg. The rationale for dose selection please refer to section 2.1. When KN046 and nab-paclitaxel are dosed on the same day, nab-paclitaxel should be dosed at least 2 hours after the completion of KN046 infusion.

The body weight of subjects will be measured on the day of dosing or 1 day before dosing to calculate the dose of KN046. Every subject will receive KN046 at protocol scheduled dose and regimen until confirmed disease progression per RECIST 1.1 criteria, significant clinical deterioration (clinical progression), unacceptable toxicity (section 6.1.4), starts new anti-cancer therapy, withdrawal of informed consent, or until they meet any of the protocol-defined criteria for withdrawal from KN046 or the study (section 5.3.1 and 5.3.2). Refer to Section 6.1.4 and Appendix 2 for the conditions with a need to adjust the mode of administration of KN046 (e.g., change in infusion rate, dose delay, resumption of dosing). For the subject who is still deriving benefit from

KN046 after 2 years treatment judged by the investigator, it is allowed to continue treatment with the consent of the sponsor.

After initial determination of disease progression per RECIST 1.1, a subsequent radiographic assessment should be performed preferably 4~8 weeks (but not later) apart after original progressive disease (PD) to determine whether there has been a decrease in the tumor size, or continued PD (Confirmed Progression). Please refer to Section 7.3.2.3.3 for the procedures to determine Confirmed Progression. Before progression is confirmed, the subject may continue treatment with KN046 if the investigator considers potential clinical benefit to the subject and if he/she meets below criteria ("Criteria for Continuation of Treatment Beyond Progression"):

- No decline in ECOG performance status that can be attributed to disease progression.
- No unacceptable toxicity that is related to KN046.
- Absence of fast disease progression that needs a salvage therapy; AND
- Absence of fast disease progression leading to medical emergencies that cannot be managed by protocol-allowed medical interventions (for example, CNS metastasis, spinal compression fracture, or leptomeningeal spread etc).

The decision to continue treatment should be discussed with the Medical Monitor and documented in the study records. If the disease progression is confirmed per RECIST 1.1 criteria and the investigator considers continuing KN046 will benefit the subject, the subject might continue KN046 treatment provided she/he still meets the "Criteria for Continuation of Treatment Beyond Progression". Under such case, subject must provide written consent to acknowledge deferring alternative treatment options in favor of continuing study treatment.

For subjects who continue KN046 beyond RECIST 1.1 defined progression, iRECIST criteria could be referenced for subject care [19]. Treatment of KN046 beyond progression should be stopped immediately if the subject does not tolerate KN046 or if therapeutic failure occurs.

6.1.4 Dose modifications

KN046 will be withheld for ≥ Grade 3 drug-related toxicity, including laboratory abnormalities with the exception of laboratory abnormalities that are not clinically significant or not meet the criteria of adverse event, and severe or life-threatening AEs.

If a dose of KN046 is withheld for toxicity, subjects may resume dosing with KN046 when toxicity has improved to ≤ Grade 1.

In case toxicity does not resolve to Grade 0-1 within 12 weeks after last infusion, trial treatment should be discontinued after discussion with Sponsor Medical Monitor. With the agreement between investigator and Sponsor Medical Monitor, subjects with a laboratory adverse event at Grade 2 after 12 weeks may continue treatment only if asymptomatic and controlled, for example, hypothyroidism or Type I diabetes controlled by substitutional treatments.

Subjects who require corticosteroids to manage drug-related adverse events must be at an equivalent dose of ≤ 10 mg per day of prednisone to resume dosing with KN046. In case of an inability to reduce the corticosteroid dose for managing a drug-related adverse event to the equivalent of ≤ 10 mg prednisone per day within 12 weeks of last KN046 dose, a prompt discussion and agreement between investigator and the Sponsor Medical Monitor regarding the subject's continuation on KN046 treatment.

For a subject who experience a recurrence of the same serious adverse event at the same grade or greater with rechallenge of KN046, the subject must discontinue study medication.

In addition, if any of below drug-related adverse event occurs, it requires permanent discontinuation of KN046 treatment:

- ≥ Grade 3 immune related pneumonitis.
- Recurrence of ≥ Grade 2 immune related pneumonitis lasting ≥ 4 weeks after appropriate medical interventions.
- ≥ Grade 2 immune related CNS toxicities lasting ≥ 4 weeks after appropriate medical interventions.
- ≥ Grade 3 immune related colitis.
- ≥ Grade 3 uveitis or optic neuritis.
- ≥ Grade 3 immune related hepatitis with ALT/AST > 8 x ULN OR total bilirubin> 3 x ULN.
- ≥ Grade 3 immune related nephritis or renal dysfunction.

Table 10 Guideline on KN046 dose modification

Toxicity	Grade	Withheld treatment	Resume treatment	Dose / schedule for resuming treatment	Discontinuation of treatment
Hematological toxicity	1, 2	No	Not applicable	Not applicable	Not applicable
	3 (except for isolate event of grade 3 neutropenia)	Yes	Toxicity resolves to ⩽ Grade 1 or baseline within 12 weeks of last KN046 dose	Resume at planned dose and may increase the dose interval by 1 week at each occurrence	Permanent discontinuation if toxicity does not resolve to ⩽ Grade 1 or baseline within 12 weeks of last KN046 infusion
	4	Yes			
	1	No	Not applicable	Not applicable	Not applicable
	2	Yes	Toxicity resolves to ⩽ Grade 1 or baseline within 4 weeks of last KN046 dose	Resume at planned dose and dose interval	

Toxicity	Grade	Withheld treatment	Resume treatment	Dose / schedule for resuming treatment	Discontinuation of treatment
Non-hematological toxicity with the exception for alopecia of any Grade and Grade 2 fatigue which will be treated as Grade 1 adverse reaction			Toxicity resolves to ⩽ Grade 1 or baseline beyond 4 weeks and within 12 weeks of last KN046 dose	Resume at planned dose and may increase the dose interval by 1 week at each occurrence	Permanent discontinuation if toxicity does not resolve to ⩽ Grade 1 or baseline within 12 weeks of last KN046 infusion
	3, 4	Yes	Toxicity resolves to ≤ Grade 1 or baseline within 12 weeks of last KN046 dose	Resume at planned dose and may increase the dose interval by 1 week at each occurrence	Permanent discontinuation if toxicity does not resolve to ≤Grade 1 or baseline within 12 weeks of last KN046 infusion; Permanent discontinuation should also be considered for any life-threatening or severe adverse reactions
**Note: if increased Grade 3 or 4 CPK occurs, myocardial enzymes (CPK, CK-MB, TnI, TnT) should also be measured. KN046 will be permanently discontinued in case of elevation on treatment-related myocardial enzymes**					

Infusion related reactions and irAEs should be handled according to the guidelines provided in Sections 6.3.3.1 and appendix 2, respectively.

As a biological product, KN046 retains ADCC activity. Refer to Section 6.3.3.2 for the management of potential tumor lysis syndrome.

If KN046 is interrupted due to adverse events, the investigator should decide whether to continue using nab-paclitaxel based on clinical practice.

6.1.5 Treatment Assignment

The subject will be given a subject number after signing the ICF. The number will also be used as a treatment assignment number when the subject receives KN046. Each of subject numbers and treatment assignment numbers could not be reassigned to other subjects once it is determined.

6.1.6 Drug Packaging and Labeling

KN046 is aseptically filled in a neutral borosilicate colorless glass vial, and sealed with a halobutyl rubber stopper and an aluminum-plastic cap. Each 2 mL vial contains 1.6 mL of KN046 drug solution. Each vial is packed in cardboard boxes.

The vial label mainly contains the following information: protocol number, drug content and strength, batch number, expiry date, storage conditions, and usage. The contents of the label will meet the requirements of current regulations.

6.1.7 Storage and handling requirements

Study drugs should be dispensed to the study site only after receipt of the requisition form in accordance with relevant regulations and the provisions of the sponsor. Drug use should be performed according to the following procedures. KN046 may not be given until subjects have been enrolled in this study. Only authorized site personnel can supply or manage study drugs. In order to ensure the safety of the subjects and to infuse the drugs according to the regimens, the subjects must return to the study site for each dose and should not carry and use the drugs themselves. The study drugs must be stored in a secure area that is accessible only by the investigator and authorized study site personnel and meets the storage requirements of the study drugs at 2-8℃.

6.1.8 Drug quantity management

The investigator is responsible for the quantity verification, dispensing and record maintenance of the study drugs. In accordance with relevant regulatory requirements, the investigator or site designee must maintain a drug accountability record throughout the study. It includes the quantity of study drugs received from the sponsor and the quantity supplied to the subjects. After completion of the study, all remaining vials and unused KN046 drugs will be registered and destroyed on site or at an independent center with written consent from the sponsor.

6.1.9 Assessment of investigational medicinal product compliance

Clinical research associates will verify compliance against body weight, group, and other medical records.

6.1.10 Occupational Safety

KN046 does not pose an occupational safety risk to site staff under normal compounding and dosing conditions.

6.2 Study drug (Nab-paclitaxel)

Nab-paclitaxel will be centrally supplied by the sponsor. Detailed specifications and preparation methods for nab-paclitaxel can be found in the provided instructions.

6.2.1 Dose and method of administration of nab-paclitaxel

Subjects in this trial will receive nab-paclitaxel at dose of 100 mg/m^2 administered as an intravenous infusion on Days 1, 8, and 15 of each 28-day cycle. The subject is expected to receive 6 cycles of nab-paclitaxel. The infusion time of each cycle is about 30 minutes. After 6 cycles treatment, if the subject has no disease progression or intolerable toxicity, the investigator can decide to continue the nab-paclitaxel based on clinical practice and the benefit-risk assessment.

The dose calculation of nab-paclitaxel is according to the following steps:

- Calculate body surface area (BSA)
    - Measure the height (cm) and weight (kg) of the subject.
- Calculate the BSA according to the Dubios formula:
    - Dubios: BSA (m2) = 0.007184 x (height) 0.725 x (weight) 0.425.
- Calculate the dosage following the formula:
    - Nab-paclitaxel dose = BSA x 100 (mg).

6.2.2 Dose modification of nab-paclitaxel

Patients who experience severe hematologic or non- hematologic toxicities during nab-paclitaxel therapy should have dose modification for subsequent courses. Dose modification guidelines are summarized below as Table 11. Nab-paclitaxel is allowed for dose reduction up to 2 times. If a third dose reduction is needed, treatment should be permanently terminated.

If nab-paclitaxel is discontinued due to adverse events, the investigator should refer to sections 6.1.3 and 6.1.4 to determine whether to continue KN046 treatment.

Table 11 Guideline on nab-paclitaxel dose reduction

	Original plan Dose	Nab-paclitaxel
Dose Level - 0	100%	100 mg/m^2
Dose Level - 1	75%	75 mg/m^2
Dose Level - 2	50%	50 mg/m^2
Dose Level - 3	--	Discontinue permanently

6.2.2.1 Dose modification for hematologic toxicities of nab-paclitaxel

Do not administer nab-paclitaxel on day 1 of a cycle until absolute neutrophil count (ANC) is at least 1500 cells/mm^3 and platelet count is at least 100,000 cells/mm^3. Do not administer nab-paclitaxel on day 8 or 15 of a cycle until ANC is at least 500 cells/mm^3 and platelet count is at least 50,000 cells/mm^3.

Table 12 Guideline of nab-paclitaxel modification on hematologic toxicities

Adverse Reaction	Nab-paclitaxel (day 1 of every cycle)	
	Occurrence	Nab-paclitaxel (mg/m^2)
Neutropenic Fever (no matter how long it lasts) OR Delay of next cycle by more than 7 days for ANC less than 1500/mm^3 OR ANC less than 500/mm^3 for more than 7 days	First	75
	Second	50
	Third	Discontinue permanently
Platelet count less than 50,000/mm^3	First	75
	Second	Discontinue permanently

6.2.2.2 Dose modification for non-hematologic toxicities of nab-paclitaxel

It is necessary to obtain laboratory test results before each administration of nab-paclitaxel (Table 2). Table 13 summarizes the recommended dose modification for nab-paclitaxel in the event of non-hematologic toxicities. If there is other event not listed, please refer to the instructions of nab-paclitaxel to implement dose modification.

Table 13 Guideline of nab-paclitaxel modification on hematologic toxicities

Adverse Reactions		Nab-paclitaxel modification
**Hepatic Impairment**	AST < 10 x ULN or TBIL 1 ~ < 1.5 x ULN	100
	AST < 10 x ULN or TBIL 1.5 ~ < 2.5 x ULN	Withhold until AST<10 x ULN and TBIL 1~ < 1.5 x ULN, reduce dose to 75 mg/m^2;<br>Discontinue permanently if it cannot improve to AST<10 x ULN and TBIL 1~ < 1.5 x ULN within 3 weeks.
	AST<10 x ULN or TBIL 2.5 ~ < 5 x ULN	Withhold until AST<10 x ULN and TBIL 1~ < 1.5 x ULN, reduce dose to 50 mg/m^2;<br>Discontinue permanently if it cannot improve to AST<10 x ULN and TBIL 1~ < 1.5 x ULN within 3 weeks.
	AST⩾10 x ULN or TBIL ⩾ 5 x ULN	Discontinue permanently
**Neurotoxicity**	Grade 3 or 4 peripheral neuropathy for 1st time	Withhold until improves to ⩽ Grade 1, resume at 75 mg/m^2
	Grade 3 or 4 peripheral neuropathy for 2nd time	Withhold until improves to ⩽ Grade 1, resume at 50 mg/m^2
	Grade 3 or 4 peripheral neuropathy for 3rd time	Discontinue permanently
**hypersensitivity**	Grade 3 or 4	Discontinue permanently

6.3 Concurrent medications and therapies

6.3.1 Permitted medications and procedures

Any medications considered necessary for subjects' welfare and will not interfere with KN046 may be given at the investigator's discretion. The investigator will record all concomitant medications taken by the subject from 28 days prior to first trial treatment, during the study till 90 days after last KN046 dose.

Palliative radiotherapy to bone (e.g., local radiotherapy to relieve bone pain, or to prevent the risk of fracture due to lytic lesions) is allowed in this study, but it is required that lesions in the area where palliative radiotherapy to bone are not selected as target lesions and palliative radiotherapy is not intended to treat tumors. PD should be judged based on RECIST 1.1 criteria rather than the need for palliative radiotherapy to bone.

6.3.2 Prohibited medications and procedures

The following treatments must not be administered during the study:

- Anticancer treatment other than the investigational drug (for example, cytoreductive therapy, radiotherapy with the exception of palliative radiotherapy or radiotherapy administered to superficial lesions, immunotherapies, or cytokine therapy except for erythropoietin).

- Concurrent systemic therapy with steroids or other immunosuppressive agents with the exception of the treatment of irAEs, prophylactic use to prevent allergic reactions to the contrast for imaging evaluation, for the prevention of acute infusion-related reactions, or administration of steroids for hormone replacement at doses ≤10 mg or equivalent of prednisone per day. Steroids with no or minimal systemic effect (topical, intranasal, intra-ocular, or inhalation) are allowed.

- Immunosuppressive drugs with the exception for the treatment of irAEs.

- Traditional Chinese medicine that has an approved indication in treating cancer from National Medical Products Administration (NMPA).

- Other investigational drug.

- Major surgery excluding diagnostic biopsy.

- RANKL inhibitor (such as denosumab) which is used to treat bone metastases.

- Herbal remedies with immunostimulating properties (eg, mistletoe extract) or known to potentially interfere with major organ function (eg, hypericin).

If the administration of a nonpermitted concomitant drug becomes necessary during the study, the subject will be withdrawn from treatment with KN046. The Sponsor may be contacted to discuss whether KN046 must be discontinued.

6.3.3 Special precautions

As a routine precaution, subjects enrolled in this study must be observed for 2 hours after the completion of infusion of KN046 in an area with resuscitation equipment and emergency agents. At all times during study drug administration, immediate emergency treatment of an infusion-related reaction or a severe hypersensitivity reaction according to institutional standards must be assured, for instance, dexamethasone 10 mg and epinephrine in a 1:1000 dilution or equivalents should always be available along with equipment for assisted ventilation.

Infusion of KN046 will be stopped in case of ≥Grade 3 hypersensitivity or anaphylactic reaction. The treatment recommendations for infusion-related reactions, severe hypersensitivity reactions and tumor lysis syndrome according to the NCI are outlined in Sections 6.3.3.1 and 6.3.3.2, respectively.

Investigators should monitor subjects closely for potential irAE, which may manifest at the earliest after the first dose of treatment. Such events may consist of autoimmune hepatitis, diarrhea and colitis, arthritis, persistent rash, glomerulonephritis, cardiomyopathy or inflammatory eye conditions etc. Details for irAE management please refer to appendix 2.

6.3.3.1 Infusion-related reactions and hypersensitivity reactions

Infusion-related reactions could manifest as fever, chills, rigors, diaphoresis or headache. Hypersensitivity reactions could manifest as impaired airway, decreased oxygen saturation (<93%), confusion, lethargy, hypotension, pale or clammy skin and cyanosis.

Management of infusion-related reactions and hypersensitivity caused by KN046 is provided in below table.

If a hypersensitivity reaction occurs, the subject must be treated according to the best available medical practice. A complete guideline for the emergency treatment of anaphylactic reactions according to the Working Group of the Resuscitation Council (United Kingdom) can be found at https://www.resus.org.uk/pages/reaction.pdf (refer to: Emergency Treatment of Anaphylactic Reactions: Guidelines for Healthcare Providers, 2008). Subjects should be instructed to report any delayed reactions to the Investigator immediately.

If severe immediate systemic hypersensitivity occurs, the subject should be placed on a monitor immediately and receive epinephrine and corticosteroid infusion as appropriate. The intensive care unit should be alerted for possible transfer if required.

If the subject is suspected to have systemic hypersensitivity reaction, the investigator is advised to collect plasma histamine, C-reactive protein (CRP) and the presence of drug-specific immunoglobulin E within 30 minutes of system onset, and urinary methyl histamine within 24 hours of onset of symptoms, as well as unplanned ADA and PK samples. For subjects with chest pain, ECG, myocardial enzymes (CPK, CK-MB, TnI, TnT) and BNP are recommended within 30 minutes of the onset of symptoms.

If investigator determines that the benefit of the continued administration outweighs the risk, the KN046 treatment is allowed to continue, and the prophylactic medical must be administered 30-60 minutes prior to the administration of all subsequent dosing cycles. Examples of prophylactic dosing regimens are diphenhydramine 25~50 mg IV, cimetidine 300 mg IV and acetaminophen 650 mg PO. If the subject's hypersensitivity reaction includes bronchospasm or dyspnea, montelukast 10 mg PO is recommended as part of a prophylactic regimen. Investigators can use medical judgment to determine whether steroids need to be added to a preventive regimen. Encourage discussion with immunologists to determine treatment options.

Table 14 Guideline on management of IRR and hypersensitivity reactions

NCI-CTCAE Grade	Treatment Modification for KN046
**Grade 1 – mild**<br>Mild transient reaction; infusion interruption not indicated; intervention not indicated.	Decrease the KN046 infusion rate by 50% and monitor closely for any worsening.
**Grade 2 – moderate**<br>Therapy or infusion interruption indicated but responds promptly to symptomatic treatment (e.g., antihistamines, NSAIDs, narcotics, i.v. fluids); prophylactic medications indicated for ≤ 24 hours.	Stop KN046 infusion.<br>Resume infusion at 50% of previous rate once infusion-related reaction has resolved or decreased to at least Grade 1 in severity, and monitor closely for any worsening.

NCI-CTCAE Grade	Treatment Modification for KN046
	**Once the KN046 infusion rate has been decreased by 50% due to an infusion-related reaction, it must remain decreased for all subsequent infusions. At next cycle, a premedication regimen of diphenhydramine 25~50 mg IV, cimetidine 300 mg IV and acetaminophen 650 mg po is mandatory 30 to 60 minutes prior to each dose of KN046). If subject developed symptomatic bronchospasm, premedication with montelukast (10 mg PO) is mandatory as part of premedication regimen mentioned above. At the discretion of investigator, premedication of corticosteroid can also be considered. If a subject has a recurrence of ≥grade 2 infusion related reactions despite the premedications as well as decreased infusion rate, the subject should be withdrawn from KN046 treatment.**<br>**It's also suggested to the investigator discuss with the allergist locally.**
**Grade 3 or Grade 4 – severe or life-threatening** Grade 3: Prolonged (e.g., not rapidly responsive to symptomatic medication and/or brief interruption of infusion); recurrence of symptoms following initial improvement; hospitalization indicated for clinical sequelae.<br>Grade 4: Life-threatening consequences; urgent intervention indicated.	Stop the KN046 infusion immediately and disconnect infusion tubing from the subject.<br>Subjects have to be withdrawn permanently from KN046.
Note: once the subject develops infusion related reactions, additional appropriate medical therapy may include but is not limited to: IV fluids, antihistamines, NSAIDS, acetaminophen and narcotics. Increase monitoring of vital signs as medically indicated until the subject is deemed medically stable in the opinion of the investigator. If symptoms resolve within one hour of stopping drug infusion, the infusion may be restarted at 50% of the original infusion rate (e.g. from 100 mL/hr to 50 mL/hr). Otherwise dosing will be held until symptoms resolve and the subject should be premedicated for the next cycle.	

i.v.=intravenous, NCI-CTCAE=National Cancer Institute-Common Terminology Criteria for Adverse Event, NSAIDs=nonsteroidal anti-inflammatory drugs.

6.3.3.2 Tumor lysis syndrome

Human IgG1 Fc segment is retained in the KN046 structure, and *in vitro* experiments have confirmed that KN046 has the ADCC function; after receiving KN046 treatment, the subjects may be at the risk of ADCC-induced tumor lysis syndrome.

Tumor lysis syndrome (TLS) [21] refers to serious metabolic disorder caused by short-term rapid dissolution of tumor cells spontaneously or under the action of anti-tumor drugs, resulting in rapid release of various electrolyte ions, nucleic acids, proteins and other metabolites in cells into the blood, and it is clinically characterized by hyperuricemia, hyperkalemia, hyperphosphatemia, hypocalcemia and acute renal failure.

TLS can be treated as shown in **Figure 3.**

Figure 3 Evaluation and initial treatment of TLS [21]

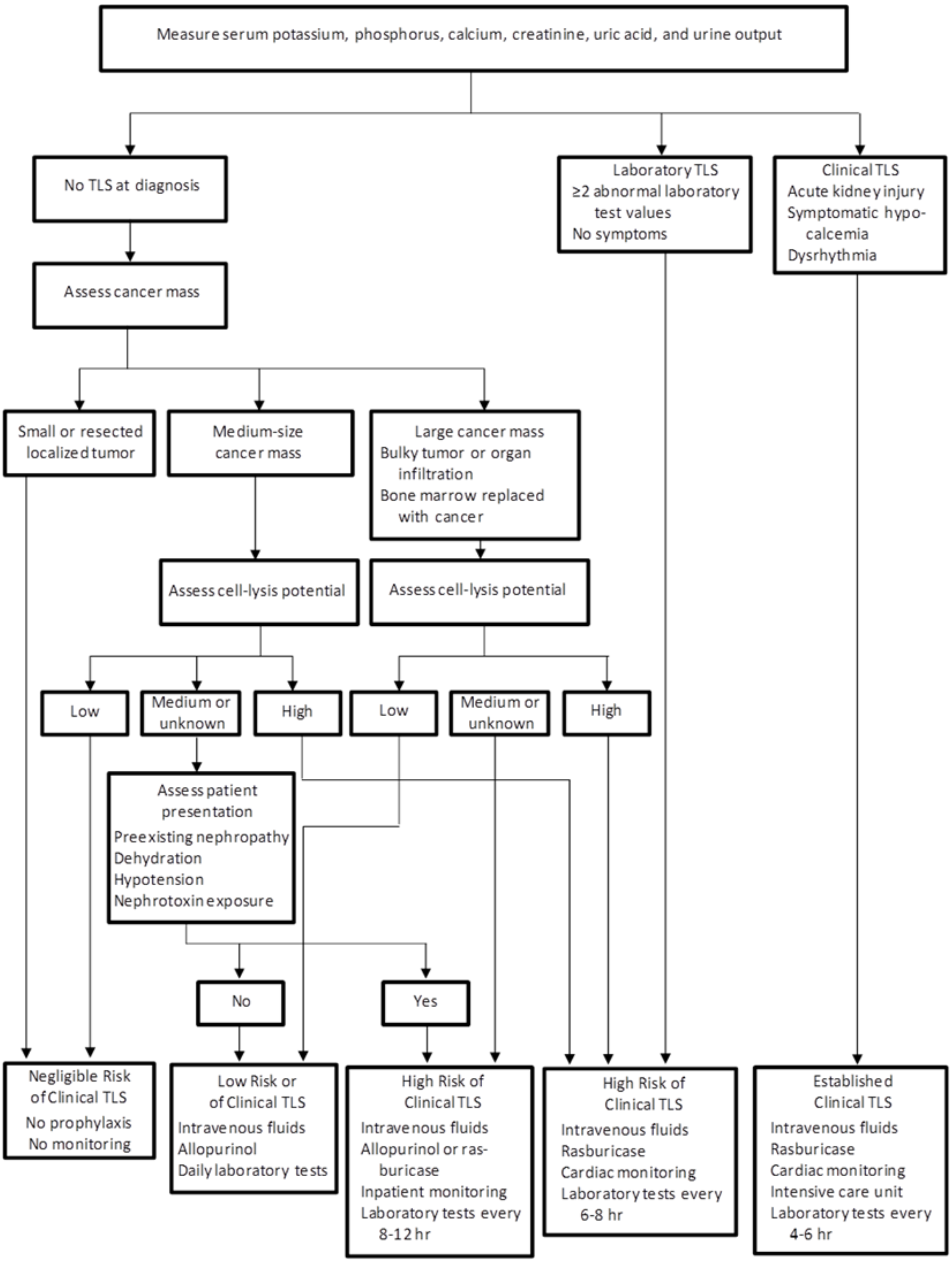

7 PROCEDURES AND ASSESSMENTS

7.1 Schedule of visits plan

A complete Schedule of Assessments is provided in Table 1, Table 2 and Table 3.

7.1.1 Screening and baseline period

Prior to performing any trial assessments not part of the subject's routine medical care, the investigator will ensure that the subject or the subject's legal representative has provided written informed consent.

During the Screening period and before any trial-related investigations and assessments are started, subjects will be asked to sign the ICF. The Screening procedures and Baseline assessments will be completed within 28 days of signing the ICF before first trial treatment.

The subjects' information that will be documented during Screening includes the demographic information (date of birth, sex, race and ethnicity) and the complete medical history (oncology history, previous and ongoing medications, prior surgery, radiation and medication therapies, and baseline medical condition etc.). The AE reporting period for safety surveillance and a concomitant medication recording period begins when the subject first signs an informed consent.

During Screening, subjects will undergo a complete physical examination, vital signs, recording height and body weight, 12-lead ECG, and a determination of the ECOG performance status.

The Screening laboratory examination includes hematology, coagulation, serum chemistry, troponin, C-reactive protein, and urinalysis. Adrenocorticotropic hormone (ACTH), total T3, free T3, free T4 and TSH will also be assessed at Screening for all subjects. Viral tests for HBV, HCV and HIV should be performed at Screening.

During Screening, a serum beta-human chorionic gonadotropin pregnancy test will be performed for females of childbearing potential. Females who are postmenopausal or who have undergone hysterectomy or bilateral oophorectomy are exempt from pregnancy testing. If necessary to confirm postmenopausal status, FSH will be drawn at Screening.

During Screening, the tumor evaluation will be performed using CT scan or MRI or any other established methods. A brain CT/MRI scan is required at Screening if not performed within 42 days prior to first trial treatment. A bone scan should be done at Screening if not be done within 12 weeks prior to first trial treatment.

Failure to establish subject eligibility due to laboratory parameters is allowed to be re-tested following consultation with the Medical Monitor.

Subjects who sign the ICF but do not start trial treatment for any reason will be considered Screening failure. The following eCRF pages must be completed for Screening failure:

- Informed consent;
- Reason of Screening failure;
- Demography;
- Adverse events (only if an SAE occurs);
- Inclusion / exclusion page.

7.1.2 Treatment period

The Treatment period begins on Cycle 1 Day 1 with the first administration of investigational drug (KN046, or KN046 plus nab-paclitaxel) and will continue until confirmed disease progression per RECIST 1.1, significant clinical deterioration (clinical progression), unacceptable toxicity, or any criterion for withdrawal from the investigational medicinal product. A subject may remain on treatment beyond disease progression per RECIST 1.1 if their ECOG performance status remains stable, no new symptoms or worsening of existing symptoms and if in the opinion of the investigator the subject will benefit from continued treatment. In this case, iRECIST [19] criterion may be referenced for subject care. For subject continues KN046 beyond progression, it should be stopped immediately if the subject no longer tolerates KN046 or if therapeutic failure occurs.

Subjects will be asked to visit the study site every 2 weeks during the Treatment Period. A time window of up to 3 days before or after the scheduled visit day (+/-3 days) will be permitted for all study procedures except for body weight, which should be measured on the day prior to, or the day of, administration of KN046 or KN046 plus nab-paclitaxel. In addition, the tumor evaluation has a tumor assessment window of up to 7 days before or after the scheduled visit day (+/- 7 days) throughout the Treatment phase.

The assessments to be performed during the Treatment phase are detailed in Table 1 and

.

7.2 End of treatment

Subjects must undergo an EOT visit after discontinuation of KN046 for any reason. The EOT visit should be performed within 7 days after the decision to discontinue KN046, but before any new anticancer therapy is started, whichever occurs earlier. The EOT visit may be performed on the day of the decision to discontinue KN046 and under such case EOT exams may not be repeated if have been done since last dose of KN046.

The EOT visit eCRF page should be completed with a visit date reflecting the date the discontinuation decision was made, with the last known date the subject received KN046, and 1 of the following reasons:

- Adverse event(s)
- Abnormal laboratory value(s)
- Abnormal test procedure result(s)
- Protocol violation
- Withdrawal of informed consent
- Lost to follow-up
- Death (must state if death is due to "Study Indication" or "Other" reason)
- Disease progression per RECIST 1.1 criteria
- Clinical deteriorations

- New cancer therapy (optional, to be used when follow-up for progression is stopped in this case)
- Other reasons, including administrative problems.

For reasons other than progression (and death) it should be checked if this was not in fact progression (especially reasons Adverse Events, Abnormal laboratory value (s), Abnormal test procedure result and subject withdrew consent). Also, it should be checked if subject withdrew consent because of safety issues, in which case reasons Adverse Events, Abnormal laboratory value (s), Abnormal test procedure result should be used. In such cases where the reason for discontinuation is Adverse Event, the adverse event eCRF page must be consistent with the EOT reason provided.

If RECIST 1.1 defined progressive disease does not occur at the EOT, the subject will be asked to continue tumor assessments for up to 1 year. If a subject withdraws their consent, it must be clearly stated if the subject is also withdrawing their consent from post-treatment follow-up assessments.

Please refer to Table 1 and

for the specific assessments to be performed at the EOT visit.

7.2.1 Safety follow up

The safety parameters to be assessed during the Safety follow-up are detailed in Table 1 and

.

All subjects will have Safety Follow-up visits scheduled 30 days (+/- 7 days) and 90 days (+/- 14 days) after the last administration of KN046.

After the EOT visit, all AEs have to be documented until the 30-day Safety Follow-up visit. All SAEs and all treatment-related nonserious AEs must be documented until the 90-day Safety Follow-up visit.

During Safety Follow-up, subjects will be asked about any anticancer therapy.

7.2.2 Long term follow up

Subjects with an ongoing SAE after 30-/90-day Safety Follow-up visits must be monitored and followed up by the investigator until stabilization or until the outcome is known, unless the subject is documented as “lost to follow-up”. Any SAE assessed as related to trial treatment must be reported whenever it occurs, irrespective of the time elapsed since the last dose of KN046.

After the EOT visit, subjects will be followed every 12 weeks (+/- 14 days) for survival (including the assessment of any further anticancer therapy). Survival follow-up will continue until at least 75% subjects of the study have died. Under some circumstances, subjects may not be followed for survival for 1 year in this study, for example, subjects may be given the opportunity to participate a rollover study, or the Sponsor may terminate the study early.

Each subject will be followed for survival until death, lost to follow-up, or the cut-off date of the End of Study. Subjects without documented progressive disease according to RECIST 1.1 at the EOT visit will be followed-up for tumor assessment. The reason for death should be documented (and will be coded using MedDRA); it should be also stated if death was due to ‘Study indication’ or ‘Other’ reason.

The assessments to be performed at the long-term follow-up visits are detailed in Table 1 and Table 2.

7.3 Study assessments

7.3.1 Demographic and other baseline characteristics

The assessments and procedures described in this section will be performed during Screening period.

7.3.1.1 Demographic data

At screening, the following data will be collected:

- Date of birth
- Sex
- Race
- Ethnicity.

7.3.1.2 Tumor diagnosis

The tumor disease information to be documented and verified at Screening visit for each subject includes:

- Detailed history of the tumor including histopathological diagnosis, grading and staging in accordance with Masaoka-Koga and AJCC staging system.
- All therapy used for prior treatment of the tumor (including surgery, radiotherapy, chemotherapy, targeted therapy, antibody conjugates, tumor vaccines, and immunotherapy).
- Current cancer signs and symptoms, and AEs effects from current and/or previous anticancer treatments.
- Current cancer disease status.

7.3.1.3 Medical history

In order to determine the subject's eligibility to participate in the study, a complete medical history will be collected and documented during Screening visit. This will include but may not be limited to:

- Past and concomitant nonmalignant diseases and treatments;
- Past and concomitant malignant diseases and treatments;
- All medications (including herbal medications) taken and procedures carried out within 28 days prior to Screening;
- Smoking history;
- Family cancer history.

7.3.1.4 Other baseline assessment

Additional baseline evaluations include oncology evaluations, vital signs, full physical examinations, ECOG score, laboratory tests, 12-lead ECG, biomarker tests, and evaluation of inclusion/exclusion criteria.

7.3.2 Efficacy assessments

In this study, the investigator will evaluate tumors according to RECIST 1.1 (Appendix 1). The results assessed by investigators will be used to make clinical decisions (such as discontinuation of KN046) as well as secondary and exploratory endpoint analyses. For detailed tumor assessment, please refer to the imaging manual.

7.3.2.1 Computed Tomography (CT), MRI, FDG PET/CT, and other assessment

For all subjects, tumor response assessment will be performed by CT scan or MRI of the chest / abdomen / pelvis (when MRI is used, CT of chest is mandatory) and other established assessments of tumor burden if CT/MRI imaging is insufficient for the individual subject. CT scan or MRI should preferably be performed using a 5 mm slice thickness with a contiguous reconstruction algorithm.

CT/MRI scan slice thickness should not exceed 8 mm cuts using a contiguous reconstruction algorithm. Chest X-ray or ultrasound should not be used to measure tumor lesions. All the scans performed at baseline and other imaging performed as clinically required (other supporting imaging) need to be repeated at subsequent visits. In general, lesions detected at baseline need to be followed using the same imaging methodology and preferably the same imaging equipment at all subsequent tumor evaluation visits.

A brain CT/MRI scan (either, with contrast preferred) is required at Screening if not performed within the previous 42 days (6 weeks). Thereafter, a brain CT/MRI scan should be performed, if clinically indicated by the development of new specific symptoms. A bone scan should be performed at Screening if not performed within the previous 12 weeks and beyond as clinically indicated.

For each subject, the investigator will designate 1 or more of the following measures of tumor status to follow for determining response or disease progression: CT or MRI images of primary and/or metastatic tumor masses, physical examination findings, and the results of other assessments. All available images collected during the study period will be considered. The most appropriate measures to evaluate the tumor status of a subject should be used. The measures to be chosen for subsequent evaluation during the study must correspond to the measures used to document the progressive tumor status that qualifies the subject for enrolment.

7.3.2.2 Required tumor assessment at baseline

Baseline tumor assessment will be performed within 28 days prior to the first administration of KN046 in order to document the baseline status of the tumor disease, using RECIST 1.1 target and nontarget lesions.

To be eligible for the study, at baseline, subjects must have measurable disease as per RECIST 1.1. Subjects with only non-measurable lesions are not eligible.

Measurable disease is defined as the presence of at least 1 measurable non-nodal or nodal lesion:

Measurable non-nodal lesion: non-nodal lesions that can be accurately measured with at least 1 dimension with minimum lesion size no less than double the slice thickness (i.e., ≥ 10 mm with spiral CT scan or MRI if the slice thickness is 5 mm).

Measurable nodal lesion: lymph nodes ≥ 15 mm in the short axis.

Lytic bone lesions or mixed lytic-blastic lesions with identifiable soft tissue components that can be evaluated by CT/MRI.

Non-measurable lesions are all other lesions that are considered non-measurable, including small lesions (e.g., with the longest diameter < 10 mm with CT scan or MRI if the slice thickness is 5 mm, or pathological lymph nodes with ≥ 10 mm to < 15 mm short axis). Examples are blastic bone lesions, leptomeningeal disease, ascites, pleural/pericardial effusion, inflammatory breast disease, lymphangitis cutis/pulmonis, abdominal masses that are not confirmed and followed by imaging techniques, and cystic lesions.

Any potentially measurable lesion that has previously been treated with radiotherapy should be considered as a non-measurable lesion. However, if a lesion previously treated with radiotherapy has clearly progressed since the radiotherapy, it can be considered as a measurable lesion.

Target lesions are defined as all measurable lesions (nodal and non-nodal) up to a maximum of 5 lesions in total (and a maximum of 2 lesions per organ), representative of all involved organs. Target lesions should be selected on the basis of their size (lesions with the longest diameter) and their suitability for accurate repeated measurements. Each target lesion must be measured, and uniquely and sequentially numbered on the eCRF (even if it resides in the same organ) at baseline.

Nontarget lesions are all other lesions, i.e., those lesions that do not fulfil the criteria for target lesions at baseline. Multiple nontarget lesions involved in the same organ can be assessed as a group and recorded as a single item. Measurement of these lesions is not required. Each nontarget lesion identified at baseline must be documented on the eCRF.

7.3.2.3 Subsequent tumor assessment for determination

Subsequent tumor assessment will be performed every 8 weeks in the first 12 months and every 12 weeks thereafter until confirmed progression according to RECIST 1.1, start of a new anticancer therapy, death, lost to follow-up or subject withdrawal of informed consent. For post baseline tumor assessments, all target / non-target lesions that were present at baseline must be accounted by the same technique as used at baseline and documented on the eCRF. If possible, a single radiologist should perform all tumor assessment for an individual subject. New lesions are those not observed at baseline but that occur later on. The appearance of a new lesion, either measurable or non-measurable should be reported accordingly in the New Lesion eCRF page.

In case of a RECIST 1.1 defined CR or PR, a confirmatory CT or MRI scan must be performed preferably at the scheduled 8-week interval and no sooner than 4 weeks (Confirmation of Response).

If a subject experience a RECIST 1.1 defined PD, PD should be confirmed preferably 4~8 weeks apart and not later after initial progression has been diagnosed, using RECIST 1.1 criteria (Confirmed Progression).

If progression is based on the occurrence of a new lesion in an area that was not scanned at baseline, a further on-study scan 4~8 weeks later should be considered prior to performing the endo-of-treatment visit. If discontinuation occurs due to progression and a definitive diagnosis / radiographic confirmation according to RECIST 1.1 is not made at the time of discontinuation, a second imaging scan may be allowed for confirmation of progression.

A post-treatment tumor biopsy may be performed in the case of imaging-evaluated disease progression in order to differentiate between actual disease progression and a tumor flare resulting from intratumor inflammation. These biopsies are optional. Should the histology of the biopsy performed be consistent with RECIST 1.1 defined tumor progression, treatment with KN046 may continue provided the subject meets the Criteria of Treatment Beyond Progression (see Section **错误!未找到引用源。**). For subjects who continue KN046 beyond RECIST 1.1 defined progression, iRECIST will be referenced for subject care.

The investigator may perform scans in addition to scheduled study scans for medical reasons or if the investigator suspects PD. Subjects who withdraw from treatment for clinical or symptomatic deterioration before objective documentation of PD will be asked to undergo appropriate imaging to confirm PD.

7.3.3 Safety assessments

The safety profile of every subject will be assessed through the recording, reporting and analyzing of baseline medical conditions, AEs, physical examinations, vital signs, and laboratory tests. Comprehensive assessment of any apparent toxicity experienced by the subject will be performed throughout the course of the study, from the time of the subject’s signature of informed consent.

The AE reporting is described in Section 8.

7.3.3.1 Vital signs

Vital signs include respiratory rate, heart rate, blood pressure and body temperature and will be performed in below visits:

- Screening

- Pre-dose of Day 1 of each KN046 and/or nab- paclitaxel cycle:

    - In the D1 of 1st to 4th doses of KN046, they will be measured before the infusion (-60 min), 30 minutes (± 10 min) after the infusion, 15 minutes after the end of infusion, and 2 hours (+ 30 min) after the end of infusion.

- End-of-Treatment visit

- 30- and 90-day safety follow-up.

When vital signs are scheduled on the same day of safety laboratory tests and pharmacokinetics assessment, vital signs should be performed first.

7.3.3.2 Physical examinations

Full physical examination will include an examination of general appearance, skin, neck (including thyroid), eyes, ears, nose, throat, lungs, heart, abdomen, back, lymph nodes, extremities, and a basic nerve system evaluation.

Full physical examination will be performed in below visits:

- Screening

- End-of-treatment visit.

Symptom-directed physical examination will include general status and any new or worsening of abnormal findings. Symptom-directed exam will be performed in below visits:

- Pre-dose of Day 1 of each KN046 and/or nab- paclitaxel cycle;

- 30- and 90-day safety follow-up.

Results from the physical examination including any abnormalities will be documented in the eCRF in either prior/current medical history or AE page. Abnormal findings are to be reassessed at subsequent visits. Details of the physical examination must be present in the source documentation at the study site.

7.3.3.3 Height and body weight

Body weight (to the nearest 0.1 kilogram [kg]) will be measured at Screening and at subsequent visits as indicated in the Schedule of Assessments (Table 1) and documented in the eCRF. Body height in centimeters (cm) will be measured at Screening only

7.3.3.4 ECOG performance status

ECOG status will be assessed at below visits:

- Screening
- Pre-dose of Day 1 of each treatment cycle
- End-of-treatment visit
- 30- and 90-day safety follow-up.

It will be documented in the eCRF.

7.3.3.5 Clinical laboratory assessments

The sponsor will be provided with a list of local laboratory normal ranges before shipment of the investigational medical product. Any change in laboratory normal ranges during the study should also be forwarded to the sponsor or designated CRO.

Blood samples will be taken from subjects who have been fastened for at least 6 hours and prior to administration of KN046. All routine laboratory analyses will be performed at a laboratory facility local to the study site and relevant results essential for subject management decisions (eg, hematology, biochemistry, liver function test) must be available and reviewed prior to administration of KN046. The

report of the results must be retained as a part of the subject’s medical record or source documents and documented on the eCRF. Blood samples for the test listed in Table 18 will be taken from subjects during Screening, Treatment Phase, the end-of-treatment visit, and 30- and 90-day safety follow-up visits at the time point specified in Assessment Schedule (Table 1 and

). In case of clinically relevant abnormal laboratory test (eg, AST, ALT and/or total bilirubin) requiring additional laboratory draws, unscheduled laboratory tests will be performed.

Endocrine function and urinalysis tests will be assessed at the time points specified in the Schedule of Assessments (Table 1 and Table 2).

Blood sampling in order to exclude active infection with hepatitis B and hepatitis C virus and tuberculosis will be performed at Screening. If confirmation of a subject's childbearing potential is necessary, a FSH level will also be performed at Screening.

If a subject has a clinically significant abnormal laboratory test value that is not present at baseline, the test will be repeated weekly and the subject will be followed until the test value has returned to the normal range or the Investigator has determined that the abnormality is chronic or stable.

Table 15 Laboratory Test Items

Blood chemistry	Hematology	Coagulation	Urinalysis
Alkaline phosphatase	RBC count	Prothrombin time (PT)	pH
Alanine aminotransferase	Hematocrit	Activated partial thromboplastin time (aPTT)	Specific gravity
Aspartate aminotransferase	Hemoglobin	International Normalized Ratio (INR)	Glucose
		Fibrinogen (FIB)<br>Fibrinogen (FIB)	
		Fibrinogen degradation product （FDP）<br>Fibrinogen degradation product （FDP）	
Albumin	Mean corpuscular hemoglobin (MCH)	D-dimer<br>D-dimer	Protein
Total bilirubin	Mean corpuscular hemoglobin concentration (MCHC)	**Hormone levels**	Ketones
Serum urea nitrogen	Mean corpuscular volume (MCV)	Follicle stimulating hormone (if applicable)	Urine blood cells
Blood calcium	Platelet counts	TSH	24-hour protein urine
Chloride	White blood cell (WBC) count with differential	FT4	
Creatinine	Neutrophil count	TT3	**Viral test**
Glucose	Lymphocyte count<br>Lymphocyte count	FT3	HBsAg (if it is positive, HBV DNA should be added)
Lactate dehydrogenase	Monocyte count	ACTH	HCV antibody (if it is positive, HCV RNA should be added)

γ-Glutaminotransferase	Basophil count		HIV antibody
Total protein	Eosinophil count	**Pregnancy test, as applicable**	
Blood potassium		HCG	**Troponin I or T**
Blood sodium		Urine HCG	

1. If urine protein is ≥ 2+ (dipstick method) in urinalysis, 24-hour urine should be collected for detection of total protein.

7.3.4 Pharmacokinetics assessments

The PK sampling plan is presented in Tables 3. The PK sample collection for this study is a sparse sample collection and will be analyzed by a non-linear mixed effects model, which will be detailed in a separate PK/pharmacodynamic analysis plan.

PK blood sample collection should be recorded on the appropriate eCRF page, including the exact date and clock time of KN046 administration and blood sample collection.

Samples will be processed, labeled, stored, and shipped as detailed in the Laboratory Manual. PK blood samples will be analyzed and tested by the central laboratory.

If the PK samples and immunogenicity (anti-KN046 antibodies) samples are scheduled to be collected at the same time, all of these samples should be collected at the same time with the exact collection time of each sample recorded.

7.3.5 Immunogenicity assessment

The immunogenicity of KN046 will be evaluated by detecting ADAs and NADAs. The detection schedule for ADAs is presented in Table 3. Subjects who are positive for ADAs will be further tested for antibody titers.

The immunogenicity samples should be collected, processed, standardized, stored, and shipped as specified in the laboratory manual. Immunogenicity testing will be performed at the central laboratory.

7.3.6 Biomarker assessments

7.3.6.1 Tumor Tissue Biomarker Assessment

It is to submit formalin-fixed, paraffin-embedded tissues in subjects who have signed optional informed consent for biomarker analysis. For China sites, this requirement is optional. Tumor tissues recently obtained (biopsy specimens from non-radiation areas within 24 months) during Screening period will be used for TMB, gene expression profile (GEP), multiplex IHC, PD-L1 expression and tumor infiltrating lymphocyte (TIL) analysis. Block, or 12 unstained tumor slides is acceptable. These analyses will be performed in a central laboratory with prioritization of PD-L1 expression → multiplex IHC, TIL→ TMB/GEP. Please refer to Lab manual provided by the

Sponsor or its designated central lab for details on sample collection, process, standardization, storage and transportation.

PD-L1 positive (tumor and interstitial cells) will be determined using immunohistochemical (IHC) methods developed by the central laboratory. Tumor cell analysis included the percentage of tumor cells at each staining intensity level. It is also possible to determine PD-L1 expression in tumor microenvironment cells (eg, immune cells/infiltrating lymphocytes). The number and location of tumor-infiltrating lymphocytes, such as, but not limited to, immunohistochemistry for detection of ICOS+CD4 T cells, natural killer cells, B cells, macrophages, neutrophils, bone marrow-derived suppressor cells, fibroblasts and vascular structures, CD8 and Foxp3 regulatory T cells. TMB will be determined by the NGS method; GEP will be determined by RNA nanostring method.

Tissue Collection: A biopsy should be collected at Screening unless tissue (blocks or slides) from an archival specimen (biopsy or surgery) is available and was obtained no more than 24 months prior to Screening. Endoscopic biopsies, core needle biopsies, excisional biopsies, punch biopsies, and surgical specimens are suitable. Fine needle aspiration biopsies are not suitable. Biopsies are only to be obtained from safely accessible tumor tissue/sites.

Provision of Samples: 1° Priority: tumor-containing FFPE tissue block; 2° Priority: if the tumor-containing FFPE tissue block cannot be provided in total, sections from this block should be provided that are freshly cut (within 1 week), 4~6 μm thick, and mounted on SuperFrost Plus microscope slides. Subjects should be encouraged to provide as many slides as possible, and preferably, approximately 12~15 slides should be provided in total. However, if this is not possible, a minimum of 5-10 slides is required for PD-L1 expression and multiplex IHC analysis.

Tissue Processing: Cancer tissues should be fixed in 10% neutral buffered formalin, paraffin-embedded, and routinely processed for histological evaluation. Formalin substitutes are not suitable as a fixative.

Sample and Tissue Repository: Biomarker samples may be stored beyond the end of the study and utilized at a later time jointly with samples from other studies in order to investigate actions of KN046 or aspects of the disease under study, and the commercial development of the companion diagnostic kit.

7.3.6.2 Serum biomarker assessment

Each subject is required to provide a serum sample during the screening period for BRCA and HRD mutation analysis.

8 ADVERSE EVENT ASSESSMENT AND RECORD

8.1 Adverse event definition

8.1.1 Adverse event

An AE is any untoward medical occurrence in a subject or clinical investigation subject administered a pharmaceutical product, which does not necessarily have a causal relationship with this treatment. An AE can therefore be any unfavorable and unintended sign (including an abnormal laboratory finding), symptom, or disease temporally associated with the use of a medicinal product, whether or not considered related to the medicinal product. In cases of surgical or diagnostic procedures, the condition / illness leading to such a procedure is considered as the AE rather than the procedure itself.

The investigator is required to grade the severity / intensity of each AE. Investigator will reference the NCI-CTCAE v5.0. This is a descriptive terminology that can be used for AE reporting. A general grading (severity / intensity) scale is provided at the beginning of the referenced document, and specific event grades are also provided. If the severity / intensity of a particular AE is not specifically graded by the guidance document, the investigator is to revert to the general definitions of Grade 1 through Grade 5 and use his or her best medical judgement.

The 5 general grades are:

- Grade 1: Mild; asymptomatic or mild symptoms; clinical or diagnostic observations only; intervention not indicated;
- Grade 2: Moderate; minimal, local or noninvasive intervention indicated; limiting age-appropriate instrumental activities of daily living (ADL);
- Grade 3: Severe or medically significant but not immediately life-threatening; hospitalization or prolongation of hospitalization indicated; limiting self-care ADL;
- Grade 4: Life-threatening consequences or disabling; urgent intervention indicated;
- Grade 5: Death related to AE.

If events classified as CTCAE grade 4 or higher, serious adverse events will be considered with special attention. However, a laboratory abnormality with a severity / intensity of Grade 4, such as anemia or neutropenia, is considered serious only if the condition meets one of the serious criteria described below.

In the case of death, the primary cause of death (the event leading to death) should be recorded and reported as a SAE. “Fatal” will be recorded as the outcome of this respective event; death will not be recorded as a separate event. Only if no cause of death can be reported (eg, sudden death, unexplained death), the death per se might be reported as a SAE.

Abnormal laboratory findings and other abnormal investigational findings (eg, on an ECG trace) should not be reported as AEs unless they are associated with clinical signs and symptoms, lead to treatment discontinuation or are considered otherwise medically important by the investigator. In addition, required specific corrective therapy or meets definition of an SAE should also be reported as AE. If an abnormality fulfils these criteria, the identified medical condition (eg, anemia) must be reported as the AE rather than the abnormal value itself whenever possible.

Medical conditions present at the initial study visit that do not worsen in severity or frequency during the study are defined as baseline medical conditions, and are NOT to be considered AEs.

Disease progression can be considered as a worsening of a patient's condition attributable to the disease for which the investigational product is being studied. It may be an increase in the severity of the disease under study and/or increases in the symptoms of the disease. The development of new, or progression of existing metastasis to the primary cancer under study should be considered as disease progression and not an AE. Events, which are unequivocally due to disease progression, should not be reported as an AE during the study.

The Investigator will assess causal relationship between investigational product and each AE, and answer 'yes' or 'no' to the question 'Do you consider that there is a reasonable possibility that the event may have been caused by the investigational product?'

Not related: Not suspected to be reasonably related to the study treatment. The AE could not medically (pharmacologically/clinically) be attributed to the treatment under study in this protocol. A reasonable alternative explanation must be available.

Related: Suspected to be reasonably related to the study treatment. The AE could medically (pharmacologically/clinically) be attributed to the investigational medicinal product (IMP) under study in this protocol.

The following factors should be considered when deciding if there is a "reasonable possibility" that an AE may have been caused by the drug.

• Time Course. Exposure to suspect drug. Has the subject actually received the suspect drug? Did the AE occur in a reasonable temporal relationship to the administration of the suspect drug?

• Consistency with known drug profile. Was the AE consistent with the previous knowledge of the suspect drug (pharmacology and toxicology) or drugs of the same pharmacological class? OR could the AE be anticipated from its pharmacological properties?

• Dechallenge experience. Did the AE resolve or improve on stopping or reducing the dose of the suspect drug?

• No alternative cause. The AE cannot be reasonably explained by another aetiology such as the underlying disease, other drugs, other host or environmental factors.

• Rechallenge experience. Did the AE reoccur if the suspected drug was reintroduced after having been stopped? The Sponsor would not normally recommend or support a rechallenge.

• Laboratory tests. A specific laboratory investigation (if performed) has confirmed the relationship?

A "reasonable possibility" could be considered to exist for an AE where one or more of these factors exist.

In contrast, there would not be a "reasonable possibility" of causality if none of the above criteria apply or where there is evidence of exposure and a reasonable time course but any dechallenge (if performed) is negative or ambiguous or there is another more likely cause of the AE.

In difficult cases, other factors could be considered such as:

• Is this a recognized feature of overdose of the drug?

• Is there a known mechanism?

Ambiguous cases should be considered as being a " reasonable possibility " of a causal relationship unless further evidence becomes available to refute this. Causal relationship in cases where the disease under study has deteriorated due to lack of effect should be classified as no reasonable possibility.

8.1.2 Serious adverse event

An SAE is any untoward medical occurrence that at any dose:

Results in death;

Is life-threatening.

Note: The term "life-threatening" in this definition refers to an event in which the subject is at risk of death at the time of the event; it does not refer to an event that hypothetically might cause death if it were more severe.

Requires inpatient hospitalization or prolongation of existing hospitalization;

Results in persistent or significant disability/incapacity;

Is a congenital anomaly/birth defect;

Is otherwise considered as medically important.

Important medical events that may not result in death, be life-threatening, or require hospitalization may be considered as SAEs when, based upon appropriate medical judgment, they may jeopardize the subject or may require medical or surgical intervention to prevent 1 of the outcomes listed in this definition. Examples of such events include allergic bronchospasm requiring intensive treatment in an emergency room or at home, blood dyscrasias or convulsions that do not result in inpatient hospitalization, or the development of drug dependency or drug abuse.

For the purposes of reporting, any suspected transmission of an infectious agent via an investigational medicinal product is also considered a serious adverse reaction and all such cases should be reported in an expedited manner as described in this Section.

Elective hospitalizations to administer, or to simplify study treatment or study procedures (e.g., an overnight stay to facilitate chemotherapy and related hydration therapy application) are not considered as SAEs. However, all events leading to unplanned hospitalizations or unplanned prolongation of an elective hospitalization (e.g., undesirable effects of any administered treatment) must be documented and reported as SAEs.

8.1.3 AE of interest

Grade 2 or greater infusion-related reactions are considered AE of interest and must be reported to sponsor within 24 hours of identification by the investigator according to the reporting procedure of SAE (See Section 8.1.2).

Grade 3 or 4 CPK increase, myositis or myocarditis are considered AE of interest and must be reported within 24 hours of identification by the investigator according to the reporting procedure of SAE (See Section 8.1.2).

Myasthenia Gravis is considered AE of interest and must be reported within 24 hours of identification by the investigator according to the reporting procedure of SAE (See Section 8.1.2).

Liver function abnormal meeting below criteria are considered AE of interest and should be reported following the procedure of SAE as stated in Section 8.1.2.

- ALT or AST > 8 x ULN;
- ALT or AST > 5 x ULN for more than 2 weeks;
- ALT or AST > 3 x ULN and (total bilirubin > 2 x ULN or INR > 1.5);
- ALT or AST > 3 x ULN with the appearance of fatigue, nausea, vomiting, right upper quadrant pain or tenderness, fever, rash, and/or eosinophilia (>5%).

8.2 Methods of recording and assessing adverse events

At each study visit, the subject will be queried on changes in his/her condition. During the reporting period of the study, any unfavorable changes in the subject's condition will be recorded as AEs, whether reported by the subject or observed by the investigator.

Complete, accurate, and consistent data on all AEs experienced for the duration of the AE Reporting Period (as defined in Section 8.3) will be reported on an ongoing basis in the appropriate section of the eCRF. Among these AEs and all SAEs must also be documented and reported using the appropriate Report Form.

It is important that each AE report includes a description of the event, its duration (onset and resolution dates [/times "/times" to be completed when it is important to assess the time of AE onset

relative to the recorded treatment administration time]), its severity, its relationship with the study drug, any other potential causal factors, any treatment given or other action taken (including dose modification or discontinuation of the investigational medicinal product), and its outcome. In addition, serious cases should be identified and the appropriate seriousness criteria documented.

8.3 Definition of the adverse event reporting period

All AEs will be collected from time of signature of inform consent through 30 days following the last dose of study drug or the date of subject initiates new anticancer therapy, whichever is earlier. All SAE and treatment related adverse event will be collected from the time of informed consent through 90 days following the last dose of study drug. However, if an investigator learns of any SAE, after 90-day safety follow-up period, and she/he considers there is a reasonable possibility that the event is related to the study drug, the investigator should report to sponsor.

8.4 Procedure for reporting serious adverse events

If an SAE occurs, sponsor is to be notified via paper Serious Adverse Reporting form within 24 hours of investigator awareness of the event. This timeframe also applies to additional new information (follow-up) on previously forwarded SAE reports. For all SAEs, the investigator is obligated to pursue and provide information to sponsor in accordance with the timeframes for reporting specified above. In addition, an investigator may be requested by Sponsor to obtain specific additional follow-up information in an expedited fashion. In general, this will include a description of the AE in sufficient detail to allow for a complete medical assessment of the case and independent determination of possible causality. Information on other possible causes of the event, such as concomitant medications and illnesses must be provided. In the case of a patient death, a summary of available autopsy findings must be submitted as soon as possible to Sponsor or its designated representative.

8.5 Monitoring of subjects with adverse events

Adverse events are recorded and assessed continuously throughout the study and are assessed for final outcome at the 30-day safety follow-up (30 days after the last dose of study therapy). After the 30-day safety follow-up visit, all new and ongoing SAEs and all treatment-related nonserious AEs ongoing at the 90-day safety follow-up visit(90 days after the last dose of study therapy) must be monitored and followed-up by the investigator until resolution or stabilization or until the outcome is known, unless the subject is documented as “lost to follow up”. Reasonable attempts to obtain this information must be made and documented. The investigator is also to ensure that any necessary additional therapeutic measures and follow-up procedures are performed and recorded in the eCRF.

8.6 pregnancy and in utero drug exposure

If, following initiation of the study drug, it is subsequently discovered that a study subject is pregnant or may have been pregnant at the time of study exposure, including during at least 24 weeks after product administration, the investigator must immediately notify the Sponsor Medical Monitor/designee of this event and complete and forward a Pregnancy Reporting Form to sponsor within 24 hours of awareness of the event and in accordance with SAE reporting procedures

described in Section 8.4. In the event of a pregnancy in a subject occurring during the course of the study, the subject must be discontinued from KN046 administration immediately.

Follow-up information regarding the course of the pregnancy, including perinatal and neonatal outcome and, where applicable, offspring information must be reported on the Pregnancy Reporting Form.

Any pregnancy that occurs in a female partner of a male study participant should be reported to Jiangsu Alphamab Biopharmaceuticals Co., Ltd. Information on this pregnancy will be collected on the Pregnancy Reporting Form.

Any abnormal outcome must be reported in an expedited manner as described in Section 8.4, while normal outcomes must be reported within 45 days from delivery.

9 STATISTICS

9.1 Statistical methods

All data for all subjects in the study will be presented by SAS. Unless otherwise noted, all data will be evaluated based on actual observations and missing data will not be estimated.

Study results will be summarized using descriptive statistics, i.e., statistics for continuous variables may include mean, median, range, and standard deviation/variability. Qualitative variables will be summarized by count and percentage. The uncertainty of estimates will be assessed by confidence intervals (CIs). The specific analytical methods will be described in the SAP.

9.2 Sample size

30 subjects and 25 subjects will be enrolled into KN046 monotherapy cohort and KN046 combination therapy cohort, respectively, based on the dose regimen. The sample size calculation is based on the Clopper-Pearson method to estimate the 95% confidence interval for ORR.

Table 16 Sample size calculation

Sample size	ORR, %	ORR 95% CI
30（Mono）	5%	(0.82%, 17.2%)
	10%	(2.1%, 26.5%)
	15%	(5.6%, 30.7%)
	20%	(7.7%, 38.6%)
	25%	(12.3%, 42.3%)
	30%	(14.7%, 49.4%)
25（Combination）	40%	(21.1%, 61.3%)
	45%	(24.4%, 65.1%)
	50%	(31.3%, 72.2%)
	55%	(34.9%, 75.6%)
	60%	(38.7%, 78.9%)
	65%	(42.5%, 82.0%)
	70%	(50.6%, 87.9%)
	75%	(54.9%, 90.6%)

9.3 Analysis set

The following analysis sets will be defined in the study:

Safety Set (SAS): all subjects who received at least one (full or partial) dose of study treatment. Subjects will be classified according to the treatment prescribed in the protocol. Unless otherwise stated, SAS will be the default analysis set for all analyses.

Efficacy Analysis Set (EAS): all subjects who received at least one (full or partial) dose of study treatment and had at least one post-baseline tumor imaging assessment. EAS will be used for the analysis of ORR and DOR.

Pharmacokinetic Analysis Set (PAS): all subjects who receive at least one (full or partial) dose of study treatment and provide at least one post treatment KN046 concentration value above the lower limit of quantification (LLOQ) of the assay.

Immunogenicity Analysis Set (IAS): all subjects who receive at least one (full or partial) dose of KN046 and provide at least one post treatment anti-KN046 antibody result.

9.4 Demographic and Other Baseline Characteristics

Demographic and other baseline data, including age, sex, body height, weight, ECOG score, etc. will be presented for each subject in the study report and summarized by dose level using descriptive statistics (continuous data) or frequency tables (categorical data).

9.5 Primary endpoint analysis

The 95% CI for ORR calculated by the Clopper Pearson method will be reported by cohort based on the EAS; and the parameters of the time-related event (DOR) will be calculated using the Kaplan-Meier method by cohort.

9.6 Secondary endpoint analysis

9.6.1 Safety endpoints

Actual cumulative dose and duration of exposure (days), dose intensity (calculated ratio of actual dose to actual treatment duration), relative dose intensity (calculated ratio of dose intensity to planned dose/planned treatment duration), number of dose delays, and treatment discontinuations will be listed and summarized for KN046.

Safety analysis will be performed based on the SS. Descriptive statistical analyses of safety endpoints will be performed by dose level. Safety analyses will be based on the incidence of AEs, TEAEs, irAEs, TRAEs, and changes in vital signs, ECGs, weights, ECOG scores and laboratory values (hematology and serum chemistry). The treatment period is defined as the period from the first dose of KN046 to 90 days after the last dose of KN046, or until 1 day before the initiation of a new anti-tumor therapy, whichever occurs first.

9.6.1.1 Adverse events

All AEs will be coded using the Medical Dictionary for Regulatory Activities (MedDRA) and their severities will be graded according to the NCI-CTCAE Version 5.0 Toxicity Rating Scale. AEs will be summarized by PTs and SOCs with their severities and relationship to study drugs described.

Analyses of AEs will include, but not be limited to:

- All AEs
- SAEs
- TEAEs
- TRAEs
- irAEs
- AEs leading to treatment discontinuation
- AEs leading to death.

9.6.1.2 Tolerability

The tolerability of subjects to the study drugs will be evaluated by the number of subjects with dose interruption, delay, and discontinuation in each cohort. Reasons for dose interruption, delay, and discontinuation will be listed by cohort, and descriptive statistical analysis will be performed on the frequency of dose interruption, delay and discontinuation by cohort.

9.6.1.3 Laboratory abnormalities

Laboratory results will be graded according to NCI-CTCAE Version 5.0. Parameters that cannot be graded will be classified as decreased/normal/increased according to the normal range of laboratory tests.

Test values for each laboratory test (e.g., hematology, serum chemistry, etc.) will be listed by laboratory parameter, subject, and dose level. Then, the frequency of obvious laboratory abnormalities (Grade 3 or 4 laboratory test abnormalities judged by the NCI-CTCAE Version 5.0) will be described by parameters, number of treatment cycles and dose levels; and the frequency of all laboratory abnormalities will be described by parameter, worst grade (according to the NCI-CTCAE Version 5.0), and dose level.

The obvious laboratory abnormalities (Grade 3 or 4 laboratory test abnormalities judged by the NCI-CTCAE Version 5.0) will be listed. For the parameters that can be categorized using the CTCAE Version 5.0, laboratory data can be summarized by changes in grade as listed in the tables. The parameters that could not be categorized according to the CTCAE Version 5.0 are divided into 3 groups (decreased/normal/increased) according to the normal range, and the data will be listed to statistically analyze the frequency of occurrence.

9.6.1.4 Other safety data (physical examination, ECG, vital signs)

All ECG parameters, including the QT interval corrected according to the Fridericia method (QTc interval) for each subject, will be listed, and descriptive statistical analysis will be performed on the change from baseline for each parameter by dose level and evaluation time. The correlations between cohorts as well as PK parameters (e.g., C_{max}, AUC_{0-t}) and changes in QTc will be presented graphically.

Blood pressure, pulse, respiratory rate, body temperature, and body weight will be listed for all subjects, and changes from baseline for each value will be presented and statistically described.

All physical examination findings will be listed.

9.6.2 Efficacy endpoints

Efficacy endpoints related to tumor assessment will be analyzed based on the EAS, and PFS and OS will be analyzed based on the SAS. The 95% CIs calculated by the Clopper Pearson method will be reported by cohort for individual proportions (CBR); the parameters (including median and 95% CIs) of time-related events (DOR, PFS, OS) calculated by the Kaplan-Meier method will be reported by cohort.

The specific items and methods of analysis will be detailed in the SAP.

9.6.3 Pharmacokinetic Endpoints

PK analysis will be performed based on the measured KN046 plasma concentrations and actual blood sample collection time points, and individual PK parameters will be calculated, including AUCtau, ss, C_{max}, $C_{trough, ss}$, CL, V, and $T_{1/2}$, etc. Descriptive statistical analysis will be performed on PK parameters, including mini, max, median, arithmetic mean, geometric mean, and coefficient of variation (CV)%. The specific analyses and the corresponding methods will be detailed in a separate PK/pharmacodynamic analysis plan.

9.6.4 Correlation between biomarkers and clinical response endpoints

The correlation between PD-L1 expression levels and TMB, as well as clinical efficacy indicators, will be analyzed based on the ROC curve analysis of PD-L1 expression levels for clinical efficacy indicators (ORR, CBR, PFS rate, OS rate).

TIL, HRD, and BRCA1/2 mutation status will be categorized (e.g., high/medium/low TIL; BRCA1/2 mutation negative vs positive; homologous recombination deficiency gene mutation negative vs positive), and statistical analysis will be conducted for clinical efficacy indicators within each category. For individual proportions (ORR, CBR, PFS rate, OS rate), the 95% confidence interval will be calculated using the Clopper Pearson method. For time-related events (DOR), parameters (including median and 95% confidence interval) will be calculated using the Kaplan-Meier method according to the treatment cohort.

9.6.5 Immunogenicity endpoints

The frequency of anti-KN046 ADAs and NADAs in each dose group will be listed by cohort and descriptive statistical analysis will be performed; for ADA-positive subjects, the ADA titers will also be listed. The frequency of low/medium/high titer ADAs will be listed for each cohort.

9.7 Other endpoints

The correlation between PK and safety will be based on Logistic regression analysis of $AUC_{tau, ss}$, $C_{trough, ss}$ and C_{max} and safety endpoints (irAEs ≥ Grade 3, etc.). The data for $AUC_{tau, ss}$ and C_{max} will be derived from the results of the population PK analysis; the data for C_{trough} could be derived from the results of the population PK analysis or from the observed data. It will be described in detail and reported in a separate PK/pharmacodynamic analysis plan.

The composition of TILs in the tumor tissue before and after KN046 treatment will be listed, showing the changes relative to baseline, and statistically described.

10 DATA COLLECTION AND MANAGEMENT

10.1 Data confidentiality

Information about study subjects will be kept confidential and managed under the applicable laws and regulations.

10.2 Site monitoring

Before study initiation, at a site initiation visit or at an investigator's meeting, Jiangsu Alphamab Biopharmaceuticals Co., Ltd.personnel or a designated CRO will review the protocol and eCRFs with the investigators and their staff. During the study, the field monitor will visit the site regularly to check the protocol and to Good Clinical Practice, the progress of enrollment, and to ensure that study treatment is being stored, dispensed, and accounted for according to specifications. Key study personnel must be available to assist the field monitor during these visits.

The investigator must maintain source documents for each patient in the study, consisting of case and visit notes (hospital or clinical medical records) containing demographic and medical information, laboratory data, electrocardiograms, and the results of any other tests or assessments. All information recorded on eCRFs must be traceable to source documents in the patient's file. The investigator must also keep the original signed informed consent form (a signed copy is given to the subject).

The investigators must give the field monitor access to all relevant source documents to confirm their consistency with the eCRF entries. Jiangsu Alphamab Biopharmaceuticals Co., Ltd. monitoring standards require full verification for the presence of informed consent, adherence to the inclusion/exclusion criteria and documentation of SAEs. Additional checks of the consistency of the source data with the eCRFs are performed according to the study-specific monitoring plan.

10.3 Data collection

Electronic Data Capture (EDC) is used for the study. The designated investigator staff will enter the data required by the protocol into the eCRFs. The eCRFs have been built using fully validated secure web-enabled software that conforms to 21 CFR Part 11 requirements. Investigator site staff will not be given access to EDC system until they have been trained. Automatic validation programs check for data discrepancies in the eCRFs and, allow modification or verification of the entered data by the investigator staff.

The Principle Investigator (PI) is responsible for assuring that the data entered into the eCRF is complete, accurate, and that entry and updates are performed in a timely manner.

PK, ADA and biomarker (blood and tissue) samples drawn during the course of the study will be collected from the investigator sites and analyzed by Jiangsu Alphamab Biopharmaceuticals Co., Ltd. assigned laboratory or contracted central laboratories. The site staff designated by the investigator will enter the information required by the protocol onto the PK, ADA and biomarker sample collection eCRFs. The designated laboratory's requisition forms that will be printed on 2-part paper, one copy of the requisition form forwarded to the central laboratory along with the

corresponding samples with required information (eg, study code, subject ID etc) and the other copy retained by the site. The field monitor will review the relevant eCRFs for accuracy and completeness and will work with the site staff to adjust any discrepancies as required. The field monitor will also review the requisition forms for completeness.

Imaging data will be collected by a designated imaging CRO and electronically transferred to Jiangsu Alphamab Biopharmaceuticals Co., Ltd.

10.4 Database management and quality control

EDC is used for the study. Jiangsu Alphamab Biopharmaceuticals Co., Ltd. personnel or designated CRO will review the data entered by investigational staff for completeness and accuracy. Electronic data queries stating the nature of the problem and requesting clarification will be created for discrepancies and missing values and sent to the investigational site via the EDC system. Designated investigator site staff are required to respond promptly to queries and to make any necessary changes to the data.

Concomitant treatments and prior medications entered into the database will be coded using the WHO Drug Reference List, which employs the Anatomical Therapeutic Chemical classification system. Medical history/current medical conditions and adverse events will be coded using the Medical Dictionary for Regulatory Activities (MedDRA) terminology.

Samples and/or data relating to PK, ADA and biomarker blood/tissue samples will be processed centrally and the results will be sent electronically to Jiangsu Alphamab Biopharmaceuticals Co., Ltd.

At the conclusion of the study, the occurrence of any protocol violations will be determined. Once the data has been verified to be complete and accurate, the database will be declared locked. Authorization is required prior to making any database changes to locked data according to Sponsor SOP.

After database lock, the investigator will receive a CD-ROM or paper copies of the subject data for archiving at the investigational site.

11 ETHICAL AND REGULATORY ASPECTS

11.1 Responsibilities of the investigator

The investigator is responsible for the conduct of the study at the site and will ensure that the study is performed in accordance with this clinical trial protocol, the ethical principles outlined in the Declaration of Helsinki, ICH GCP and any other applicable regulations. The investigator must ensure that only subjects who have given informed consent are included in the study.

According to USA Code of Federal Regulations Part 54.2(e), for studies conducted in any country that could result in a product submission to the USA FDA for marketing approval and could contribute significantly to the demonstration of efficacy and safety of an investigational medicinal product (which are considered “covered clinical studies” by the FDA), the investigator and all subinvestigators are obliged to disclose any financial interest which they, their spouses or their dependent children may have in the Sponsor or the Sponsor’s product under study. This information is required during the study and for 12 months following completion of the study.

11.2 Independent ethnic committee or institutional review board

Prior to commencement of the study at a given site, the Clinical Trial Protocol will be submitted together with its associate documents, such as ICF, to the responsible IEC/IRB for its favorable opinion/approval. The written favorable opinion/approval of the IEC/IRB will be filed in the investigator site file, and a copy will be filed with the Sponsor or its designated CRO.

The study must not start at a site before the Sponsor has obtained written confirmation of favorable opinion/approval from the concerned IEC/IRB. The IEC/IRB will be asked to provide documentation of the date of the meeting at which the favorable opinion/approval was given, and of the members and voting members present at the meeting. Written evidence of favorable opinion/approval that clearly identifies the study, the Clinical Trial Protocol version, the subject information and ICF version reviewed should be provided. Where possible, copies of the meeting minutes should be obtained.

Amendments to the study will also be submitted to the concerned IEC/IRB before implementation in case of substantial changes. Relevant safety information will be submitted to the IEC/IRB during the course of the study in accordance with applicable regulations.

11.3 Health authorities

The Clinical Trial Protocol and any applicable documentation (eg, IMP dossier, subject information, and the ICF) will be submitted or notified to the health authorities in accordance with all applicable local or national regulations for each site.

11.4 Informed consent

An unconditional prerequisite for a subject's participation in the study is his/her written informed consent. The subject's written informed consent to participate in the study must be given before any study-related activities are performed. Separate specific informed consent of biomarkers will also be provided to subjects who are willing to participate in these optional procedures, which refers to the extraction and analysis of DNA or RNA expression from blood and/or tumor biopsy in order to better understand how gene(s) or gene expression profile may affect the efficacy of KN046.

Adequate information must therefore be given to the subject by the investigator before informed consent is obtained. A subject information sheet in the local language and prepared in accordance with the Note to Guidance on GCP will be provided by the Sponsor for the purpose of obtaining informed consent. In addition to providing this written information to a potential subject, the investigator or his/her designate will inform the subject verbally of all pertinent aspects of the study. The subject will be given sufficient time to read the information and the opportunity to ask questions and to request additional information and clarification.

Where the information is provided by the investigator, the ICF must be signed and personally dated by the subject and the investigator. The signed and dated declaration of informed consent will remain at the investigator's site, and must be safely archived by the investigator so that the forms can be retrieved at any time for monitoring, auditing and inspection purposes. A copy of the signed and dated information and ICF should be provided to the subject prior to participation.

Whenever important new information becomes available that may be relevant to the subject's consent, the written subject information sheet and any other written information provided to subjects will be revised by the Sponsor or designee and be submitted again to the IEC/IRB for review and favorable opinion. The agreed, revised information will be provided to each subject in the study for signing and dating. The investigator will explain the changes to the previous version. The subject will be given sufficient time to read the information and the opportunity to ask questions and to request additional information and clarification about the changes.

11.5 Subject identification and privacy

A unique subject identifier number will be assigned to each subject, immediately after informed consent has been obtained. This number will serve as the subject's identifier in the study as well as in the clinical study database. All subject data collected in the study will be stored under the appropriate subject number. Only the investigator will be able to link study data to an individual subject via an identification list kept at the site. For each subject, original medical data will be accessible for the purposes of source data verification by the monitor, audits and regulatory inspections, but subject confidentiality will be strictly maintained.

Data protection and privacy regulations will be observed in capturing, forwarding, processing, and storing subject data. Subjects will be informed accordingly, and will be requested to give their consent on data handling procedures in accordance with applicable regulations.

Blood and tumor tissue samples for biomarker analysis will be stored for up to 10 years after study completion. During this time, samples may be reanalyzed for newly identified markers or with new

or improved technology. After 10 years, the samples will be destroyed or fully anonymized or a new IEC/IRB approval and informed consent will be requested to keep the samples for an additional period. If tumor tissue remains, the site will be notified and the tumor tissue will be returned to the site upon request. If the site does not request the return of the tumor tissue, it will be destroyed.

11.6 Clinical trial insurance and compensation

Insurance coverage will be provided for each country participating to the study. Insurance conditions shall meet good local standards, as applicable.

12 TRIAL MANAGEMENT

12.1 Case report form handling

The investigator or qualified designee by the investigator will be responsible for entering trial data in the eCRF provided by the Sponsor or delegated CRO and follow the data entry guidelines. It is the investigator's responsibility to ensure the accuracy of the data entered in the eCRF and to sign the case report form.

The data will be entered into a validated database. The Sponsor or its delegated CRO will be responsible for data review and processing in accordance with the Sponsor or its delegated CRO's data management procedures.

Database lock will only occur once quality control procedures and quality assurance procedures, if applicable, have been completed. Copies of the eCRF will be provided to the investigator at the completion of the trial.

12.2 Source data and subject files

The investigator must keep a file on paper or electronically for every subject in the trial. The file must contain below demographic and medical information for the subject:

- Subject's full name, date of birth, gender, race, ethnicity, height and body weight;
- Medical history and oncology history;
- Concomitant diseases;
- Prior and concurrent medications;
- Site number and subject number;
- Dates for informed consent (entry into the trial) and visit;
- Any medication examinations and clinical findings predefined in this trial protocol;
- All AEs;
- Date and reason that the subject withdraws from the trial or investigational medicinal product;
- Any other documents containing source data. This includes original printouts of data recorded or generated by automated instruments, CT or MRI scan images, ECG recordings, and laboratory listings etc. Such documents must include at least the subject number and the date of the procedure was performed.

12.3 Investigator site file and archiving

The investigator will be provided with and investigator site file upon initiation of the trial. This file will contain all documents necessary for the conduct of the trial and will be updated and completed throughout the trial. It must be available for review by the Monitor, and must be ready for Sponsor audit and for inspection by Health Authorities during and after the trial. It must be safely archived for at least 15 years after the end of the trial. The documents to be archived include the Subject Identification List and signed subject ICFs. If archiving of the Investigator Site File is no longer possible at the site, the investigator must notify the Sponsor.

All original subject files or medical records must be stored at the site for the longest possible time permitted by the applicable regulations or as per ICH GCP guidelines, whichever is longer. In any case, the investigator should ensure that no destruction of medical records is performed without the written approval of the Sponsor.

12.4 Monitoring, quality assurance and inspection by health authorities

The trial will be monitored in accordance with the ICH Note for Guidance on GCP. The Monitor will perform visits to the trial site at regular intervals.

Representatives of the Sponsor Quality Assurance unit or a designated organization, and health authorities must be permitted to inspect all trial-related documents and other materials at the site, including the Investigator Site File, the completed eCRF, the trial drug, and the subject's medical records.

The clinical trial protocol, data capture procedure and the handling of the data will be subject to independent quality assurance. Audits may be conducted at any time during or after the trial to ensure the validity and integrity of the trial data.

12.5 Changes to the clinical trial protocol

Changes to the clinical trial protocol will be documented in written protocol amendments. Major (substantial, significant) amendments will usually require submission to the Health Authorities and to the relevant IEC / IRB for approval or favorable opinion. In such cases, the amendment will be implemented only after approval or favorable opinion has been obtained.

Minor (nonsubstantial) protocol amendments, including administrative changes, will be filed by the Sponsor and at the site. They will be submitted to the relevant IEC / IRB or to Health Authorities only where requested by pertinent regulations.

Any amendment that could have an impact on the subject's agreement to participate in the trial requires the subject's informed consent prior to implementation (See Section 10.4).

12.6 Clinical trial report and publication policy

The first publication will be a publication of the results of the analysis of the primary endpoint(s) that will include data from all trial sites.

The investigator will inform the Sponsor in advance about any plans to publish or present data from the trial. Any publications and presentations of the results, for example, abstracts, oral presentations etc, either in while or in part by investigators or their representatives will require presubmission review by the Sponsor.

The Sponsor will not suppress or veto publications, but maintains the right to delay publication in order to protect intellectual property rights.

13 REFERENCE

1) Mahoney, K.M., P.D. Rennert, and G.J. Freeman, Combination cancer immunotherapy and new immunomodulatory targets. *Nat Rev Drug Discov*. 2015; 14(8): 561-84.

2) Kathleen M. Mahoney, Paul D. Rennert and Gordon J. Freeman. Nature Reviews: *Drug Discovery*, Volume 14, August 2015.

3) Ipilimumab Package Insert. July 2018.

4) Jedd D. Wolchok, Vanna Chiarion-Sileni, Rene Gonzalez, et al. Overall survival with combined nivolumab and ipilimumab in advanced melanoma. *NEJM* 2017; 377: 1345-1356.

5) Robert J. Motzer, Nizar M. Tannir, David F. McDermott, et al. Nivolumab plus ipilimumab versus sunitinib in advanced renal-cell carcinoma. *NEJM* 2018; 378: 1277-1290.

6) Matthew D. Hellmann, Tudor-Eliade Ciuleanu, Adam Pluzanski, et al. Nivolumab plus ipilimumab in lung cancer with a high tumor mutational burden. *NEJM* 2018; 378: 2093-2104.

7) Wolchok,J.D., Kluger,H., Callahan,M.K., et al. Nivolumab plus ipilimumab in advanced melanoma. *NEJM* 2013; 369: 122–133.

8) Wanqing Chen, Rongshou Zheng, Peter D. Baade, et al. Cancer statistics in China, 2015. *CA Cancer J Clin.* 2016; 66: 115-132.

9) Antonio C. Wolff, M. Elizabeth Hale Hammond, Kimberly H. Allison, et al. HER2 Testing in Breast Cancer: American Society of Clinical Oncology/College of American Pathologists Clinical Practice Guideline Focused Update Summary. *JCO 2018; 14(7): 437-441.*

10) P. Schmid, S. Adams, H.S. Rugo, et al. Atezolizumab and nab-paclitaxel in advanced triple-negative breast cancer. *NEJM 2018; DOI: 10.1056/NEJMoa1809615*.

11) Leisha A. Emens, Cristina Cruz, Joseph Paul Eder, et al. Long-term clinical outcomes and biomarker analyses of atezolizumab therapy for patients with metastatic triple-negative breast cancer. *JAMA Oncol doi:10.1001/jamaoncol.2018.4224.*

12) Adams, et al. ASCO2017. *Abstract 1008*.

13) Emma Nolan, Peter Savas, Antonia N. Policheni, et al. Combined immune checkpoint blockade as a therapeutic strategy for BRCA1-mutated breast cancer. *Sci Transl Med 2017; 9: eaal4922.*

14) Yardley DA.Nab-Paclitaxel mechanisms of action and delivery. *J Control Release 2013;170:365-372.*

15) Gradishar WJ, Tjulandin S, Davidson N, et al. Phase III trial of nanoparticle albuminbound paclitaxel compared with polyethylated castor oil–based paclitaxel in women with breast cancer. *J Clin Oncol 2005; 23: 7794 −803.*

16) Gradishar WJ, Krasnojon D, Cheporov S, et al. Significantly longer progression-free survival with nab-paclitaxel compared with docetaxel as first-line therapy for metastatic breast cancer. *J Clin Oncol 2009; 27: 3611-3619.*

17) European Medicines Agency (EMA). Assessment report for Abraxane (paclitaxel). 2007. http://www.ema.europa.eu/docs/en_GB/document_library/EPAR_Public_assessment_report/human/000778/WC500020433.pdf (accessed 18 Dec2014).

18) Breast Cancer. NCCN Clinical Practice Guidelines in Oncology. Version 3.2018.

19) Lesley Seymour, Jan Bogaerts, Andrea Perrone, et al. iRECIST: guidelines for response criteria for use in trials testing immunotherapeutics. *The Lancet Oncol 2017; 18: e143-e152*.

20) Cesar August Santa-Maria, Taigo Kato, Jae-Hyun Park, et al. A pilot study of durvalumab and tremelimumab and immunogenomic dynamics in metastatic breast cancer. *Oncotarget 2018; 9 (27): 18985-18996*.

21) Howard SC, Jones DP, Pui CH. The Tumor Lysis Syndrome[J]. New England Journal of Medicine, 2011, 364(19):1844-1854.

14 APPENDICES

Appendix 1 RECIST 1.1

For all subjects, the RECIST tumor response data will be used to determine the subject's response at each visit according to RECIST 1.1 criteria (Eisenhauer et al 2009). It will also be used to determine if and when a subject has a progression according to RECIST 1.1 and also best overall response (BOR).

Below tables summarize the response criteria for target, nontarget, and new lesions per RECIST 1.1 criteria. Further details, including the handling of subjects with only nonmeasurable disease at baseline, e.g., as a result of a protocol violation or due to discordance between the IRC and the Investigator assessment, will be provided in the IRC Charter (in accordance with RECIST 1.1).

Response Criteria for Target, Nontarget and New Lesions

Response Criteria	Evaluation of Target Lesions
Complete response	Disappearance of all non-nodal target lesions. In addition, any pathological lymph nodes assigned as target lesions must have a reduction in short axis to < 10 mm
Partial response	At least a 30% decrease in the sum of diameter of all target lesions, taking as reference the baseline sum of diameters
Progressive disease	At least a 20% increase in the sum of diameter of all measured target lesions, taking as reference the smallest sum of diameter of all target lesions recorded at or after baseline. In addition to the relative increase of 20%, the sum must also demonstrate an absolute increase of at least 5 mm[1]
Stable disease	Neither sufficient shrinkage to qualify for PR or CR nor an increase in lesions which would qualify for PD
Unknown	Progression has not been documented and one or more target lesions have not been assessed or have been assessed using a different method than baseline
**Response Criteria**	**Evaluation of Nontarget Lesions**
Complete response	Disappearance of all nontarget lesions. In addition, all lymph nodes assigned as nontarget lesions must be nonpathological in size (< 10 mm short axis)
Progressive disease	Unequivocal progression of existing nontarget lesions[2,3]
Incomplete response/stable disease	Neither CR or PD
Unknown	Progression has not been documented and 1 or more nontarget lesions have not been assessed or have been assessed using a different method than baseline

Evaluation of New Lesions
• If a new lesion is equivocal, continued therapy and follow-up evaluation will clarify if it represents truly new disease. If repeat scans confirm there is definitely a new lesion, then progression should be declared using the date of the first observation of the lesion.<br>• A lymph node is considered as a "new lesion" and, therefore, indicative of PD if the short axis increases in size to ≥ 10 mm for the first time in the study plus 5 mm absolute increase.<br>• If new disease is observed in a region which was not scanned at baseline or where the particular baseline scan is not available for some reason, this should be considered as a PD.

CR: complete response; IRC: Independent Review Committee; PD: progressive disease; PR: partial response.

[1]Following an initial CR, a PD cannot be assigned if all non-nodal target lesions are not present and all nodal lesions are < 10 mm in size. In this case, the target lesion response is still CR.

[2]To achieve unequivocal progression on the basis of the nontarget disease, there must be an overall level of substantial worsening in nontarget disease such that, even in presence of SD or PR in target disease, the overall tumor burden has increased sufficiently to merit discontinuation of therapy. A modest increase in the size of 1 or more nontarget lesions is usually not sufficient to quality for unequivocal progression status.

[3]Although a clear progression of "nontarget" lesions only is exceptional, in such circumstances, the opinion of the Investigator does prevail and the progression status should be confirmed later on by the IRC.

Overall Response at Each Assessment

Target Lesion	Nontarget Lesion	New Lesion	Overall Response
CR	CR	No	CR[1]
CR	Incomplete response/SD	No	PR[1]
CR, PR, SD	UNK	No	UNK
PR	Non-PD and not UNK	No	PR[1]
SD	Non-PD and not UNK	No	SD[1,2]
UNK	Non-PD, or not UNK	No	UNK[1]
PD	Any	Yes, or No	PD
Any	PD	Yes, or No	PD
Any	Any	Yes	PD

CR: complete response; IRC: Independent Review Committee; PD: progressive disease; PR: partial response; SD: stable disease; UNK: unknown.

[1]This overall response also applies when there is no nontarget lesion identified at baseline.

[2]Once confirmed PR or CR is achieved, all subsequent assessments are considered as PR or CR until PD occurs.

1.1.1 Best overall response

The best overall response is the best response recorded from the start of the treatment until disease progression/recurrence (taking as reference for PD the smallest measurements recorded since the treatment started). In general, the patient's best response assignment will depend on the achievement of both measurement and confirmation criteria.

The best overall response will usually be determined from response assessments undertaken while on treatment. However, if any assessments occur after treatment withdrawal the protocol should specifically describe if these will be included in the determination of best overall response and/or whether these additional assessments will be required for sensitivity or supportive analyses. **As a default, any assessments taken more than 28 days after the last dose of study therapy will not be included in the best overall response derivation.** If any alternative cancer therapy is taken while on study any subsequent assessments would ordinarily be excluded from the best overall response determination. If response assessments taken after withdrawal from study treatment and/or alternative therapy are to be included in the main endpoint determination, then this should be described and justified in the protocol.

This study requires a response PR or CR, changes in tumor measurements must be confirmed by repeat assessments that should be performed not less than 4 weeks after the criteria for response are first met.

The best overall response for each patient is determined from the sequence of overall (lesion) responses according to the following rules:

- CR = at least two determinations of CR at least 4 weeks apart before progression where confirmation required or one determination of CR prior to progression

- PR = at least two determinations of PR or better at least 4 weeks apart before progression (and not qualifying for a CR) where confirmation required or one determination of PR prior to progression
- SD = at least one SD assessment (or better) > 6 weeks after start of trial treatment (and not qualifying for CR or PR).
- PD = progression ≤ 12 weeks after start of trial treatment (and not qualifying for CR, PR or SD).

  Note: discontinuation due to ‘Disease progression’ or ‘death due to study indication’ is considered as PD even if this was not accompanied by documentation of PD based on tumor measurements. Subjects with symptoms of rapidly progressing disease without radiologic evidence will be classified as progression only when clear evidence of clinical deterioration is documented and/or subjects discontinued due to ‘Disease progression’ or death due to study indication.

- UNK = all other cases (i.e. not qualifying for confirmed CR or PR and without SD after more than 6 weeks or early progression within the first 12 weeks).

Overall lesion responses of CR must stay the same until progression sets in, with the exception of a UNK status. A subject who had a CR cannot subsequently have a lower status other than a PD, e.g. PR or SD, as this would imply a progression based on one or more lesions reappearing, in which case the status would become a PD.

Once an overall lesion response of PR is observed (which may have to be a confirmed PR depending on the study) this assignment must stay the same or improve over time until progression sets in, with the exception of an UNK status. However, in studies where confirmation of response is required, if a subject has a single PR (≥ 30% reduction of tumor burden compared to baseline) at one assessment, followed by a <30% reduction from baseline at the next assessment (but not ≥ 20% increase from previous smallest sum), the objective status at that assessment should be SD. Once a confirmed PR was seen, the overall lesion response should be considered PR (or UNK) until progression is documented or the lesions totally disappear in which case a CR assignment is applicable.

Example: In a case where confirmation of response is required the sum of lesion diameters is 20 cm at baseline and then 14 cm - 15 cm - 14 cm - 16 cm - 16 cm at the subsequent visits. Assuming that non-target lesions did not progress, the overall lesion response would be PR - SD - PR - PR - PR. The second assessment with 14 cm confirms the PR for this subject. All subsequent assessments are considered PR even if tumor measurements decrease only by 20% compared to baseline (20 cm to 16 cm) at the following assessments.

If the subject progressed but continues study medication, further assessments are not considered for the determination of best overall response.

The primary analysis of the best overall response in this study will be based on the investigator calculated overall lesion responses.

Based on the subjects' best overall response during the study, the following rates are then calculated:

- Overall response rate (ORR) is the proportion of subjects with a best overall response of CR or PR.
- Disease control rate (DCR) is the proportion of subjects with a best overall response of CR or PR or SD.
- Clinical benefit rate (CBR) is the proportion of subjects with a best overall response of CR or PR or SD $\geq$ 24 week.

1.1.2 Time to event variable

1.1.2.1 Progression-free survival

Progression-free survival (PFS) is the time from date of start of trial treatment to the date of event defined as the first documented progression or death due to any cause. If a subject has not had an event, progression-free survival is censored at the date of last adequate tumor assessment.

1.1.2.2 Overall survival

All subjects in this study should be followed until death or until subject has had adequate follow-up time as specified in the protocol whichever comes first. The follow-up data should contain the date the subject was last seen alive / last contacted, the date of death and the reason of death ("Study indication" or "Other").

Overall survival (OS) is defined as the time from date of start of trial treatment to date of death due to any cause. If a subject is not known to have died, survival will be censored at the date of last contact.

1.1.2.3 Duration of response

The analysis following variables should be performed with much caution when restricted to responders since treatment bias could have been introduces. There have been reports where a treatment with a significantly higher response rate had a significantly shorter duration of response but where this probably primarily reflected selection bias which is explained as follows: It is postulated that there are two groups of subjects: a good risk group and a poor risk group. Good risk subjects tend to get into response readily (and relatively quickly) and tend to remain in response after they have a response. Poor risk patients tend to be difficult to induce a response, may have a longer time to respond, and tend to relapse quickly when they do respond. Potent agents induce a response in both good risk and poor risk patients. Less potent agents induce a response mainly in good risk patients only. This is described in more detail by Morgan (1988).

It is recommended that an analysis of all subjects (both responders and non-responders) be performed whether or not a responders only descriptive analysis is presented. An analysis of responders should only be performed to provide descriptive statistics and even then interpreted with caution by evaluating the results in the context of the observed response rates. If an inferential comparison between treatments is required, this should only be performed on all subjects (i.e. not

restricting to “responders” only) using appropriate statistical methods such as the techniques described in Ellis et al (2008). Duration of response in this study will only be calculated in subjects whose response has been confirmed.

Duration of overall response (CR or PR): For subjects with a CR or PR (which may have to be confirmed the start date is the date of first documented response (CR or PR) and the end date and censoring is defined the same as that for time to progression.

1.1.2.4 Time to response

Time to overall response (CR or PR) is the time between date start of trial treatment until first documented response (CR or PR). The response needs to be confirmed subsequently.

In this study, subjects who did not achieve a response (which may have to be a confirmed response) will be censored using the following option:

- At maximum follow-up (i.e. FPI to LPLV used for the analysis) for subjects who had a PFS event (i.e. progressed or died due to any cause). In this case the PFS is the worst possible outcome as it means that the subject cannot subsequently respond.

1.1.2.5 Definition of start and end dates for time to event variables

Assessment date

For each assessment (i.e. evaluation number), the assessment date is calculated as the latest of all measurement dates (e.g. CT-scan) if the overall lesion response at that assessment is CR/PR/SD/UNK. Otherwise - if overall lesion response is progression - the assessment date is calculated as the earliest date of all measurement dates at that evaluation number.

Start date

Date of start of KN046 is to be used for all definitions in this study. For all “time to event” variables, other than the duration of responses, the date of KN046 treatment start will be used as the start date.

For the calculation of duration of responses, the following start date should be used:

- Date of first documented response is the assessment date of the first overall lesion response of CR / PR (for duration of overall response), when this status is later confirmed.

End date

The end dates which are used to calculate ‘time to event’ variables are defined as follows:

- Date of death (during treatment as recorded on the treatment completion page or during follow-up as recorded on the study evaluation completion page or the survival follow-up page).

- Date of progression is the first assessment date at which the overall lesion response was recorded as progressive disease.

- When there is no documentation of radiologic evidence of progression, and the subject discontinued for ‘Disease progression’ due to documented clinical deterioration of disease, the date of discontinuation is used as date of progression.

- Date of last adequate tumor assessment is the date the last tumor assessment with overall lesion response of CR, PR or SD which was made before an event or a censoring reason occurred. In this case the last tumor evaluation date at that assessment is used. If no post-baseline assessments are available (before an event or a censoring reason occurred) the date of start of KN046 treatment is used.

Example (if protocol defined schedule of assessments is 3 months): tumor assessments at baseline - 3 months - 6 months - missing - missing - PD. Date of next scheduled assessment would then corresponds to 9 months.

- Date of discontinuation is the date of the end of treatment visit.

- Date of last contact is defined as the last date the subject was known to be alive. This corresponds to the latest date for either the visit date, lab sample date or tumor assessment date. If available, the last contact date from that survival follow-up page is used. If no survival follow-up is available, the date of discontinuation is used as last contact date.

1.1.2.6 Censoring reason

In order to summarize the various reasons for censoring, the following categories will be calculated for each time to event variable based on the treatment completion page, the study evaluation completion page and the survival page.

For survival the following censoring reasons are possible:

- Alive

- Lost to follow-up

For PFS and TTP (and therefore duration of responses) the following censoring reasons are possible:

- Ongoing without event

- Lost to follow-up

- Withdrew consent

- Adequate assessment no longer available*

- Event documented after two or more missing tumor assessments

- New cancer therapy added

* This reason is applicable when adequate evaluations are missing for a specified period prior to data cut-off (or prior to any other censoring reason) corresponding to the unavailability of two or more planned tumor assessments prior to the cut-off date. The following clarifications concerning this reason should also be noted:

- This may be when there has been a definite decision to stop evaluation (e.g. reason=Administrative problems on study evaluation completion page), when subjects are not followed for progression after treatment completion or when only UNK assessments are available just prior to data cut-off).

- The reason "Adequate assessment no longer available" also prevails in situations when another censoring reason (e.g. withdrawal of consent, loss to follow-up or alternative anti-cancer therapy) has occurred more than the specified period following the last adequate assessment.

- This reason will also be used for censor in case of no baseline assessment.

Data handling, analysis and reporting plan will be detailed in SAP.

Appendix 2 Guidelines on the management of immune related adverse event

Only the general principles of treatment for relatively common immune-related adverse events are listed below. Other immune-related toxicities, including rare but severe immune-related toxicity treatments (such as immune-related ocular toxicity and central nervous system toxicity) can be found in ESMO (*JBAG Haanen, F. Carbonnel, C. Robert et al. Management of toxicities from immunotherapy: ESMO clinical practice guidelines for diagnosis, treatment and follow-up. Annals of Oncol 2017; 28(S4): 119-142*) and NCCN (*Julie R Brahmer, Christina Lacchetti, Bryan J Schneider et al. Management of immune-related adverse events In patients with immune checkpoint inhibitor therapy: American Society of Clincial Oncology Clinical Practice Guideline. JCO 2018; 36: 1714-1768*). Subjects who have been treated for more than 3 weeks with steroids recommend the use of drugs such as sulfamethoxazole/trimethoprim to prevent opportunistic infections such as pneumocystis.

Management of immune related pneumonitis

CTCAE v5.0 Grade	KN046 Dosing Management	Action and Guidelines	Diagnostic Consideration
**Grade 1**<br>Asymptomatic. Radiographic changes only presenting as ground glass change, non-specific interstitial pneumonia	• Consider delay KN046 treatment	• Monitor symptoms and signs every 2~3 days<br>• Radiologic findings should be followed on serial imaging studies at least every 3 weeks<br>• Consider pulmonary consultation. Perform bronchoscopy if clinically indicated	All attempts should be made to rule out other causes such as metastatic disease, bacterial (eg, Legionella, Mycoplasma) or viral infection
**Grade 2**<br>Mild/moderate new symptoms of dyspnea, cough, chest pain	• Withhold KN046<br>• Treatment with KN046 may be resumed if the event improves to ≤ Grade 1 within 12 weeks and corticosteroids have been reduced to the equivalent of methylprednisolone 10 mg/day orally or less<br>• **Discontinue KN046 if upon rechallenge the patient develops a second episode of ≥ Grade 2 pneumonia**<br>• **Discontinue KN046 if Grade 2 pneumonia lasting for ≥ 4 weeks**	• Rule out other causes such as infectious diseases<br>• Consider pulmonary function tests<br>• Consider pulmonary consultation. Perform bronchoscopy and biopsy/bronchoalveolar lavage (BAL) if clinically indicated<br>• Start empirical antibiotics treatment if suspicion of infection (fever, CRP increased, neutrophil counts increased)<br>• Consider hospitalization and monitor signs and symptoms every day<br>• If no evidence of infection or no improvement in signs and symptoms with	

CTCAE v5.0 Grade	KN046 Dosing Management	Action and Guidelines	Diagnostic Consideration
	**despite appropriate medical intervention**	antibiotics after 48 hours, add in prednisolone 1 mg/kg/day (or equivalent) orally with prolonged taper lasting for at least 4 weeks	
**Grade 3-4**<br>Severe new symptoms; new/worsening hypoxia; life threatening; difficulty in breathing; acute respiratory distress syndrome (ARDS)	• Discontinue KN046	• Perform pulmonary function tests<br>• Pulmonary consultation. Recommend to perform bronchoscopy and biopsy/bronchoalveolar lavage (BAL)<br>• Treat with intravenous (IV) methylprednisolone 2~4 mg/kg/day (or equivalent). When symptoms improve to Grade 1 or less, a high-dose oral steroid (eg, prednisone 1~2 mg/kg/day or equivalent) taper should be started and continued over no less than 4 weeks<br>• If IV steroids followed by high-dose oral steroids does not reduce initial symptoms within 48~72 hours, treat with infliximab at 5 mg/kg once every 2 weeks. Discontinue infliximab upon symptom relief and initiate a prolonged steroid taper over 6~8 weeks. If symptoms worsen during steroid reduction, initiate a retapering of steroid starting at a higher dose of 80 or 100 mg followed by a more prolonged taper with or without administration of infliximab. Addition of mycophenolate mofetil to replace infliximab could be considered if the subject has liver injury<br>• Add prophylactic antibiotics for preventing opportunistic infections	

Management of immune related colitis

CTCAE v5.0 Grade	KN046 Dosing Management	Action and Guidelines	Diagnostic Consideration
**Grade 1**<br>< 4 liquid stools per day over baseline	• No change in KN046 dose	• For diarrhea, treat symptomatically (loperamide, oral hydration, electrolyte substitution, ADA colitis diet and avoid high fiber high lactose diet)<br>• Grade 1 diarrhea that persist for > 1 week should be treated with the addition of oral diphenoxylate hydrochloride and atropine sulfate four times daily and budesonide 9 mg daily<br>• Grade 1 diarrhea that persist for > 2 weeks should consider to add treatment with prednisolone 0.5~1 mg/kg (non-enteric coated)<br>• If diarrhea persists, endoscopy is recommended	All attempts should be made to rule out other causes such as metastatic disease, bacterial or parasitic infection, viral gastroenteritis, or the first manifestation of an inflammatory bowel disease by examination for stool leukocytes, stool cultures, and a Clostridium difficile titer
**Grade 2**<br>4~6 liquid stools per day over baseline, or abdominal pain, or blood in stool or nausea or noctumal episodes	• Withhold KN046 till ≤ Grade 1	• Consultation with gastroenterologist and endoscopy is recommended to confirm or rule out colitis for Grade 2 diarrhea that persists > 1 week or Grade 1~2 diarrhea with rectal bleeding<br>• Grade 2 diarrhea should be treated with the addition of oral diphenoxylate hydrochloride and atropine sulfate four times daily and budesonide 9 mg daily<br>• Grade 2 diarrhea that persists > 1 week or Grade 2 diarrhea with diffuse ulceration and bleeding seen on endoscopy is recommended to add oral prednisolone 0.5~1 mg/kg (or equivalent) with prolonged taper lasting for at least 4 weeks prednisolone. Diffuse ulceration and bleeding seen on endoscopy represents an increased risk	

CTCAE v5.0 Grade	KN046 Dosing Management	Action and Guidelines	Diagnostic Consideration
		for the development of bowel perforation and indicates longer taper of steroid	
**Grade 3-4**<br>≥7 liquid stools per day or life-threatening	• Withhold KN046<br>• KN046 will be permanently discontinued if ≥ Grade 3 colitis is confirmed<br>• Discontinue KN046 if unable to reduce corticosteroid dose to < 10 mg per day prednisone or equivalent within 12 weeks of toxicity	• Require hospitalization<br>• Endoscopy is recommended to confirm or rule out colitis<br>• Grade 3-4 colitis<br>• Systemic corticosteroids should be initiated at a dose of 1~2 mg/kg/day of prednisone or equivalent. When symptoms improve to Grade 1 or less, corticosteroid taper should be started and continued over at least 4 weeks<br>• Grade 3-4 diarrhea<br>• Rule out bowel perforation. Imaging with plain films or computed tomography (CT) can be useful<br>• Consider consultation with gastroenterologist and confirmation biopsy with endoscopy<br>• Treat with intravenous (IV) steroids (methylprednisolone 125 mg) followed by high-dose oral steroids (prednisone 1~2 mg/kg once per day or equivalent). When symptoms improve to Grade 1 or less, steroid taper should be started and continued over no less than 4 weeks. Taper over 6~8 weeks in patients with diffuse and severe ulceration and/or bleeding<br>• If IV steroids followed by high-dose oral steroids does not reduce initial symptoms within 48~72 hours,	

CTCAE v5.0 Grade	KN046 Dosing Management	Action and Guidelines	Diagnostic Consideration
		consider treatment with infliximab upon symptom relief and initiate a prolonged steroid taper over 6~8 weeks. If symptoms worsen during steroid reduction, initiate a retapering of steroids starting at a higher dose of 80 or 100 mg followed by a more prolonged taper with or without administration of infliximab. **Caution:** infliximab is contradicted in patients with bowel perforation or sepsis<br>• If symptoms persist despite the above treatment, a surgical consult should be obtained	

Management of immune related endocrine disorder

Management of immune related colitis

CTCAE v5.0 Grade	KN046 Dosing Management	Action and Guidelines	Diagnostic Consideration
**Grade 1-2**<br>• Hyperthyroidism<br>• Hypothyroidism<br>• Thyroid disorder<br>• Thyroiditis	• No change in KN046 dose	• Monitor thyroid function or other hormone level tests and serum chemistries more frequently (every 3~6 weeks) until returned to baseline values or clinically stable<br>• Replacement of thyroid hormone or thyroid suppression therapy as clinically indicated	All attempts should be made to rule out other causes such as brain metastases, sepsis, and/or severe infection
**Grade 3-4**<br>• Hyperthyroidism<br>• Hypothyroidism<br>• Thyroid disorder<br>• Thyroiditis	• Withhold KN046 treatment until on stable replacement dose as determined by resolution of symptoms and normalization of hormone levels	• Consider endocrine consultation<br>• Replacement of thyroid hormone or thyroid suppression therapy as clinically indicated	
**Grade 1-4**<br>• Adrenal insufficiency<br>• Hypophysitis<br>• Hypopituitarism<br>• Pan-hypopituitarism	• Withhold KN046 treatment until on stable replacement dose as determined by resolution of symptoms and normalization of hormone levels	• Consider endocrine consultation<br>• Thyroid hormone and/or steroid replacement therapy to manage adrenal insufficiency<br>• If Grade 1~2 hypophysitis is considered, pituitary gland imaging should be considered (magnetic resonance imaging [MRIs] with gadolinium and selective cuts of the pituitary can show enlargement or heterogeneity and confirm the diagnosis)<br>• If adrenal crisis occurs (Grade 3~4 hypophysitis with clinically significant adrenal insufficiency and hypotension, dehydration, and electrolyte abnormalities, such as hyponatremia and hyperkalemia), it requires hospitalization and intravenous methylprednisolone should be initiated	

Management of immune related hepatitis

CTCAE v5.0 Grade	KN046 Dosing Management	Action and Guidelines	Diagnostic Consideration
**Grade 1**<br>• ALT or AST>1-3 x ULN<br>• Subjects with baseline ALT or AST increased, toxicity Grading worsened for ≤1	• No change in KN046 dose	• Weekly monitor liver function tests	All attempts should be made to rule out other causes such as medications (eg, statins, antibiotics), alcohol history, viral infections (eg, anti-HAV/HBV/HCV antibody, HEV PCR), other liver diseases (eg, anti-ANA / SMA / LKM / SLA / LP / LCI, iron studies) or metastasis / clot
**Grade 2**<br>• ALT or AST 3-5 x ULN<br>• Subjects with baseline ALT or AST increased, toxicity Grading worsened for ≤1 and ≤5 x ULN	• Withhold KN046	• Monitor liver function tests every 3 days<br>• If no improvement in liver function tests, treat with oral steroids (prednisolone 1 mg/kg/ day)	
**Grade 3-4**<br>• ALT or AST >5 x ULN<br>• With or without total bilirubin increased > 3x ULN	• Discontinue KN046	• Liver biopsy to establish etiology of hepatic injury, if necessary<br>• Treat with high-dose IV prednisolone 2 mg/kg/day or equivalent for 24~48 hours. When symptoms improve to Grade 1 or less, a steroid taper with oral prednisone at 1~2 mg/kg/day (or equivalent) should be started and continue over no less than 4 weeks<br>• If serum transaminase levels do not decrease 48 hours after initiation of systemic steroids, oral mycophenolate mofetil 500 mg every 12 hours may be given. **Caution:** infliximab is not recommended due to its potential for hepatotoxicity<br>• Several courses of steroid tapering may be necessary as symptoms may worsen when the steroid dose is decreased	

Management of immune related skin toxicity

CTCAE v5.0 Grade	KN046 Dosing Management	Action and Guidelines	Diagnostic Consideration
**Grade 1-2**<br>• Skin rash, with or without symptoms, <30% body surface area (BSA)	• No change in KN046 dose	• Symptomatic treatment<br>• Topical glucocorticosteroids, eg betamethasone 0.1% cream or hydrocortisone 1%)<br>• Urea-containing creams in combination with oral antipruritics (eg, diphenhydramine HCl or hydroxyzine HCl)<br>• At investigator's discretion, treatment with oral steroids for Grade 2 events<br>• Treat as Grade 3 skin toxicity if Grade 2 skin toxicity accompanies apparent clinical signs and symptoms	All attempts should be made to rule out other causes such as metastatic disease, infection or allergic dermatitis
**Grade 3**<br>• Rash covers > 30% BSA or Grade 2 with substantial symptoms	• Withhold KN046 treatment	• Dermatology consultation. Consider biopsy to confirm diagnosis<br>• Recommend oral steroids treatment, starting with prednisone 1 mg/kg/day or equivalent. When symptoms improve to Grade 1 or less, steroid taper should be started and continued over no less than 4 weeks	
**Grade 4**<br>• Skin sloughing > 30% BSA with associated symptoms (eg, erythema, purpura, epidermal detachment)	• Discontinue KN046	• Dermatology consultation. Consider biopsy and clinical dermatology photograph<br>• Initiate steroids at methylprednisolone 1~2 mg/kg/day or equivalent. When symptoms improve to Grade 1 or less, steroid taper should be started and continued over no less than 4 weeks	

Management of immune related nephritis

CTCAE v5.0 Grade	KN046 Dosing Management	Action and Guidelines	Diagnostic Consideration
**Grade 1**<br>• Creatinine 1.5 x baseline or > ULN~1.5 x ULN	• No change in KN046 dose<br>• Withhold KN046 if event does not improve with symptomatic treatment	• Symptomatic treatment<br>• Monitor creatinine weekly; resume routine creatinine monitoring per protocol when it returns to baseline	All attempts should be made to rule out other causes such as obstructive uropathy, progression of disease, or injury to other medications
**Grade 2**<br>• Creatinine > 1.5~3 x baseline or > 1.5~3 x ULN	• Withhold KN046 treatment<br>• **Discontinue KN046 treatment if elevations persists > 7 days or worsen after appropriate medical interventions**	• Renal consultation. Consider ultrasound and/or biopsy as appropriate<br>• Treat with systemic corticosteroids at a dose of prednisolone 1~2 mg/kg/day or equivalent. When symptoms improve to Grade 1 or less, steroid taper should be started and continued over no less than 4 weeks<br>• If elevations persist > 7 days or worsen, treat as Grade 3 or 4	
**Grade 3-4**<br>• Creatinine > 3 x baseline or >3 x ULN	• Discontinue KN046	• Renal consultation. Consider ultrasound and/or biopsy as appropriate<br>• Monitor creatinine daily<br>• Treat with systemic corticosteroids at a dose of prednisolone 1~2 mg/kg/day or equivalent. When symptoms improve to Grade 1 or less, steroid taper should be started and continued over no less than 4 weeks	

Management of immune related myocarditis

CTCAE v5.0 Grade	KN046 Dosing Management	Action and Guidelines	Diagnostic Consideration
**Grade 1**<br>• Abnormal in cardiac tests (eg, myocardial enzymes, ECG)	• Discontinue KN046	• Cardiology consultation<br>• Exam ECG, troponin, BNP, cardiac ultrasound and chest X-ray<br>• Immediately initiate high-dose steroid treatment<br>◦ Prednisolone 1~2 mg/kg/day or equivalent<br>◦ Increase steroid dose (eg, methylprednisolone 1 g/day) and add in infliximab, mycophenolate mofetil or anti-thymocyte globulin if symptoms do not promptly respond to steroids<br>• Treat cardiovascular symptoms according to ACC/AHA guidelines<br>• Subjects with troponin or conduction abnormalities requires to be treated within cardiac ICU	All attempts should be made to rule out myocardial infarction, viral myocarditis, infectious heart valve disease etc
**Grade 2**<br>• Abnormal in cardiac tests with mild symptoms			
**Grade 3-4**<br>• Moderate to severe cardiac dysfunction requiring intravenous treatment or life-threatening			

Appendix 3 Guidance on contraception

Birth control methods considered as highly effective.

According to the Clinical Trials Facilitation Group (CTFG) Recommendations Related to Contraception and Pregnancy Testing in Clinical Trials, 2014, methods that can achieve a failure rate of less than 1% per year when used consistently and correctly are considered as highly effective birth control methods, such as:

- Combined (estrogen and progesterone containing) hormonal contraception associated with inhibition of ovulation[1] (oral, intravaginal, transdermal)
- Progesterone-only hormonal contraception associated with inhibition of ovulation[1] (oral, injectable, implantable[2])
- Intrauterine device[2]
- Intrauterine hormone-releasing system[2]
- Bilateral tubal occlusion[2]
- Vasectomized partner[2,3]
- Sexual abstinence[4].

[1]Hormonal contraception may be susceptible to interaction with the IMP, which may reduce the efficacy of the contraception method.

[2]Contraception methods in the context of this guidance are considered to have low user dependency.

[3]Vasectomized partner is a highly effective birth control method provided that the partner is the sole sexual partner of the woman of childbearing potential study participant and that the vasectomized partner has received medical assessment of the surgical success.

[4]In the context of this guidance, sexual abstinence is considered a highly effective method only if defined as refraining from heterosexual intercourse during the entire period of risk associated with the study treatments. The reliability of sexual abstinence needs to be evaluated in relation to the duration of the clinical study and the preferred and usual lifestyle of the subject.

Appendix 4 Eastern Cooperative Oncology Group Performance Status

**Eastern Cooperative Oncology Group Performance Status[1]**	
**Grade**	**Eastern Cooperative Oncology Group**
0	Fully active, able to carry on all predisease performance without restriction
1	Restricted in physically strenuous activity but ambulatory and able to carry out work of a light or sedentary nature, e.g., light house work, office work
2	Ambulatory and capable of all self-care but unable to carry out any work activities. Up and about > 50% of waking hours
3	Capable of only limited self-care, confined to bed or chair > 50% of waking hours
4	Completely disabled. Cannot carry on any self-care. Totally confined to bed or chair
5	Death

[1]Oken MM, Creech RH, Tormey DC et al. Toxicity and Response Criteria of the Eastern Cooperative Oncology Group. Am J Clin Oncol. 1982; 5:649-55.

Appendix 5 Cockcroft-Gault Formula

The estimated creatinine clearance rate (CrCl; mL/min) will be calculated using the Cockcroft-Gault equation based on actual weight.

Conventional – serum creatinine in mg/dL:

in men=((140-age) ×weight)/ (72× [serum creatinine])

in women = 0.85 × creatinine clearance in men

Conventional – serum creatinine in µmol/L:

in men=((140-age) ×weight)/ (0.81× [serum creatinine])

in women = 0.85 × creatinine clearance in men
