# Supplementary material for: The anti-PD-L1/CTLA-4 bispecific antibody KN046 in combination with nab-paclitaxel in first-line treatment of metastatic triple-negative breast cancer: a multicenter phase II trial
Source: Nat Commun. 2024 Feb 3;15:1015. doi: 10.1038/s41467-024-45160-y (PMC10838317; doi:10.1038/s41467-024-45160-y)
Supplement: Supplementary file 3 — Reporting Summary [file 41467_2024_45160_MOESM3_ESM.pdf]

nature portfolio

Corresponding author(s): Binghe Xu
Last updated by author(s): Oct 18, 2023

Reporting Summary

Nature Portfolio wishes to improve the reproducibility of the work that we publish. This form provides structure for consistency and transparency in reporting. For further information on Nature Portfolio policies, see our Editorial Policies and the Editorial Policy Checklist.

Statistics

For all statistical analyses, confirm that the following items are present in the figure legend, table legend, main text, or Methods section.

n/a	Confirmed	
[ ]	[x]	The exact sample size (*n*) for each experimental group/condition, given as a discrete number and unit of measurement
[x]	[ ]	A statement on whether measurements were taken from distinct samples or whether the same sample was measured repeatedly
[ ]	[x]	The statistical test(s) used AND whether they are one- or two-sided<br>*Only common tests should be described solely by name; describe more complex techniques in the Methods section.*
[x]	[ ]	A description of all covariates tested
[x]	[ ]	A description of any assumptions or corrections, such as tests of normality and adjustment for multiple comparisons
[ ]	[x]	A full description of the statistical parameters including central tendency (e.g. means) or other basic estimates (e.g. regression coefficient) AND variation (e.g. standard deviation) or associated estimates of uncertainty (e.g. confidence intervals)
[ ]	[x]	For null hypothesis testing, the test statistic (e.g. *F*, *t*, *r*) with confidence intervals, effect sizes, degrees of freedom and *P* value noted<br>*Give P values as exact values whenever suitable.*
[x]	[ ]	For Bayesian analysis, information on the choice of priors and Markov chain Monte Carlo settings
[x]	[ ]	For hierarchical and complex designs, identification of the appropriate level for tests and full reporting of outcomes
[x]	[ ]	Estimates of effect sizes (e.g. Cohen's *d*, Pearson's *r*), indicating how they were calculated

Our web collection on statistics for biologists contains articles on many of the points above.

Software and code

Policy information about availability of computer code

Data collection	This study used an electronic data acquisition system to create an electronic case report form (eCRF), which was logged online through the Internet for data collection and management.
Data analysis	Statistical analyses were performed using SAS statistical analysis software (version 9.4).

For manuscripts utilizing custom algorithms or software that are central to the research but not yet described in published literature, software must be made available to editors and reviewers. We strongly encourage code deposition in a community repository (e.g. GitHub). See the Nature Portfolio guidelines for submitting code & software for further information.

Data

Policy information about availability of data

All manuscripts must include a data availability statement. This statement should provide the following information, where applicable:

- Accession codes, unique identifiers, or web links for publicly available datasets
- A description of any restrictions on data availability
- For clinical datasets or third party data, please ensure that the statement adheres to our policy

The raw clinical data are protected and are not available due to data privacy laws. The de-identified datasets supporting the findings of this study are available for academic purposes on request from the corresponding author, Binghe Xu for 5 years, with the approval of the Institutional Ethical Committees. A brief summary of the study protocol is available at clinicaltrials.gov. Source data are provided with this paper. The remaining data are available within the Article, Supplementary Information or Source Data file.

Research involving human participants, their data, or biological material

Policy information about studies with human participants or human data. See also policy information about sex, gender (identity/presentation), and sexual orientation and race, ethnicity and racism.

Reporting on sex and gender	27 female adult patients were enrolled in this study.
Reporting on race, ethnicity, or other socially relevant groupings	Totally 27 patients were Asian.
Population characteristics	Patients with histologically confirmed locally advanced unresectable or metastatic TNBC were eligible. Additional inclusion criteria included: ≥18 years old, naïve system treatment for metastatic TNBC, prior chemotherapy in the neoadjuvant/ adjuvant therapy including taxanes was allowed if treatment-free interval (TFI) ≥12 months, measurable disease at baseline according to RECIST v1.1, an Eastern Cooperative Oncology Group performance status (ECOG PS) of 0/1, and adequate organ functions.
Recruitment	From July 2019 to July 2020, 27 female patients with treatment-naïve locally advanced inoperable or metastatic TNBC were enrolled sequentially according to eligibility criteria in the study protocol. 16 patients were assigned to receive KN046 3mg/kg Q2W plus nab-paclitaxel (dose level [DL]-1 group) and the other 11 patients were assigned to receive KN046 5mg/kg Q2W plus nab-paclitaxel (DL-2 groups) sequentially, following a dose escalation part and a dose expansion part afterward.<br>The first 6 enrolled patients were enrolled in the 3mg/kg group (dose Level [DL]-1 group) firstly, The Site Monitoring Committee (SMC) meeting was held as all 6 subjects had completed the 28-day safety observation period. After an evaluation of the safety, initial efficacy, and pharmacokinetic data, decided to increase to the 5mg/kg group (DL-2 group) and also expand the 3mg/kg group at the same time. The 5mg/kg group first enrolled 6 patients, and after the evaluation of the SMC meeting as all subjects had completed the 28-day safety observation period, decided to expand the 5mg/kg group.<br>Written inform consent was obtained from all patients. There were no potential self-selection bias.
Ethics oversight	The present study (KN046-203) was approved by the ethics committee of National Cancer Center/Cancer Hospital, Chinese Academy of Medical Sciences and Peking Union Medical College (Approval No. 2018L02884) and was registered at clinicaltrials.gov (NCT03872791). The study was performed in accordance with the relevant guidelines and regulations. All patients voluntarily participated and provided written informed consent.<br>The study design and conduct complied with all relevant regulations regarding the use of human study participants and was conducted in accordance with the criteria set by the Declaration of Helsinki.

Note that full information on the approval of the study protocol must also be provided in the manuscript.

Field-specific reporting

Please select the one below that is the best fit for your research. If you are not sure, read the appropriate sections before making your selection.

☒ Life sciences ☐ Behavioural & social sciences ☐ Ecological, evolutionary & environmental sciences

For a reference copy of the document with all sections, see nature.com/documents/nr-reporting-summary-flat.pdf

Life sciences study design

All studies must disclose on these points even when the disclosure is negative.

Sample size	The sample size was calculated based on the ORR 95% CI estimated by the Clopper Pearson method, 25 participants were planned to enroll for each dose group.
Data exclusions	No data exclusions in the present study.
Replication	Translational research was not done and replication was not applicable for this clinical trail.

Randomization	The present study was a single-arm trial, no radomization was performed.
Blinding	The present study was a single-arm, open-label trial, blinding was not applicable.

Reporting for specific materials, systems and methods

We require information from authors about some types of materials, experimental systems and methods used in many studies. Here, indicate whether each material, system or method listed is relevant to your study. If you are not sure if a list item applies to your research, read the appropriate section before selecting a response.

Materials & experimental systems

n/a	Involved in the study	
☒	☐	Antibodies
☒	☐	Eukaryotic cell lines
☒	☐	Palaeontology and archaeology
☒	☐	Animals and other organisms
☐	☒	Clinical data
☒	☐	Dual use research of concern
☒	☐	Plants

Methods

n/a	Involved in the study	
☒	☐	ChIP-seq
☒	☐	Flow cytometry
☒	☐	MRI-based neuroimaging

Clinical data

Policy information about clinical studies

All manuscripts should comply with the ICMJEguidelines for publication of clinical research and a completedCONSORT checklist must be included with all submissions.

Clinical trial registration	NCT03872791
Study protocol	The full trial protocol can be accessed at at clinicaltrials.gov.
Data collection	From May 30, 2019 to June 30, 2020, 27 eligible patients with treatment-naïve locally advanced inoperable or metastatic TNBC were enrolled in 8 centers across China, including) National Cancer Center/Cancer Hospital, Chinese Academy of Medical Sciences and Peking Union Medical College; 2) Harbin Medical University Cancer Hospital; 3) Hunan Cancer Hospital; 4) Liaocheng People's Hospital; 5) Sun Yat-Sen Memorial Hospital,6) Liaoning Cancer Hospital & Institute, Cancer Hospital of China Medical University; 7) The First Affiliated Hospital of Xiamen University; 8) Nantong Tumor Hospital.<br>The clinical cutoff date for PFS and OS analyses was on Aug 21, 2022.
Outcomes	The primary endpoints included objective response rate (ORR) and duration of response (DoR) assessed by ndependent Review Committee (IRC). The secondary endpoints included ORR and DoR assessed by investigators, disease control rate (DCR) and clinical benefit rate (CBR) assessed by IRC and investigators, PFS, 1-year/2-year OS rate, and safety/ tolerability.<br>Tumor response was evaluated every 8 weeks according to the Response Evaluation Criteria in Solid Tumors (RECIST) v1.1 [15]. ORR was defined as the portion of patients who have achieved complete response (CR) and partial response (PR). DoR was defined as the time from the date of initial assessed CR or PR until the date of disease progression or death from any cause, whichever occurs first. DCR was defined as the portion of patients who have achieved CR, PR and stable disease (SD). CBR was defined as the portion of patients who have achieved CR, PR and SD ≥ 24 weeks. PFS was defined as the time from the first dose administration to the date of disease progression or cancer-related death, whichever occurs first. OS was defined as the time from the first dose administration to the date of death or last visit.<br>AEs were collected and graded according to the National Cancer Institute Common Terminology Criteria for Adverse Events (CTCAE) v5.0 assessed by IRC.
